# Cost-effectiveness of multidisciplinary care in mild to moderate chronic kidney disease in the United States: A modeling study

**DOI:** 10.1371/journal.pmed.1002532

**Published:** 2018-03-27

**Authors:** Eugene Lin, Glenn M. Chertow, Brandon Yan, Elizabeth Malcolm, Jeremy D. Goldhaber-Fiebert

**Affiliations:** 1 Division of Nephrology, Department of Medicine, Stanford University School of Medicine, Palo Alto, California, United States of America; 2 Center for Health Policy/Center for Primary Care and Outcomes Research, Stanford University School of Medicine, Palo Alto, California, United States of America; 3 Duke University, Durham, North Carolina, United States of America; 4 Division of General Medical Disciplines, Department of Medicine, Stanford University School of Medicine, Palo Alto, California, United States of America; Royal Derby Hospital, UNITED KINGDOM

## Abstract

**Background:**

Multidisciplinary care (MDC) programs have been proposed as a way to alleviate the cost and morbidity associated with chronic kidney disease (CKD) in the US.

**Methods and findings:**

We assessed the cost-effectiveness of a theoretical Medicare-based MDC program for CKD compared to usual CKD care in Medicare beneficiaries with stage 3 and 4 CKD between 45 and 84 years old in the US. The program used nephrologists, advanced practitioners, educators, dieticians, and social workers. From Medicare claims and published literature, we developed a novel deterministic Markov model for CKD progression and calibrated it to long-term risks of mortality and progression to end-stage renal disease. We then used the model to project accrued discounted costs and quality-adjusted life years (QALYs) over patients’ remaining lifetime. We estimated the incremental cost-effectiveness ratio (ICER) of MDC, or the cost of the intervention per QALY gained. MDC added 0.23 (95% CI: 0.08, 0.42) QALYs over usual care, costing $51,285 per QALY gained (net monetary benefit of $23,100 at a threshold of $150,000 per QALY gained; 95% CI: $6,252, $44,323). In all subpopulations analyzed, ICERs ranged from $42,663 to $72,432 per QALY gained. MDC was generally more cost-effective in patients with higher urine albumin excretion. Although ICERs were higher in younger patients, MDC could yield greater improvements in health in younger than older patients. MDC remained cost-effective when we decreased its effectiveness to 25% of the base case or increased the cost 5-fold. The program costed less than $70,000 per QALY in 95% of probabilistic sensitivity analyses and less than $87,500 per QALY in 99% of analyses. Limitations of our study include its theoretical nature and being less generalizable to populations at low risk for progression to ESRD. We did not study the potential impact of MDC on hospitalization (cardiovascular or other).

**Conclusions:**

Our model estimates that a Medicare-funded MDC program could reduce the need for dialysis, prolong life expectancy, and meet conventional cost-effectiveness thresholds in middle-aged to elderly patients with mild to moderate CKD.

## Abstract 2

Using a modeling approach, Eugene Lin and colleageus examine the cost-effectiveness of multi-disciplinary care in mild to moderate chronic kidney disease in the US.

## Author summary

**Why was this study done?:**

- Chronic kidney disease is a major cause of morbidity and mortality in the US.
- Multidisciplinary care—when healthcare providers of different expertise collaborate to treat a single disease—has successfully reduced mortality and the incidence of end-stage renal disease in patients with chronic kidney disease.
- Understanding the economic impact of multidisciplinary care in chronic kidney disease could help policy makers decide whether such a program improves health outcomes in a cost-effective way.

**What did the researchers do and find?:**

- We developed a novel Markov model that accurately simulates the progression of chronic kidney disease to end-stage renal disease and accounts for heterogeneity in patients with chronic kidney disease.
- Multidisciplinary care was cost-effective in most patients with chronic kidney disease and was more cost-effective in patients with higher levels of albuminuria.
- Multidisciplinary care remained cost-effective in the model even if it was substantially less effective or more costly than base case estimates.

**What do these findings mean?:**

- Multidisciplinary care could improve the health of patients with chronic kidney disease at reasonable value for money.
- Policy makers could consider implementing pilot multidisciplinary care programs to formally test their effectiveness and cost-effectiveness.

## Introduction

Chronic kidney disease (CKD) affects approximately 10% of Medicare beneficiaries in the US but accounts for a disproportionate 20% of expenditures [1]. Patients with end-stage renal disease (ESRD) are more costly, representing 1.6% of Medicare beneficiaries and responsible for 7.2% of costs [1]. At the same time, life expectancy is substantially lower in patients with CKD than in the general population [1–3].

Multidisciplinary care (MDC) has been proposed as a way to mitigate the high costs and mortality associated with CKD. It has led to successful outcomes in other settings, including heart failure [4], intensive care [5], and cancer [6]. In CKD, researchers have investigated a variety of strategies, including nurse and advanced practitioner coordinated models [7–12], use of dieticians and social workers [7–9,12,13], and education programs [14–19]. Several systematic reviews have shown that MDC slows CKD progression [20], delays the onset of dialysis, and decreases mortality [21].

Little is known about the cost-effectiveness of MDC in a US CKD population. Although prior studies have suggested that MDC is cost-effective, these studies were performed in other countries and did not use validated models for CKD progression [22,23]. Developing an accurate model is challenging because CKD progression is associated with mortality, and previous studies did not account for this relationship. Furthermore, these studies did not consider heterogeneity in CKD. Many patients with mild to moderate CKD do not progress to ESRD and may not benefit from an intensive disease management program [24]. Determining the subgroups that benefit the most from MDC may help providers more effectively treat vulnerable patients with CKD.

In this study, we performed a cost-effectiveness analysis of a theoretical Medicare MDC program for US populations of differing CKD severity. We did so after developing a novel CKD progression model that incorporates disease heterogeneity and mortality risk. To account for inefficiencies in a nationally funded and broadly applied MDC program, we also tested if more expensive and less effective programs remained cost-effective. We hypothesized that MDC is more cost-effective in patients with more severe CKD. We also hypothesized that even an inefficiently deployed program would be cost-effective by conventional thresholds.

## Methods

### Overview and non-technical modeling summary

To model the cost-effectiveness of MDC in CKD, our analysis involved 3 elements: (1) we constructed and calibrated a CKD progression model; (2) we modeled the cost-effectiveness of MDC; and (3) we performed multiple sensitivity analyses. Because the effectiveness of MDC varies for different types of patients, we performed these analyses in US patients of different ages (45–64, 65–74, and 75–84 years old), sexes (female and male), races (white, black, and other), estimated glomerular filtration rates (eGFRs ranging from 20 to 59 ml/min/1.73 m^2^ in 5-ml/min/1.73 m^2^ increments), and approximate albuminuria levels (urine albumin to creatinine ratio [UACR] 1, 300, 1,000, and 3,000 mg/g).

For our CKD progression model, we used a deterministic Markov model that simulates the disease course of patients with CKD as they experience progressive CKD, ESRD, and death. The model accounts for population-level heterogeneity, including differences in age, sex, and race as well as eGFR and albuminuria. For instance, patients with lower eGFRs and patients with higher levels of albuminuria are more likely to develop ESRD in our model. Similarly, older patients have higher mortality rates in our model. To ensure that our model accurately simulated CKD progression and mortality, we calibrated it to long-term mortality rates and ESRD incidence rates, which we obtained from published literature. We show results from our calibration procedure in S2 Appendix to demonstrate that our progression model accurately reflects published literature for different subpopulations with CKD.

To estimate the cost-effectiveness of a theoretical Medicare-funded MDC program in the US, we used our CKD progression model to simulate the total lifetime costs and outcomes (as measured by quality-adjusted life years [QALYs]) of medical care for patients under MDC versus under usual care. After discounting total accrued costs and QALYs by an annual rate of 3%, we computed the incremental cost-effectiveness ratio (ICER), the difference in discounted costs divided by the difference in discounted QALYs. Conceptually, the ICER is the number of dollars that Medicare must spend on MDC to gain 1 QALY. We assumed that a cost-effective program would have an ICER of no more than $150,000 per QALY, which corresponds roughly to the 2017 inflation-adjusted ICER for dialysis ($129,000 per QALY in 2009) [25].

In the literature, MDC spans different interventions and has been used in patients of varying disease severities. The aims of MDC programs have ranged from slowing the progression of CKD to ESRD, to preventing cardiovascular events such as myocardial infarction and stroke, to optimizing decision-making at the onset of ESRD. We limited our study to MDC programs that aimed to slow the progression of CKD to ESRD. We did not incorporate the reduction of cardiovascular hospitalizations or prevention of acute kidney injury into our model because it is not clear from the literature whether MDC is effective in preventing these outcomes. It is important to note that our study assessed reductions in all-cause mortality and thus implicitly assessed reductions in cardiovascular mortality. However, given the absence of data on the effect of MDC on intermediate cardiovascular endpoints, we were unable to incorporate cardiovascular or other hospitalizations into our cost-effectiveness estimates directly.

Additionally, the literature often conflates MDC programs aimed at slowing the progression of CKD with MDC programs aimed at optimizing dialysis planning through reducing the use of tunneled dialysis catheters and improving the use of home dialysis. However, these programs are operationally distinct and are meant for different populations: the former are typically used in patients with stage 3 and 4 CKD, while the latter are reserved for patients on the cusp of needing dialysis, with late stage 4 or early stage 5 CKD. Our interest was in earlier interventions focused on slowing the progression of CKD to ESRD in patients with stage 3 and 4 CKD.

These MDC programs typically comprise periodic visits with nephrologists, advanced practitioners, educators, dieticians, and social workers, which ramp up in frequency as CKD becomes more severe. We assumed that MDC increased the use of medications and laboratory tests routinely used to manage anemia and derangements in bone-mineral metabolism, common in patients with CKD.

Using a recently published systematic review, we constructed a model that estimated the effectiveness of MDC in reducing mortality and in preventing ESRD [21]. This review, and the published literature in general, provided an estimate of the average effectiveness of MDC across many severities of CKD, without accounting for population-level heterogeneity. To incorporate the clinical intuition that many patients with earlier stages of CKD do not progress to ESRD and thus may not benefit from an MDC program, we assumed that MDC was less effective in patients with less severe CKD.

Because a Medicare-funded MDC program would likely have a large range of effectiveness and cost, we performed a wide array of sensitivity analyses varying our assumptions. We did so by repeating our analyses under different scenarios, where MDC was down to only 25% as effective as the base case or up to 5 times more expensive. We also simulated scenarios where MDC did not have any effect on CKD progression. For each scenario, we performed a probabilistic sensitivity analysis, which allowed us to produce cost-effectiveness acceptability curves and 95% confidence intervals.

Below, we describe the technical specifications of our modeling technique.

### Analysis 1: CKD progression model, construction, and calibration

#### Target population and setting

We limited our study to US patients because we investigated a Medicare-based intervention. Since managing advanced CKD and ESRD is substantially different from managing mild to moderate CKD, we limited our analysis to CKD stages 3 and 4.

To better understand MDC’s effects on CKD subpopulations, we divided our population by age (45–64, 65–74, and 75–84 year olds), sex (female and male), race (black, white, and other), eGFR (5-ml/min/1.73 m^2^ increments from 20 to 59 ml/min/1.73 m^2^), and approximate albuminuria level (UACR roughly 1, 300, 1,000, and 3,000 mg/g). We studied middle-aged and elderly patients because they represent nearly all Medicare beneficiaries with CKD. Even though patients 85 years and older are an important subset of Medicare beneficiaries, we excluded them because data on CKD progression and associated mortality are sparse in this subpopulation, making projections less reliable. We chose to study 75–84 year olds separate from 65–74 year olds because they have different outcomes and because many in the former group do not progress to ESRD prior to death [26,27]. We combined 45–54 year olds with 55–64 year olds because they do not constitute a large proportion of Medicare beneficiaries with CKD.

#### Study perspective and time horizon

We took Medicare’s perspective as our reference case because it ultimately absorbs the majority of ESRD costs in the US [1]. By limiting our study population to Medicare beneficiaries with CKD, this perspective is equivalent to taking the healthcare sector perspective, since Medicare beneficiaries remain on Medicare after developing ESRD. In accordance with the recommendations from the Second Panel on Cost-Effectiveness in Health and Medicine, our analysis incorporated all aspects of healthcare sector effects and costs, including longevity effects, health-related quality of life effects, future related and unrelated healthcare costs, and out-of-pocket costs [28,29].

We considered using the societal perspective as a second reference case. However, the societal perspective requires cost estimates that are not readily available in the literature. For instance, the added cost due to caregiver burden from MDC is not known and would require additional assumptions. Likewise, it is unclear whether MDC would improve patient productivity through improved health or reduce patient productivity through increased time spent interacting with healthcare providers. Even though we ultimately decided not to include the societal perspective, we varied the costliness of MDC widely in sensitivity analysis, and this variation likely captures additional costs that would be incorporated into a societal perspective.

Since CKD progression can take years, we modeled patients over their remaining lifetime.

#### CKD progression model

We developed a deterministic Markov model of CKD progression for each subpopulation (see S1 Appendix for modeling and calibration details). For each model, we allowed age and eGFR to vary over time, while sex and race remained constant. We accounted for albuminuria at baseline but did not incorporate longitudinal changes because we did not have sufficient data to model changes over time. Specifically, we created individual models for each combination of sex, race, and starting level of albuminuria. Within each of these models, we allowed probabilities to change depending on age and eGFR. This allowed us to incorporate population-level heterogeneity in our analysis and to separate patients with a high probability of developing ESRD (e.g., those with high levels of albuminuria and low eGFRs) from patients with a low probability of developing ESRD (e.g., those with no albuminuria and relatively high eGFRs).

**Fig 1:**
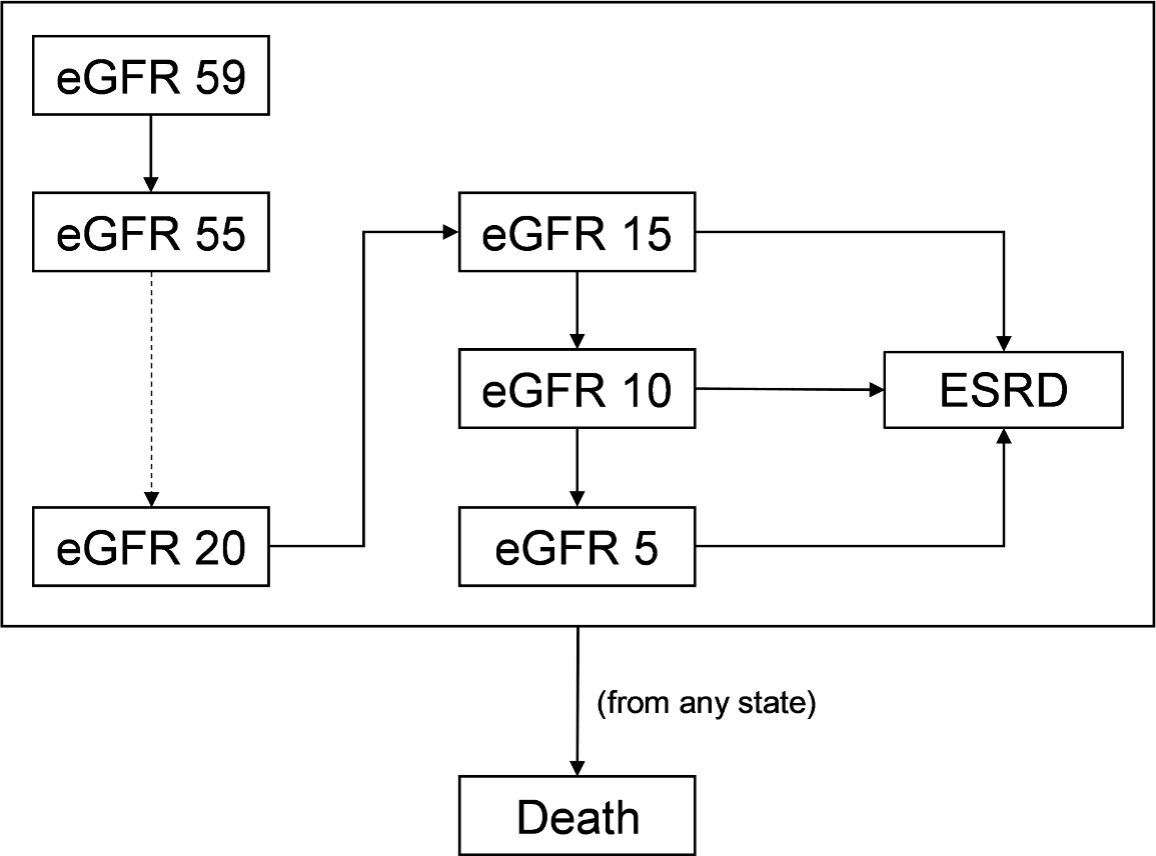
Markov model for simulating chronic kidney disease (CKD) progression. We modeled progression of CKD using levels of estimated glomerular filtration rate (eGFR). For simplicity, we modeled each level of eGFR in increments of 5 ml/min/1.73 m^2^. Each cycle, patients have a probability of staying at the same eGFR level or dropping to the next eGFR. Between eGFRs of 5 and 15 ml/min/1.73 m^2^, patients also have the possibility of progressing to end-stage renal disease (ESRD). We modeled ESRD as a single health state. Death can occur at any point.

Our model simulated CKD progression by modeling changes to eGFR through monthly cycles (Fig 1). Although CKD progression is a continuous process, we made 3 simplifications to improve the identification of model parameters and to reduce the risk of overfitting. First, we used eGFR decrements of 5 ml/min/1.73 m^2^. We did so because CKD progression is slow in most patients (a recent study reported a decline in eGFR of 4.8 ml/min/1.73 m^2^ per year in the upper tertile of patients) [30] and because health-related outcomes likely do not change materially at smaller eGFR decrements. Second, we did not allow patients to skip stages in a cycle given how slowly patients progress. Third, our model does not accommodate improvements in eGFR, even though kidney function can improve in a minority of patients [31]. We did this because Markov models simulate the expected outcome for the average patient, and CKD is progressive on average. Notably, our model was still able to capture population-level heterogeneity despite these assumptions because we modeled each subgroup separately. For instance, the majority of patients with low levels of albuminuria do not develop ESRD in their lifetime [32,33]. Because we modeled patients with different levels of albuminuria separately, populations at low risk for developing ESRD (e.g., those with no albuminuria) have a low ESRD incidence rate when compared to populations at high risk for developing ESRD (e.g., those with UACR of approximately 3,000 mg/g).

Once patients reach an eGFR of 15 ml/min/1.73 m^2^, they face the possibility of developing ESRD. Since many patients do not require renal replacement therapy at this eGFR, our model also allows progression to a minimum of 5 ml/min/1.73 m^2^ prior to developing ESRD [34]. ESRD represents many health states that have substantially different costs and outcomes, such as hemodialysis with a tunneled dialysis catheter, hemodialysis with a permanent vascular access, peritoneal dialysis, and kidney transplant. In our study, we modeled ESRD as a single health state. We did so because we limited our study to MDC programs aimed only at slowing CKD progression. Although MDC has been used to impact ESRD-related decisions, including the use of tunneled dialysis catheters, peritoneal dialysis, and transplant, we did not include these types of MDC in our analysis, and thus we did not incorporate this degree of granularity into our model.

At every health state, patients have a probability of death. To more accurately reflect expected lifetime health outcomes and costs, our model employed a standard half-cycle correction.

#### Calibration strategy

Directly computing transition probabilities is challenging because CKD progression and mortality are associated. Prior models have not considered this, likely inflating the risk of death for patients with more severe CKD [22,23].

To address this concern, we calibrated each model to long-term probabilities of mortality and progression to ESRD (Table 1). We calculated the 2-year and 5-year risk of developing ESRD as a function of age, sex, eGFR, and albuminuria, using a model developed by Tangri et al. [32]. For long-term probabilities of death, we obtained baseline mortality risks (conditional on age, sex, and race) from the Centers for Disease Control and Prevention (CDC) life tables [35] and applied hazard ratios based on eGFR and albuminuria [2]. We used the United States Renal Data System (USRDS), a database of Medicare claims for patients with ESRD, to estimate the proportion of patients starting hemodialysis with a tunneled catheter and the mortality risk of patients with ESRD [36].

**Table 1:** Base case parameter inputs for model calibration targets, MDC effectiveness, healthcare costs, and QALYs.

Parameter	Point estimate (95% CI)	Source
**Model calibration targets**		
Probability of developing ESRD	*	Tangri et al. [32]
Probability of mortality without CKD	^†^	CDC life tables [35]
Hazard ratios for mortality in CKD	^‡^	van der Velde et al. [2]
Probability of mortality in ESRD	^†^	USRDS [36]
**MDC effectiveness, overall**		
Odds ratio of mortality	0.62 (0.44, 0.88)	Wang et al. [21]
Odds ratio of developing ESRD	0.59 (0.38, 0.92)	Wang et al. [21]
**MDC effectiveness, discounted**		
CKD stage 3	25%^§^	Expert opinion
CKD stage 4	50%^§^	Expert opinion
CKD stage 5	100%^§^	Expert opinion
**Cost of usual care**		
Cost of CKD	^†^	USRDS [36]
Cost of ESRD	^†^	USRDS [36]
**Cost of MDC, annual**		
First-time visit	$273.11^§^	Levin et al. [16], CMS fee schedule [37]
CKD stage 3	$797.10^§^	Levin et al. [16], CMS fee schedule [37]
CKD stage 4	$1,484.02^§^	Levin et al. [16], CMS fee schedule [37]
CKD stage 5	$2,074.40^§^	Levin et al. [16], CMS fee schedule [37]
Medications (25% of patients with eGFR ≤ 30 ml/min/1.73 m^2^)	$4,186.46^§^	Expert opinion, CMS fee schedule [37]
Laboratory tests (25% of patients with eGFR ≤ 45 ml/min/1.73 m^2^)	$877.88^§^	Expert opinion, CMS fee schedule [37]
**QALYs**		
CKD	0.80 (0.70, 0.90)	Wyld et al. [38]
ESRD	0.71 (0.62, 0.80)	Wyld et al. [38]

*Varies by age, sex, UACR, and eGFR; see S1 Appendix.

^†^Varies by age, sex, and race; see S1 Appendix.

^‡^Varies by eGFR and UACR; see S1 Appendix.

^§^Confidence interval not relevant.

CDC, Centers for Disease Control and Prevention; CKD, chronic kidney disease; CMS, Centers for Medicare & Medicaid Services; eGFR, estimated glomerular filtration rate; ESRD, end-stage renal disease; MDC, multidisciplinary care; QALY, quality-adjusted life year; UACR, urine albumin to creatinine ratio; USRDS, United States Renal Data System.

Using a constrained Nelder–Mead minimization algorithm, we calculated transition probabilities that minimized the sum of squared percent differences between actual and computed mortality and progression [39]. We chose the Nelder–Mead algorithm because it allowed us to numerically minimize our objective function without relying on partial derivatives, which may be undefined in this type of nonlinear model. We manually inspected each calibrated model to ensure that it yielded reasonable long-term estimates of mortality and progression to ESRD for each subpopulation of interest (S1 Appendix).

### Analysis 2: Modeling the cost-effectiveness of MDC

#### Description of base case

For our base case, we evaluated a theoretical Medicare-funded MDC program for CKD. As described in the overview, we evaluated a program that uses a combination of nephrologists, advanced practitioners, educators, dieticians, and social workers. It increases in intensity through increased provider visits as kidney function worsens and stops once patients progress to ESRD.

Most of the studies included in the systematic review published by Wang et al. did not explicitly describe each activity performed, making it difficult to disentangle the effect of each component within MDC [21]. Furthermore, some of the studies were substantially less extensive, using only nurses or 1 additional provider type. Because of this uncertainty, we made 2 assumptions to ensure that our cost-effectiveness estimates were conservative. First, we took effectiveness estimates from Wang et al., which represent the average effectiveness of MDC. Second, we assumed that the MDC program mirrored the most expensive program documented [16].

Specifically, we assumed that MDC operates as services above and beyond usual CKD care (Table 2). Patients entering the MDC program have an initial, comprehensive 2-hour visit with the multidisciplinary team of healthcare providers. Subsequently, patients attend regular visits with the MDC team. Patients with stage 3 CKD see a nephrologist or advanced practitioner twice a year, a CKD educator once a year, and a dietician and social worker twice a year. Patients with stage 4 CKD see a nephrologist or advanced practitioner 4 times a year, a CKD educator once a year, and a dietician and social worker 4 times a year. Patients with stage 5 CKD see a nephrologist or advanced practitioner 6 times a year, a CKD educator twice a year, and a dietician and social worker 4 times a year.

We compared MDC to usual CKD care.

**Table 2:** Components of the MDC program.

MDC activity	CPT	Cost/CPT	CKD Stage 3		CKD Stage 4		CKD Stage 5	
			Number/year	Cost/year	Number/year	Cost/year	Number/year	Cost/year
**Nephrologist/advanced practitioner**								
New provider visit (60 minutes)*	99205	$209.23	—	—	—	—	—	—
Add-on for chronic care management provider*	G0506	$63.88	—	—	—	—	—	—
Outpatient visit (40 minutes)	99215	$146.43	2	$292.86	4	$585.72	6	$878.58
Complex care management (60 minutes)	99487	$93.67	2	$187.34	4	$374.68	6	$562.02
**CKD education**	G0420	$110.18	1	$110.18	1	$110.18	2	$220.36
**Dietician reassessment (30 minutes)**	97803	$61.02^†^	2	$122.04	4	$244.08	4	$244.08
**Social worker reassessment (30 minutes)**	96151	$42.34^†^	2	$84.68	4	$169.36	4	$169.36
**Total first-time visit cost**				$273.11		$273.11		$273.11
**Total annual recurring costs**				$797.10		$1,484.02		$2,074.40

*These activities make up the first-time visit when initiating MDC.

^†^These costs are computed for 30 minutes of time. Medicare reimburses CPT codes 97803 and 96151 at a rate per 15 minutes of time, and the Medicare Physician Fee Schedule indicates reimbursement at half the displayed cost per 15 minutes.

CKD, chronic kidney disease; CPT, Current Procedural Terminology; MDC, multidisciplinary care.

#### Estimating the effectiveness of MDC

Precisely estimating the program’s effectiveness is difficult because the quality of published studies is limited. The Wang et al. study showed that MDC programs were effective in CKD populations overall [21]. However, most of the included studies were observational or single-center randomized trials. Although the majority were published in the last decade, some were published 10 to 20 years ago. Furthermore, none of the studies stratified the effect of MDC by CKD stage, even though in aggregate, they included patients with stage 3 through 5 CKD.

Given these limitations, we discounted the effectiveness of MDC in earlier CKD stages, using the intuition that MDC is less effective in milder forms of CKD. We first calibrated a 100% effective MDC program to odds ratios from Wang et al.: 0.62 for mortality and 0.59 for progression to ESRD over a 4.9-year follow-up (Table 1) [21]. To adjust for a specific effectiveness of MDC, we took the differences in transition probabilities between the 100% effective MDC model and usual care and discounted these differences by the assumed effectiveness. In our base case, we assumed that MDC was 25% as effective as the literature in stage 3 CKD, 50% as effective in stage 4 CKD, and 100% as effective in stage 5 CKD. We applied the same level of effectiveness for all subgroups within a specific CKD stage.

We were unable to incorporate estimates of patient adherence to MDC in our model because prior studies, including those identified in Wang et al. [21], did not identify the proportion of patients that did not adhere to treatment. Implicitly, our initial estimates of MDC’s effectiveness incorporate the average rate of adherence reported in the literature. It is likely that a nationally implemented program would have a lower rate of adherence than that reported from controlled trials. Our uncertainty of the program’s effectiveness and of patients’ adherence prompted us to vary these assumptions widely in sensitivity analyses.

To make results more tangible, we computed hazard ratios for death and progression to ESRD using 10,000 simulated people and estimated a Cox proportional hazards model for each outcome.

#### Estimating costs

We estimated healthcare costs in US dollars and inflation adjusted them to their January 2017 value [40]. To estimate the annual cost of usual CKD and ESRD care, we used 2012 and 2013 Medicare Parts A and B claims data from the USRDS [36]. We used the 2016 annual data report from the USRDS to obtain Part D cost estimates [1].

For MDC costs, we identified the Current Procedural Terminology (CPT) codes for each MDC activity and obtained their costs from the 2017 Medicare Physician Fee Schedule (Tables 1 and 2) [37]. This resulted in an initial MDC visit cost of $273.11 and subsequent recurring costs that increased with progression of CKD. In general, published studies did not investigate whether MDC increased other healthcare expenses, such as medications and laboratory tests. For our base case, we assumed that MDC increased the use of erythropoietin and activated vitamin D in 25% of patients with an eGFR of 30 ml/min/1.73 m^2^ and below (Tables 1 and 3). Although many patients receive oral vitamin D, we priced the injectable version because it is more expensive and because we did not have access to oral medication prices in Medicare. We also assumed that MDC increased laboratory testing in 25% of patients with an eGFR of 45 ml/min/1.73 m^2^ and below. We varied these assumptions widely in sensitivity analyses. We discounted accrued costs at an annual rate of 3%.

**Table 3:** Medications and laboratory tests increased by MDC in the base case.

Item	CPT	Cost/dose	Doses/year	Cost/year
**Medications**				
Calcitriol (1 mcg, 3 times weekly)	J0636	$6.25	156	$975.00
Epogen (5,000 units, weekly)	J0885	$68.79	52	$3,577.08
**Laboratory tests**				
Comprehensive metabolic panel	80053	$14.49	4	$57.96
Phosphorus	84100	$6.50	4	$26.00
CBC without differential	85027	$8.87	4	$35.48
Iron	83540	$8.88	4	$35.52
TIBC	83550	$11.99	4	$47.96
Ferritin	82728	$18.70	4	$74.80
iPTH	83970	$56.62	4	$226.48
Vitamin D, 25 OH	82306	$40.61	4	$162.44
Vitamin D, 1,25 OH	82652	$52.81	4	$211.24
**Total medication cost**				$4,552.08
**Total laboratory test cost**				$793.92

CBC, complete blood count; CPT, Current Procedural Technology; iPTH, intact parathyroid hormone; MDC, multidisciplinary care; TIBC, total iron binding capacity.

#### Health outcomes and their valuation

As indicated above, we assumed that the only benefits to MDC were slowing of CKD progression and mortality reduction. Although MDC has been proposed as a way to mitigate the risk of hospitalization due to cardiovascular disease or acute kidney injury, data are mixed on its effectiveness [16–19,21,41–48]. Given this uncertainty, we did not believe our model could accurately capture MDC’s effects on these outcomes. MDC in patients with later stages of CKD has also focused on dialysis planning, with the goal of reducing the likelihood of starting hemodialysis with a tunneled dialysis catheter and increasing the use of home dialysis [21]. Because our intervention focused on stage 3 and 4 CKD, before most patients consider dialysis planning, we assumed the program did not influence tunneled dialysis catheter rates or likelihood of home dialysis use.

We used QALYs from a recent systematic review by Wyld et al. to estimate the utilities associated with CKD and ESRD [38]. This study aggregated all studies that directly measured utilities (using time trade-off or standard gamble), converted health-related quality of life into utilities (EQ-5D, 15D, or SF-6D), or translated short-form surveys (SF-36 or SF-12) into EQ-5D scores [49,50]. Although Wyld et al. did not differentiate among different stages of CKD, the published literature does not show substantial differences in health-related quality of life across different CKD stages [51,52]. We therefore used the estimate of 0.80 QALYs for CKD and 0.71 QALYs for ESRD (Table 1). We discounted accrued QALYs at a rate of 3% per year.

For cost-effectiveness estimates, we computed the ICER, or the difference in total lifetime cost divided by the difference in total lifetime QALYs. We assumed a willingness to pay (WTP) of $150,000 per QALY gained.

### Analysis 3: Sensitivity analyses

#### Adjusting the effectiveness and cost of MDC

To test our effectiveness assumptions, we investigated the cost-effectiveness under different scenarios (Table 4). We varied the effectiveness of MDC using the previously described method of discounting the difference in transition probabilities. First, we studied programs that were 50% and 25% as effective as the base case. This corresponded to MDC programs that were 12.5% and 6.25% as effective as the literature in stage 3 CKD, 25% and 12.5% as effective in stage 4 CKD, and 50% and 25% as effective in stage 5 CKD, respectively. Next, we tested programs that were equally effective across all CKD stages, at 100%, 50%, and 25% of estimates published in Wang et al. [21]. We call these scenarios “non-discounted.” Finally, we investigated programs that decreased only mortality (not progression to ESRD) at 100%, 50%, and 25% the effectiveness of the base case. This corresponded to effectiveness levels (for mortality) of 25%, 12.5%, and 6.25% in stage 3 CKD; 50%, 25%, and 12.5% in stage 4 CKD; and 100%, 50%, and 25% in stage 5 CKD, respectively.

**Table 4:** Sensitivity analyses: Varying the effectiveness and cost of MDC.

Scenario	MDC effectiveness	MDC effect on meds/labs	MDC recurring costs
**Base case**	CKD 3: 25%; CKD 4: 50%; CKD 5: 100%	Increased in 25% of patients	See Table 1
**Effectiveness sensitivity analyses**			
50% of base case	CKD 3: 12.5%; CKD 4: 25%; CKD 5: 50%	*	*
25% of base case	CKD 3: 6.25%; CKD 4: 12.5%; CKD 5: 25%	*	*
100%, non-discounted	All CKD stages: 100%	*	*
50%, non-discounted	All CKD stages: 50%	*	*
25%, non-discounted	All CKD stages: 25%	*	*
Only mortality, base case	CKD 3: 25%; CKD 4: 50%; CKD 5: 100% (only mortality)	*	*
Only mortality, 50% of base case	CKD 3: 12.5%; CKD 4: 25%; CKD 5: 50% (only mortality)	*	*
Only mortality, 25% of base case	CKD 3: 6.25%; CKD 4: 12.5%; CKD 5: 25% (only mortality)	*	*
**Cost sensitivity analyses**			
No change in labs/meds	*	Increased in 0% of patients	*
Increase in labs/meds—10%	*	Increased in 10% of patients	*
Increase in labs/meds—50%	*	Increased in 50% of patients	*
Increase in labs/meds—100%	*	Increased in 100% of patients	*
MDC cost—200%	*	*	200% of base case
MDC cost—500%	*	*	500% of base case

*Same as base case.

MDC, multidisciplinary care; CKD, chronic kidney disease; labs, laboratory tests; MDC, multidisciplinary care; meds, medications.

Although we were unable to directly model patient adherence to MDC therapy in our model, this is a major aspect of an MDC program’s effectiveness. By varying effectiveness levels widely, we implicitly tested the cost-effectiveness of MDC at different levels of adherence.

We also tested our assumptions of the cost. We first varied the proportion of patients receiving more medications and laboratory tests under MDC: 0%, 10%, 50%, and 100% of patients in the MDC arm. Next, we estimated the cost-effectiveness when all aspects of MDC were 2 times and 5 times costlier than the base case.

#### Probabilistic sensitivity analysis

For all scenarios, we conducted probabilistic sensitivity analyses by fitting all parameters to probability distributions so that 95% of the probability density fell within the 95% confidence interval (Table 5). In general, we followed standard practice for modeling these distributions by fitting parameters bounded by 0 and 1 (probabilities, QALYs) to beta distributions, odds ratios (positive numbers) to log-normal distributions, and costs (positive numbers) to gamma distributions [53]. This ensured that 95% of random draws occurred within the 95% confidence interval. We drew 5,000 total samples from these distributions for each subpopulation. We induced correlations while preserving marginal distributions, ensuring that ordered probabilities retained their order for each draw (e.g., patients with more severe kidney disease progress faster than patients with less severe kidney disease) [54].

**Table 5:** Probability distributions for probabilistic sensitivity analysis.

Parameter	Probability distribution
**CKD progression model targets**	
Probability of developing ESRD	Beta
Probability of mortality in CKD	Beta
Probability of mortality in ESRD	Beta
**MDC effectiveness**	
Odds ratio of mortality	Log-normal
Odds ratio of developing ESRD	Log-normal
**Costs of CKD and ESRD**	Gamma
**QALYs of CKD and ESRD**	Beta

CKD, chronic kidney disease; ESRD, end-stage renal disease; MDC, multidisciplinary care; QALY, quality-adjusted life year.

For each probability draw, we solved for control and MDC transition probabilities using the previously described algorithm. We used these distributions to calculate the probability of cost-effectiveness at different WTP thresholds and for different subpopulations. We did not use ICERs to compute these probabilities because negative ICERs are difficult to interpret as they can imply a reduced cost with gain in QALYs (preferred) or an increased cost with a loss of QALYs (not preferred). Therefore, we computed an equivalent measure, the net monetary benefit to determine if a probability draw was cost-effective [55]. For a given WTP threshold, the net monetary benefit is the product of the WTP threshold and the number of QALYs gained minus the cost of the intervention. A positive net monetary benefit implies a cost-effective intervention at that WTP threshold. The probabilistic sensitivity analyses also enabled us to estimate 95% confidence intervals for all costs, QALYs, hazard ratios, and net monetary benefits by taking the 2.5% and 97.5% quantiles.

### Statistical software

We used Matlab version R2016b (MathWorks, Natlick, MA) for modeling. We used Stata version 14.1 (StataCorp, College Station, TX) and SAS version 9.4 (SAS Institute, Cary, NC) to estimate costs and target probabilities.

### Ethics statement

The Stanford University Institutional Review Board approved of this study and the use of the USRDS database (IRB-17804).

All work using the USRDS was conducted under a data use agreement between Dr. Tara Chang and the National Institute of Diabetes and Digestive and Kidney Diseases (NIDDK). An NIDDK officer reviewed the manuscript and approved it for submission.

### CHEERS guidelines

Our study conformed to the Consolidated Health Economic Evaluation Reporting Standards (CHEERS) guidelines (S1 Text) [56].

## Results

### Incremental costs and outcomes

We found that MDC improved health by 0.23 (95% CI: 0.08, 0.42) QALYs per person over usual care, from 1.78 to 2.01 QALYs (Tables 6–9). This translated to hazard ratios of 0.77 (95% CI: 0.63, 0.92) for death and 0.62 (95% CI: 0.43, 0.86) for progression to ESRD (Tables 10–13). The improvement in outcomes was more pronounced for younger patients, with 45–64 year olds seeing a higher increase in QALYs (95% CI: 0.31–0.76) than 75–84 year olds (95% CI: 0.09–0.31). Across different races, sexes, and eGFRs, the gain in health was similar. Patients with higher levels of albuminuria generally gained fewer QALYs than those with lower levels of albuminuria. For example, patients with a UACR of approximately 3,000 mg/g gained fewer QALYs (95% CI: 0.09–0.33) than patients with no albuminuria (UACR approximately 1 mg/g) (95% CI: 0.22–0.76). However, relative gains in QALYs were similar across different levels of albuminuria (a 9% to 33% increase versus a 10% to 19% increase, respectively).

**Table 6:** Cost-effectiveness of MDC by severity of kidney disease.

Characteristic		Change in cost		Change in QALYs		ICER (cost/QALY)	Net monetary benefit^‡^	
eGFR*	UACR^†^	Estimate	95% CI	Estimate	95% CI		Estimate	95% CI
**59**	**1**	$17,419	$10,522, $24,357	0.30	0.10, 0.50	$57,958	$27,662	$4,404, $50,748
** **	**300**	$9,960	$4,040, $15,587	0.20	0.07, 0.35	$49,116	$20,458	$5,420, $37,581
** **	**1,000**	$8,264	$2,923, $13,549	0.16	0.05, 0.29	$50,916	$16,081	$4,047, $31,005
** **	**3,000**	$6,567	$1,881, $13,357	0.13	0.04, 0.29	$52,297	$12,269	$3,187, $30,994
**45**	**1**	$19,746	$11,868, $27,589	0.35	0.12, 0.58	$56,181	$32,975	$5,996, $59,557
** **	**300**	$11,672	$4,222, $18,669	0.25	0.09, 0.43	$46,612	$25,888	$7,768, $46,848
** **	**1,000**	$9,922	$3,087, $16,841	0.20	0.07, 0.37	$48,972	$20,468	$6,017, $39,252
** **	**3,000**	$8,203	$1,811, $17,170	0.16	0.05, 0.37	$50,867	$15,986	$4,819, $38,698
**30**	**1**	$22,565	$13,508, $31,643	0.41	0.14, 0.67	$55,315	$38,626	$7,890, $68,512
** **	**300**	$13,229	$4,506, $21,421	0.29	0.11, 0.50	$45,337	$30,540	$9,914, $55,724
** **	**1,000**	$11,204	$3,082, $19,465	0.23	0.08, 0.43	$48,323	$23,574	$7,867, $46,626
** **	**3,000**	$8,864	$1,310, $19,034	0.18	0.06, 0.41	$50,593	$17,416	$5,846, $42,925
**Overall**		$12,001	$5,098, $19,358	0.23	0.08, 0.42	$51,285	$23,100	$6,252, $44,323

See S1 and S2 Tables for total costs and QALYs under MDC and usual care by severity of kidney disease.

*eGFR units are ml/min/1.73 m^2^.

^†^UACR units are mg/g.

^‡^Net monetary benefit under a willingness to pay threshold of $150,000 per QALY gained.

eGFR, estimated glomerular filtration rate; ICER, incremental cost-effectiveness ratio; MDC, multidisciplinary care; QALY, quality-adjusted life year; UACR, urine albumin to creatinine ratio.

**Table 7:** Cost-effectiveness of MDC by age.

Characteristic			Change in cost		Change in QALYs		ICER (cost/QALY)	Net monetary benefit^‡^	
Age (years)	eGFR*	UACR	Estimate	95% CI	Estimate	95% CI		Estimate	95% CI
**45–64**	**59**	**1**	$45,705	$29,224, $63,054	0.63	0.26, 1.01	$72,432	$48,946	$8,956, $88,450
** **	** **	**300**	$24,619	$14,727, $35,302	0.39	0.18, 0.66	$62,713	$34,265	$10,528, $64,657
** **	** **	**1,000**	$19,912	$11,526, $29,619	0.31	0.14, 0.53	$64,724	$26,234	$8,162, $51,701
** **	** **	**3,000**	$16,471	$8,928, $27,255	0.26	0.11, 0.48	$64,016	$22,123	$6,805, $46,155
** **	**45**	**1**	$49,935	$31,907, $67,544	0.70	0.29, 1.09	$71,577	$54,710	$10,818, $95,834
** **	** **	**300**	$25,620	$15,907, $36,967	0.42	0.19, 0.72	$60,816	$37,571	$11,681, $72,093
** **	** **	**1,000**	$20,641	$12,538, $30,985	0.33	0.15, 0.59	$62,514	$28,887	$9,553, $58,577
** **	** **	**3,000**	$17,736	$10,005, $28,417	0.30	0.13, 0.53	$59,939	$26,649	$8,809, $52,920
** **	**30**	**1**	$53,861	$34,508, $71,675	0.76	0.32, 1.16	$71,156	$59,681	$12,542, $102,628
** **	** **	**300**	$26,374	$16,839, $38,561	0.45	0.20, 0.78	$58,916	$40,774	$12,865, $79,838
** **	** **	**1,000**	$21,109	$13,566, $32,293	0.35	0.17, 0.66	$60,468	$31,255	$10,587, $67,346
** **	** **	**3,000**	$18,325	$10,916, $28,318	0.33	0.14, 0.58	$56,034	$30,730	$10,068, $60,153
**65–74**	**59**	**1**	$21,852	$13,050, $31,003	0.39	0.13, 0.65	$56,279	$36,390	$6,174, $66,417
** **	** **	**300**	$13,074	$4,335, $21,100	0.28	0.10, 0.47	$46,923	$28,720	$8,391, $51,669
** **	** **	**1,000**	$10,811	$2,845, $18,455	0.22	0.07, 0.39	$49,041	$22,257	$6,394, $42,605
** **	** **	**3,000**	$8,980	$1,424, $16,509	0.17	0.05, 0.34	$52,833	$16,516	$4,538, $36,093
** **	**45**	**1**	$24,272	$14,458, $34,275	0.44	0.15, 0.73	$55,034	$41,882	$7,971, $75,043
** **	** **	**300**	$14,303	$4,320, $23,150	0.32	0.12, 0.54	$44,788	$33,600	$10,835, $60,555
** **	** **	**1,000**	$12,370	$3,117, $21,030	0.26	0.09, 0.45	$48,000	$26,286	$8,688, $50,119
** **	** **	**3,000**	$11,191	$1,694, $19,903	0.21	0.07, 0.40	$52,996	$20,484	$6,703, $42,071
** **	**30**	**1**	$26,658	$15,870, $37,522	0.49	0.18, 0.80	$53,974	$47,426	$10,196, $83,300
** **	** **	**300**	$15,204	$4,422, $24,616	0.35	0.14, 0.59	$43,307	$37,458	$13,247, $67,934
** **	** **	**1,000**	$13,622	$3,387, $22,707	0.28	0.11, 0.50	$48,216	$28,755	$10,186, $56,132
** **	** **	**3,000**	$12,044	$1,620, $21,652	0.22	0.08, 0.44	$53,775	$21,551	$7,711, $46,938
**75–84**	**59**	**1**	$11,600	$6,826, $16,363	0.22	0.06, 0.36	$53,700	$20,802	$2,740, $38,344
** **	** **	**300**	$6,569	$2,318, $10,729	0.14	0.04, 0.26	$46,087	$14,811	$2,943, $28,320
** **	** **	**1,000**	$5,535	$1,641, $9,504	0.12	0.03, 0.22	$47,668	$11,883	$2,034, $23,988
** **	** **	**3,000**	$4,134	$1,069, $10,322	0.09	0.02, 0.25	$47,217	$8,998	$1,836, $27,385
** **	**45**	**1**	$13,631	$7,930, $19,345	0.26	0.08, 0.44	$51,628	$25,973	$4,125, $47,407
** **	** **	**300**	$8,598	$2,454, $14,478	0.20	0.05, 0.35	$43,901	$20,779	$5,158, $38,357
** **	** **	**1,000**	$7,363	$1,571, $13,217	0.16	0.04, 0.30	$45,964	$16,665	$3,967, $32,661
** **	** **	**3,000**	$5,554	$656, $14,688	0.12	0.03, 0.33	$46,166	$12,492	$3,154, $36,105
** **	**30**	**1**	$15,666	$8,975, $22,746	0.31	0.10, 0.53	$50,165	$31,177	$5,683, $56,641
** **	** **	**300**	$10,213	$2,303, $17,838	0.24	0.07, 0.42	$42,663	$25,694	$7,339, $46,803
** **	** **	**1,000**	$8,499	$842, $16,263	0.19	0.05, 0.36	$44,826	$19,942	$5,680, $39,474
** **	** **	**3,000**	$5,876	−$819, $16,413	0.13	0.03, 0.37	$45,799	$13,369	$3,541, $39,180

See S3 and S4 Tables for total costs and QALYs under MDC and usual care.

*eGFR units are ml/min/1.73 m^2^.

^†^UACR units are mg/g.

^‡^Net monetary benefit under a willingness to pay threshold of $150,000 per QALY gained.

eGFR, estimated glomerular filtration rate; ICER, incremental cost-effectiveness ratio; MDC, multidisciplinary care; QALY, quality-adjusted life year; UACR, urine albumin to creatinine ratio.

**Table 8:** Cost-effectiveness of MDC by sex.

Characteristic			Change in cost		Change in QALYs		ICER (cost/QALY)	Net monetary benefit^‡^	
Sex	eGFR*	UACR^†^	Estimate	95% CI	Estimate	95% CI		Estimate	95% CI
**Female**	**59**	**1**	$17,426	$10,844, $23,827	0.30	0.10, 0.49	$58,702	$27,103	$3,953, $49,146
** **	** **	**300**	$10,292	$4,286, $16,101	0.22	0.08, 0.37	$47,577	$22,156	$6,214, $40,585
** **	** **	**1,000**	$8,734	$3,152, $14,215	0.18	0.06, 0.31	$49,640	$17,657	$4,719, $33,921
** **	** **	**3,000**	$7,177	$2,148, $12,975	0.14	0.04, 0.28	$52,345	$13,389	$3,630, $30,190
** **	**45**	**1**	$19,602	$12,189, $26,888	0.34	0.12, 0.56	$56,955	$32,022	$5,425, $57,365
** **	** **	**300**	$11,907	$4,536, $18,947	0.27	0.10, 0.45	$44,888	$27,882	$8,734, $50,445
** **	** **	**1,000**	$10,383	$3,331, $17,124	0.22	0.08, 0.38	$47,518	$22,393	$6,901, $42,227
** **	** **	**3,000**	$8,981	$2,202, $16,202	0.18	0.06, 0.35	$50,653	$17,615	$5,554, $37,228
** **	**30**	**1**	$22,286	$13,872, $30,781	0.40	0.14, 0.65	$56,051	$37,355	$7,135, $65,949
** **	** **	**300**	$13,441	$4,875, $21,531	0.31	0.12, 0.53	$43,585	$32,818	$11,038, $59,320
** **	** **	**1,000**	$11,760	$3,558, $19,790	0.25	0.10, 0.45	$46,870	$25,875	$9,063, $49,980
** **	** **	**3,000**	$10,013	$1,999, $18,310	0.20	0.07, 0.40	$49,860	$20,110	$6,728, $43,252
**Male**	**59**	**1**	$17,411	$10,242, $24,964	0.30	0.10, 0.51	$57,265	$28,196	$4,680, $52,285
** **	** **	**300**	$9,644	$3,768, $15,211	0.19	0.06, 0.33	$50,791	$18,837	$4,633, $35,337
** **	** **	**1,000**	$7,815	$2,605, $13,139	0.15	0.04, 0.28	$52,352	$14,577	$3,346, $29,443
** **	** **	**3,000**	$5,985	$1,620, $14,152	0.11	0.03, 0.31	$52,242	$11,200	$2,678, $32,779
** **	**45**	**1**	$19,884	$11,504, $28,331	0.36	0.12, 0.60	$55,471	$33,886	$6,493, $61,616
** **	** **	**300**	$11,447	$4,000, $18,487	0.24	0.08, 0.41	$48,462	$23,985	$6,836, $43,825
** **	** **	**1,000**	$9,481	$2,676, $16,609	0.19	0.06, 0.35	$50,592	$18,629	$5,322, $37,120
** **	** **	**3,000**	$7,460	$1,350, $18,382	0.15	0.04, 0.39	$51,114	$14,431	$4,118, $40,516
** **	**30**	**1**	$22,871	$13,089, $32,639	0.42	0.15, 0.69	$54,548	$40,021	$8,640, $71,464
** **	** **	**300**	$12,996	$4,234, $21,489	0.27	0.10, 0.48	$47,505	$28,040	$8,809, $52,345
** **	** **	**1,000**	$10,594	$2,380, $19,206	0.21	0.07, 0.41	$50,220	$21,049	$6,619, $43,625
** **	** **	**3,000**	$7,602	$346, $19,937	0.15	0.04, 0.41	$51,692	$14,459	$4,669, $43,010

See S5 and S6 Tables for total costs and QALYs under MDC and usual care.

*eGFR units are ml/min/1.73 m^2^.

^†^UACR units are mg/g.

^‡^Net monetary benefit under a willingness to pay threshold of $150,000 per QALY gained.

eGFR, estimated glomerular filtration rate; ICER, incremental cost-effectiveness ratio; MDC, multidisciplinary care; QALY, quality-adjusted life year; UACR, urine albumin to creatinine ratio.

**Table 9:** Cost-effectiveness of MDC by race.

Characteristic			Change in cost		Change in QALYs		ICER (cost/QALY)	Net monetary benefit^‡^	
Race	eGFR*	UACR^†^	Estimate	95% CI	Estimate	95% CI		Estimate	95% CI
**White**	**59**	**1**	$16,722	$10,145, $23,124	0.29	0.10, 0.48	$57,218	$27,115	$4,229, $49,539
** **	** **	**300**	$9,680	$4,030, $14,945	0.20	0.07, 0.34	$48,250	$20,414	$5,506, $37,462
** **	** **	**1,000**	$8,103	$2,938, $12,939	0.16	0.05, 0.29	$49,759	$16,324	$4,113, $30,515
** **	** **	**3,000**	$6,446	$1,938, $12,360	0.12	0.04, 0.28	$51,617	$12,286	$3,202, $29,655
** **	**45**	**1**	$18,919	$11,457, $26,290	0.34	0.12, 0.56	$55,427	$32,282	$5,815, $57,944
** **	** **	**300**	$11,425	$4,333, $18,028	0.25	0.09, 0.42	$45,786	$26,004	$7,868, $46,905
** **	** **	**1,000**	$9,729	$3,158, $16,111	0.20	0.07, 0.36	$47,870	$20,756	$6,115, $38,716
** **	** **	**3,000**	$8,087	$1,907, $15,867	0.16	0.05, 0.35	$50,034	$16,157	$4,950, $37,315
** **	**30**	**1**	$21,468	$13,079, $30,045	0.39	0.14, 0.65	$54,406	$37,720	$7,665, $66,962
** **	** **	**300**	$13,038	$4,824, $20,764	0.29	0.11, 0.50	$44,487	$30,922	$10,207, $56,245
** **	** **	**1,000**	$11,058	$3,351, $18,634	0.23	0.09, 0.42	$47,194	$24,090	$8,109, $46,597
** **	** **	**3,000**	$8,842	$1,677, $17,462	0.18	0.06, 0.39	$49,267	$18,078	$6,112, $41,712
**Black**	**59**	**1**	$20,735	$12,267, $30,300	0.34	0.12, 0.58	$61,022	$30,234	$5,148, $57,323
** **	** **	**300**	$11,435	$4,258, $18,393	0.21	0.07, 0.37	$53,364	$20,708	$5,250, $38,555
** **	** **	**1,000**	$9,119	$3,024, $16,525	0.16	0.05, 0.33	$56,529	$15,078	$3,884, $34,148
** **	** **	**3,000**	$7,215	$1,749, $17,918	0.13	0.04, 0.36	$55,946	$12,129	$2,905, $37,311
** **	**45**	**1**	$23,758	$13,570, $34,052	0.40	0.14, 0.67	$59,288	$36,350	$6,996, $67,090
** **	** **	**300**	$13,105	$4,238, $21,773	0.26	0.08, 0.45	$50,924	$25,497	$7,269, $46,954
** **	** **	**1,000**	$10,930	$2,918, $20,335	0.20	0.06, 0.41	$54,516	$19,143	$5,659, $42,010
** **	** **	**3,000**	$8,830	$1,423, $22,664	0.16	0.04, 0.45	$54,927	$15,283	$4,269, $45,556
** **	**30**	**1**	$27,236	$15,352, $38,226	0.47	0.16, 0.76	$58,511	$42,586	$8,840, $76,300
** **	** **	**300**	$14,465	$4,182, $24,622	0.29	0.10, 0.52	$49,333	$29,517	$9,170, $54,790
** **	** **	**1,000**	$12,011	$2,308, $22,681	0.22	0.07, 0.46	$53,547	$21,635	$7,018, $46,870
** **	** **	**3,000**	$9,124	$164, $23,901	0.16	0.04, 0.47	$55,521	$15,527	$4,814, $48,189
**Other**	**59**	**1**	$18,130	$10,966, $25,383	0.31	0.10, 0.51	$58,571	$28,301	$4,480, $51,941
** **	** **	**300**	$9,860	$3,328, $16,417	0.20	0.06, 0.36	$48,886	$20,395	$5,310, $38,359
** **	** **	**1,000**	$8,184	$2,238, $15,086	0.16	0.05, 0.32	$51,995	$15,426	$3,946, $34,227
** **	** **	**3,000**	$6,501	$1,177, $15,916	0.13	0.03, 0.35	$51,574	$12,406	$3,140, $36,033
** **	**45**	**1**	$20,387	$12,156, $28,556	0.36	0.12, 0.59	$56,765	$33,485	$6,201, $60,435
** **	** **	**300**	$11,235	$3,041, $19,370	0.24	0.08, 0.43	$46,075	$25,341	$7,420, $46,577
** **	** **	**1,000**	$9,876	$1,856, $18,566	0.20	0.06, 0.39	$49,514	$20,044	$5,863, $41,917
** **	** **	**3,000**	$8,124	$676, $20,386	0.16	0.04, 0.43	$51,536	$15,522	$4,668, $43,931
** **	**30**	**1**	$22,328	$13,222, $31,339	0.40	0.14, 0.66	$55,376	$38,153	$7,729, $68,190
** **	** **	**300**	$12,087	$2,439, $21,438	0.27	0.09, 0.48	$44,080	$29,044	$9,403, $53,690
** **	** **	**1,000**	$10,684	$952, $20,657	0.22	0.07, 0.44	$47,544	$23,024	$7,116, $46,827
** **	** **	**3,000**	$8,427	−$840, $22,023	0.16	0.04, 0.45	$53,641	$15,137	$5,174, $46,628

See S7 and S8 Tables for total costs and QALYs under MDC and usual care.

*eGFR units are ml/min/1.73 m^2^.

^†^UACR units are mg/g.

^‡^Net monetary benefit under a willingness to pay threshold of $150,000 per QALY gained.

eGFR, estimated glomerular filtration rate; ICER, incremental cost-effectiveness ratio; MDC, multidisciplinary care; QALY, quality-adjusted life year; UACR, urine albumin to creatinine ratio.

**Table 10:** Hazard ratios of multidisciplinary care by severity of kidney disease.

Characteristic		Hazard ratio for death		Hazard ratio for ESRD	
eGFR*	UACR^†^	Estimate	95% CI	Estimate	95% CI
**59**	**1**	0.70	0.56, 0.89	0.55	0.36, 0.85
** **	**300**	0.78	0.65, 0.92	0.60	0.41, 0.86
** **	**1,000**	0.82	0.69, 0.95	0.66	0.48, 0.92
** **	**3,000**	0.87	0.69, 0.97	0.74	0.52, 0.93
**45**	**1**	0.69	0.54, 0.88	0.55	0.36, 0.84
** **	**300**	0.75	0.62, 0.91	0.59	0.41, 0.85
** **	**1,000**	0.79	0.66, 0.93	0.64	0.45, 0.87
** **	**3,000**	0.84	0.66, 0.96	0.69	0.48, 0.89
**30**	**1**	0.67	0.52, 0.87	0.56	0.37, 0.85
** **	**300**	0.73	0.60, 0.90	0.59	0.40, 0.83
** **	**1,000**	0.78	0.64, 0.92	0.61	0.42, 0.83
** **	**3,000**	0.83	0.64, 0.95	0.64	0.43, 0.84
**Overall**		0.77	0.63, 0.92	0.62	0.43, 0.86

*eGFR units are ml/min/1.73 m^2^.

^†^UACR units are mg/g.

eGFR, estimated glomerular filtration rate; ESRD, end-stage renal disease; UACR, urine albumin to creatinine ratio.

**Table 11:** Hazard ratios of multidisciplinary care by age.

Characteristic			Hazard ratio for death		Hazard ratio for ESRD	
Age (years)	eGFR*	UACR^†^	Estimate	95% CI	Estimate	95% CI
**45–64**	**59**	**1**	0.66	0.51, 0.85	0.58	0.38, 0.87
** **	** **	**300**	0.79	0.67, 0.90	0.67	0.48, 0.88
** **	** **	**1,000**	0.84	0.73, 0.93	0.71	0.54, 0.90
** **	** **	**3,000**	0.87	0.77, 0.94	0.76	0.57, 0.92
** **	**45**	**1**	0.65	0.51, 0.84	0.58	0.38, 0.87
** **	** **	**300**	0.80	0.67, 0.90	0.66	0.47, 0.87
** **	** **	**1,000**	0.85	0.74, 0.93	0.70	0.51, 0.88
** **	** **	**3,000**	0.87	0.78, 0.94	0.71	0.53, 0.89
** **	**30**	**1**	0.65	0.51, 0.84	0.58	0.38, 0.87
** **	** **	**300**	0.80	0.68, 0.91	0.66	0.47, 0.86
** **	** **	**1,000**	0.86	0.74, 0.93	0.69	0.50, 0.87
** **	** **	**3,000**	0.87	0.78, 0.94	0.68	0.50, 0.88
**65–74**	**59**	**1**	0.68	0.53, 0.88	0.56	0.37, 0.85
** **	** **	**300**	0.75	0.62, 0.90	0.61	0.42, 0.86
** **	** **	**1,000**	0.80	0.68, 0.93	0.66	0.48, 0.89
** **	** **	**3,000**	0.85	0.71, 0.96	0.75	0.54, 0.93
** **	**45**	**1**	0.67	0.52, 0.87	0.56	0.37, 0.86
** **	** **	**300**	0.74	0.61, 0.89	0.62	0.43, 0.86
** **	** **	**1,000**	0.80	0.67, 0.92	0.66	0.47, 0.87
** **	** **	**3,000**	0.84	0.71, 0.95	0.71	0.51, 0.89
** **	**30**	**1**	0.66	0.51, 0.86	0.57	0.37, 0.86
** **	** **	**300**	0.74	0.61, 0.89	0.63	0.44, 0.86
** **	** **	**1,000**	0.80	0.67, 0.91	0.65	0.46, 0.86
** **	** **	**3,000**	0.84	0.71, 0.94	0.67	0.48, 0.86
**75–84**	**59**	**1**	0.72	0.58, 0.90	0.54	0.36, 0.84
** **	** **	**300**	0.79	0.66, 0.94	0.58	0.39, 0.87
** **	** **	**1,000**	0.82	0.70, 0.96	0.65	0.46, 0.96
** **	** **	**3,000**	0.87	0.67, 0.97	0.74	0.50, 0.94
** **	**45**	**1**	0.70	0.55, 0.89	0.55	0.36, 0.84
** **	** **	**300**	0.75	0.62, 0.92	0.57	0.38, 0.84
** **	** **	**1,000**	0.78	0.65, 0.95	0.62	0.43, 0.87
** **	** **	**3,000**	0.83	0.62, 0.96	0.68	0.46, 0.88
** **	**30**	**1**	0.68	0.53, 0.88	0.55	0.36, 0.84
** **	** **	**300**	0.72	0.57, 0.90	0.56	0.38, 0.82
** **	** **	**1,000**	0.76	0.60, 0.93	0.57	0.39, 0.81
** **	** **	**3,000**	0.82	0.59, 0.97	0.62	0.38, 0.84

*eGFR units are ml/min/1.73 m^2^.

^†^UACR units are mg/g.

eGFR, estimated glomerular filtration rate; ESRD, end-stage renal disease; UACR, urine albumin to creatinine ratio.

**Table 12:** Hazard ratios of multidisciplinary care by sex.

Characteristic			Hazard ratio for death		Hazard ratio for ESRD	
Sex	eGFR*	UACR^†^	Estimate	95% CI	Estimate	95% CI
**Female**	**59**	**1**	0.70	0.57, 0.89	0.56	0.37, 0.85
** **	** **	**300**	0.76	0.63, 0.91	0.59	0.40, 0.85
** **	** **	**1,000**	0.81	0.68, 0.94	0.65	0.45, 0.92
** **	** **	**3,000**	0.86	0.70, 0.96	0.75	0.52, 0.94
** **	**45**	**1**	0.69	0.55, 0.88	0.56	0.37, 0.85
** **	** **	**300**	0.74	0.61, 0.90	0.59	0.40, 0.84
** **	** **	**1,000**	0.78	0.65, 0.93	0.63	0.44, 0.87
** **	** **	**3,000**	0.83	0.68, 0.95	0.69	0.48, 0.88
** **	**30**	**1**	0.68	0.53, 0.87	0.56	0.37, 0.85
** **	** **	**300**	0.73	0.59, 0.89	0.59	0.40, 0.83
** **	** **	**1,000**	0.77	0.63, 0.91	0.61	0.43, 0.84
** **	** **	**3,000**	0.81	0.66, 0.94	0.63	0.44, 0.85
**Male**	**59**	**1**	0.70	0.56, 0.89	0.55	0.36, 0.84
** **	** **	**300**	0.79	0.66, 0.94	0.61	0.42, 0.90
** **	** **	**1,000**	0.83	0.70, 0.96	0.67	0.49, 0.94
** **	** **	**3,000**	0.88	0.68, 0.97	0.74	0.52, 0.93
** **	**45**	**1**	0.69	0.54, 0.88	0.55	0.36, 0.84
** **	** **	**300**	0.76	0.63, 0.92	0.60	0.41, 0.85
** **	** **	**1,000**	0.80	0.67, 0.95	0.64	0.46, 0.88
** **	** **	**3,000**	0.85	0.63, 0.96	0.69	0.48, 0.89
** **	**30**	**1**	0.67	0.52, 0.87	0.55	0.36, 0.84
** **	** **	**300**	0.74	0.60, 0.91	0.59	0.40, 0.83
** **	** **	**1,000**	0.79	0.64, 0.94	0.60	0.42, 0.83
** **	** **	**3,000**	0.85	0.62, 0.97	0.66	0.40, 0.85

*eGFR units are ml/min/1.73 m^2^.

^†^UACR units are mg/g.

eGFR, estimated glomerular filtration rate; ESRD, end-stage renal disease; UACR, urine albumin to creatinine ratio.

**Table 13:** Hazard ratios of multidisciplinary care by race.

Characteristic			Hazard ratio for death		Hazard ratio for ESRD	
Race	eGFR*	UACR^†^	Estimate	95% CI	Estimate	95% CI
**White**	**59**	**1**	0.70	0.57, 0.89	0.55	0.36, 0.85
** **	** **	**300**	0.77	0.64, 0.92	0.60	0.41, 0.87
** **	** **	**1,000**	0.81	0.69, 0.95	0.64	0.47, 0.93
** **	** **	**3,000**	0.86	0.69, 0.96	0.75	0.52, 0.94
** **	**45**	**1**	0.69	0.54, 0.88	0.55	0.36, 0.84
** **	** **	**300**	0.74	0.61, 0.90	0.59	0.41, 0.84
** **	** **	**1,000**	0.78	0.66, 0.93	0.63	0.45, 0.87
** **	** **	**3,000**	0.83	0.66, 0.95	0.69	0.48, 0.89
** **	**30**	**1**	0.67	0.52, 0.87	0.56	0.37, 0.85
** **	** **	**300**	0.72	0.58, 0.89	0.59	0.41, 0.83
** **	** **	**1,000**	0.76	0.63, 0.91	0.60	0.42, 0.83
** **	** **	**3,000**	0.82	0.64, 0.95	0.63	0.43, 0.84
**Black**	**59**	**1**	0.70	0.56, 0.89	0.55	0.37, 0.85
** **	** **	**300**	0.80	0.68, 0.94	0.61	0.42, 0.87
** **	** **	**1,000**	0.86	0.70, 0.96	0.72	0.50, 0.94
** **	** **	**3,000**	0.88	0.68, 0.98	0.74	0.52, 0.94
** **	**45**	**1**	0.69	0.54, 0.88	0.55	0.37, 0.85
** **	** **	**300**	0.78	0.65, 0.93	0.60	0.41, 0.85
** **	** **	**1,000**	0.83	0.67, 0.95	0.67	0.46, 0.88
** **	** **	**3,000**	0.86	0.64, 0.97	0.70	0.48, 0.89
** **	**30**	**1**	0.67	0.52, 0.87	0.56	0.37, 0.85
** **	** **	**300**	0.77	0.63, 0.92	0.59	0.40, 0.83
** **	** **	**1,000**	0.82	0.66, 0.95	0.63	0.42, 0.84
** **	** **	**3,000**	0.87	0.63, 0.98	0.67	0.41, 0.86
**Other**	**59**	**1**	0.70	0.56, 0.89	0.55	0.37, 0.85
** **	** **	**300**	0.80	0.68, 0.95	0.60	0.41, 0.88
** **	** **	**1,000**	0.86	0.70, 0.97	0.72	0.49, 0.93
** **	** **	**3,000**	0.88	0.69, 0.98	0.74	0.53, 0.94
** **	**45**	**1**	0.69	0.54, 0.88	0.56	0.37, 0.85
** **	** **	**300**	0.79	0.66, 0.94	0.60	0.41, 0.86
** **	** **	**1,000**	0.83	0.69, 0.96	0.66	0.46, 0.88
** **	** **	**3,000**	0.87	0.66, 0.98	0.71	0.49, 0.90
** **	**30**	**1**	0.68	0.53, 0.88	0.56	0.37, 0.86
** **	** **	**300**	0.78	0.64, 0.93	0.59	0.40, 0.84
** **	** **	**1,000**	0.82	0.68, 0.96	0.60	0.42, 0.85
** **	** **	**3,000**	0.87	0.66, 0.98	0.70	0.43, 0.87

*eGFR units are ml/min/1.73 m^2^.

^†^UACR units are mg/g.

eGFR, estimated glomerular filtration rate; ESRD, end-stage renal disease; UACR, urine albumin to creatinine ratio.

On average, MDC cost $12,001 (95% CI: $5,098, $19,358) more than usual care per patient over the lifetime, an increase from $68,571 to $80,572 (Tables 6–9). The corresponding ICER was $51,285 per QALY gained, which was cost-effective at a threshold of $150,000 per QALY (net monetary benefit of $23,100; 95% CI: $6,252, $44,323). The ICERs ranged from $42,663 to $72,432 per QALY gained in all subgroups. In general, MDC was more expensive in patients with lower levels of albuminuria. For instance, patients with a UACR of 1 mg/g had ICERs of $55,315 to $57,958 per QALY gained versus $48,323 to $50,916 per QALY gained in patients with a UACR of 1,000 mg/g. Similarly, younger patients tended to have higher ICERs, though these higher relative expenses brought forth greater improvements in health.

### Effectiveness sensitivity analyses

MDC remained cost-effective at the WTP threshold of $150,000 per QALY in most sensitivity analyses where we varied its effectiveness (Tables 14 and S9–S11). Even when the program was only 25% as effective as the base case, MDC was cost-effective on average in the entire population, with an ICER of $127,927 per QALY gained, though the 95% confidence interval for net monetary benefit did cross $0 (net monetary benefit of $1,076; 95% CI: −$2,194, $4,088). This corresponded to hazard ratios of 0.95 for death and 0.91 for progression to ESRD. Notably, MDC was not cost-effective when operating at 25% effectiveness in patients with an eGFR of 59 ml/min/1.73 m^2^ and UACR of 1 mg/g (ICER $151,869 per QALY) (S9 Table). When we assumed that MDC did not attenuate progression to ESRD and was only 25% as effective as the base case in preventing death (hazard ratios of 0.93–0.97 for death and 1.00–1.02 for progression to ESRD), the program was not cost-effective in all subgroups.

**Table 14:** Cost-effectiveness when varying the effectiveness of MDC—whole population.

Scenario	HR for death		HR for ESRD		ICER (cost/QALY)	Net monetary benefit*	
	Estimate	95% CI	Estimate	95% CI		Estimate	95% CI
Base case	0.77	0.63, 0.92	0.62	0.43, 0.86	$51,285	$23,100	$6,252, $44,323
50% of base case	0.89	0.83, 0.96	0.82	0.75, 0.94	$77,870	$7,425	$405, $14,311
25% of base case	0.95	0.92, 0.98	0.91	0.88, 0.97	$127,927	$1,076	−$2,194, $4,088
100% of non-discounted	0.74	0.57, 0.91	0.60	0.41, 0.85	$48,270	$29,083	$8,109, $56,131
50% of non-discounted	0.87	0.80, 0.96	0.81	0.73, 0.93	$69,953	$10,112	$1,322, $18,958
25% of non-discounted	0.94	0.90, 0.98	0.90	0.87, 0.96	$110,660	$2,356	−$1,747, $6,222
Only mortality, base case	0.82	0.69, 0.95	1.04	1.01, 1.10	$76,420	$12,957	−$156, $28,180
Only mortality, 50% of base case	0.91	0.85, 0.97	1.02	1.01, 1.04	$106,096	$3,603	−$2,459, $9,882
Only mortality, 25% of base case	0.95	0.92, 0.99	1.01	1.00, 1.02	$165,579	−$618	−$3,570, $2,243

We summarize each of the scenarios below. Base case: MDC effectiveness was 25% in stage 3 CKD, 50% in stage 4 CKD, 100% in stage 5 CKD. 50% of base case/25% of base case: MDC effectiveness was 12.5%/6.25% in stage 3 CKD, 25%/12.5% in stage 4 CKD, 50%/25% in stage 5 CKD, respectively. 100%, 50%, and 25% of non-discounted: MDC effectiveness was 100%, 50%, and 25% in all CKD stages, respectively. Only mortality: Same as above except MDC was effective in reducing only mortality (not progression to ESRD). See S9–S11 Tables for detailed cost-effectiveness estimates by eGFR and albuminuria levels.

*Net monetary benefit under a willingness to pay threshold of $150,000 per QALY gained.

ESRD, end-stage renal disease; HR, hazard ratio; ICER, incremental cost-effectiveness ratio; MDC, multidisciplinary care; QALY, quality-adjusted life year.

### Cost sensitivity analyses

**Fig 2:**
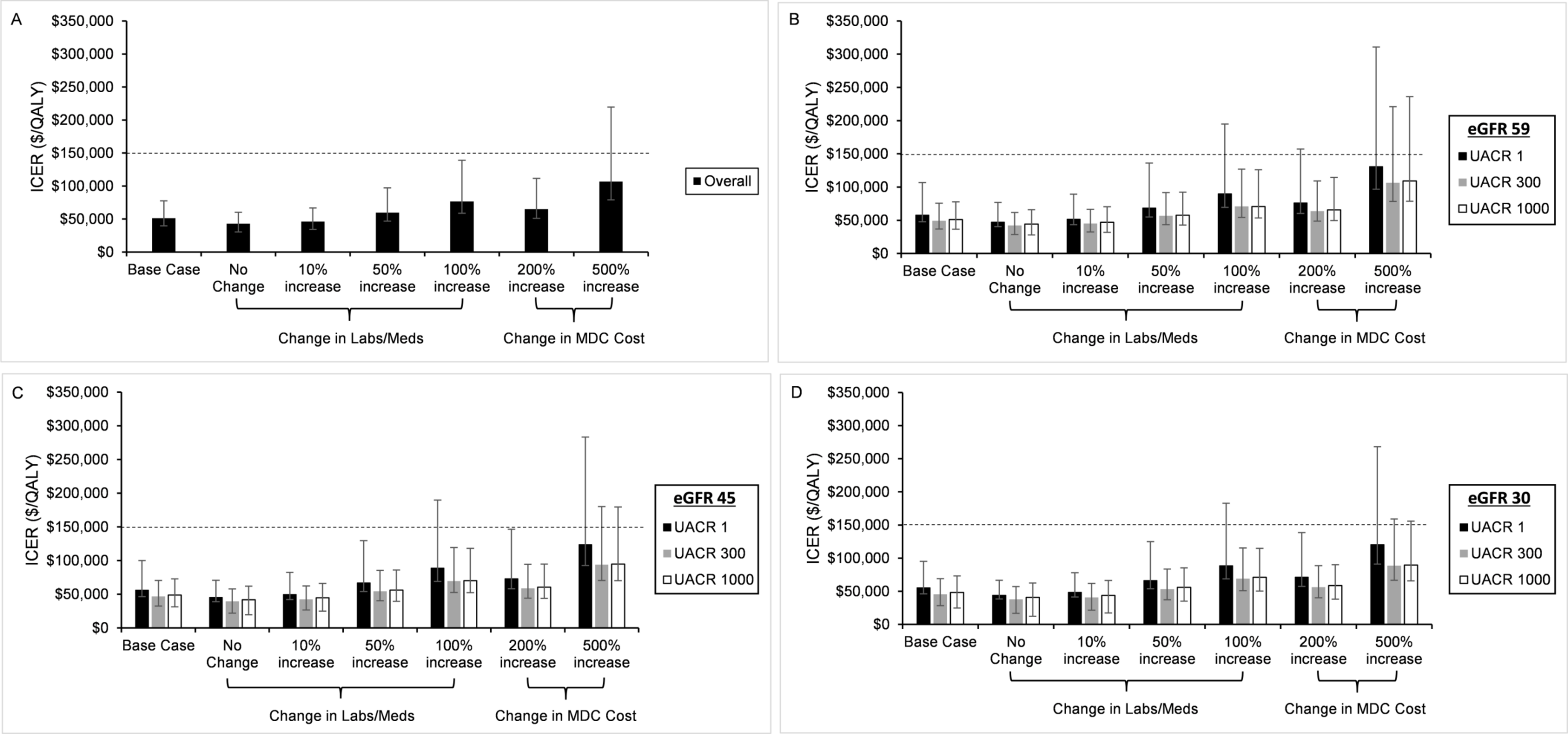
Varying the cost of multidisciplinary care (MDC). In sensitivity analyses, we varied the cost of MDC. We depict the incremental cost-effectiveness ratios (ICERs) for the entire population (A) and stratified by estimated glomerular filtration rate (eGFR) and urine albumin to creatinine ratio (UACR) (B–D). Our base case assumed that MDC increased the use of chronic kidney disease–specific medications (Meds) and laboratory tests (Labs) in 25% of the population. In subsequent analyses, we assumed that MDC increased the use of these medications and laboratory tests in 0%, 10%, 50%, and 100% of the population. We also increased the cost of the entire program 2-fold and 5-fold. In all analyses, we found that MDC remained cost-effective, but the upper end of the 95% confidence intervals exceeded the willingness to pay threshold of $150,000 per QALY in the most expensive cases. The program was more expensive (higher ICERs) in patients with UACR of 1 mg/g when compared to patients with UACR of 300 or 1,000 mg/g.

In all scenarios that varied the cost, we found that point estimates for the ICERs were less than $150,000 per QALY gained (Fig 2). MDC remained cost-effective even when we increased the monthly cost 5-fold, although the upper end of the 95% confidence interval exceeded our threshold for cost-effectiveness. Additionally, MDC remained cost-effective when it increased the use of medications and laboratory tests in 100% of the population. In this case, the upper end of the 95% confidence interval for patients without albuminuria exceeded $150,000 per QALY gained. For patients with higher levels of albuminuria, the 95% confidence interval remained less than $150,000 per QALY gained. In all cost sensitivity analyses, we found that MDC was more expensive in patients without albuminuria versus those with UACR of 300 or 1,000 mg/g.

### Probabilistic sensitivity analysis

**Fig 3:**
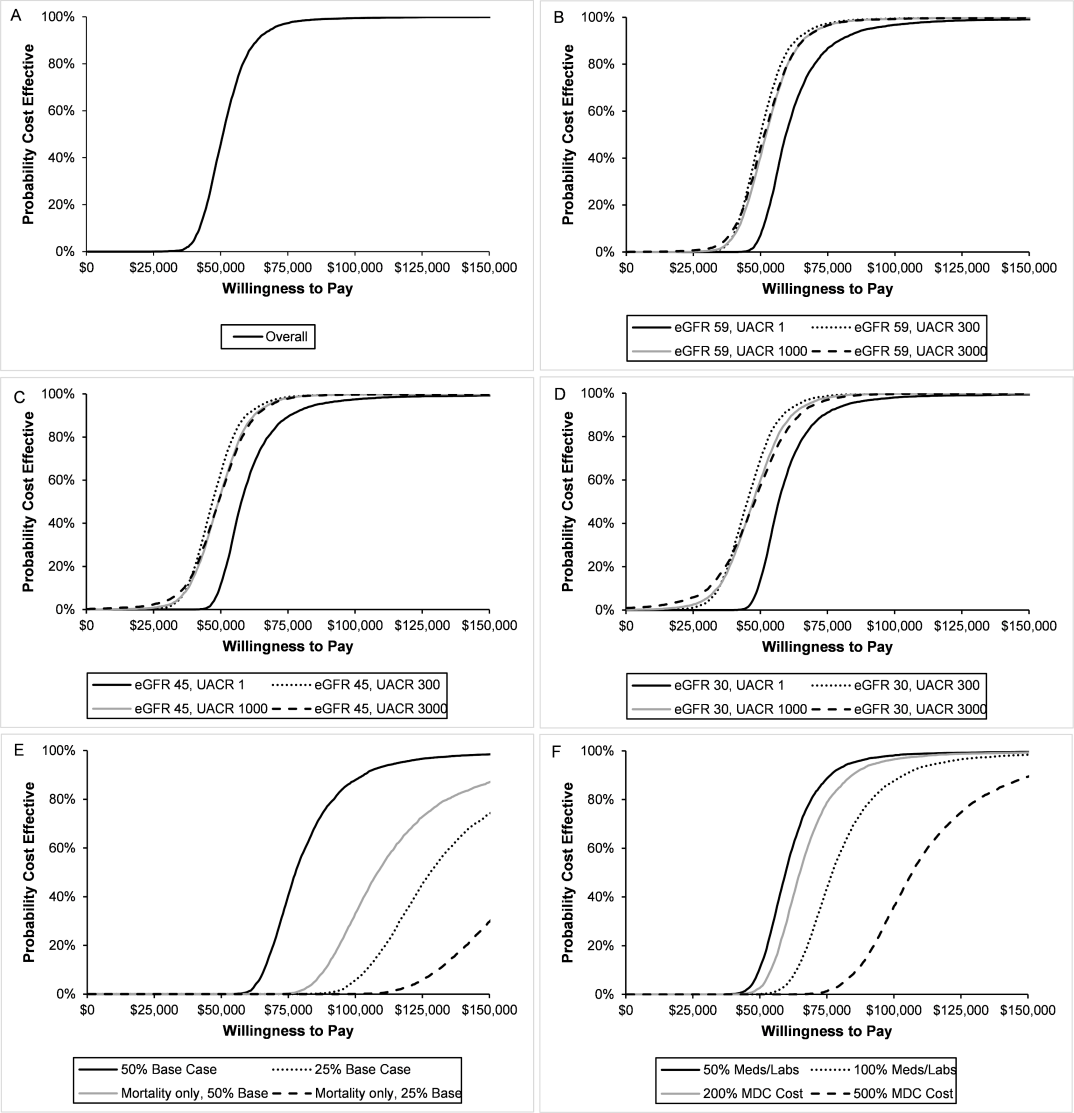
Cost-effectiveness acceptability curves for multidisciplinary care (MDC). We estimated the probability that the MDC program was cost-effective in different populations and with all sensitivity analyses. We show the acceptability curves for the overall population (A) and stratified by estimated glomerular filtration rate (eGFR) and urine albumin to creatinine ratio (UACR) (B–D). In all populations, greater than 95% of probability sensitivity analyses costed less than $90,000 per QALY, and greater than 99% of analyses costed less than $150,000 per QALY. In general, MDC was less likely to be cost-effective in populations with no albuminuria (UACR 1 mg/g) and higher eGFR (59 ml/min/1.73 m^2^). We also assessed the probability when varying effectiveness (E) and cost (F). When MDC was 50% as effective as the base case, it was cost-effective with 98% probability at a willingness to pay (WTP) threshold of $150,000 per QALY. This probability dropped with other effectiveness scenarios. For most cost scenarios, MDC was cost-effective with 95% probability at a WTP of $150,000, though this probability dropped to 89% when MDC cost 5 times the base case.

In the base case, we found that MDC was cost-effective in over 99% of probabilistic sensitivity analyses at a threshold of $87,500 per QALY and in over 95% of probabilistic sensitivity analyses at a threshold of $70,000 per QALY (Fig 3A–3D). In all subgroups, we found that MDC cost less than $150,000 per QALY in over 99% of probabilistic sensitivity analyses. Although findings were similar across eGFR levels, MDC tended to have a lower probability of cost-effectiveness for patients with less albuminuria.

When we decreased the effectiveness of MDC to 50% of the base case, we found that the program was cost-effective with 98% probability at a threshold of $150,000 per QALY (Fig 3E). However, the probability dropped to 74% under the scenario where the program was 25% as effective as the base case. The probability of cost-effectiveness dropped further when we assumed MDC only affected mortality and not progression to ESRD.

In most cost sensitivity analyses, the probability that MDC was cost-effective was greater than 95% at a threshold of $150,000 per QALY gained (Fig 3F). However, once we increased the cost of MDC 5-fold (relative to the base case), the probability declined to 89%.

## Discussion

Using data from literature, we developed and calibrated a deterministic Markov model that accurately models disease progression of patients with stage 3 and 4 CKD and accounts for population heterogeneity including age, sex, race, severity of kidney disease, and albuminuria. From this model, we found that a Medicare-funded MDC program in non-dialysis-requiring (eGFR 20 to 59 ml/min/1.73 m^2^) CKD is cost-effective in middle-aged to elderly patients. Although cost-effective in all subgroups, the program is more cost-effective in patients with more advanced CKD, particularly those with higher levels of albuminuria. Provision of MDC to younger patients was more expensive, but younger patients gained the most in health outcomes, as measured by QALYs. Notably, MDC remained cost-effective even if it was 5 times more expensive or one-quarter as effective as our base case.

Treating CKD requires effective management across multiple dimensions. Blood pressure control, especially the use of ACE inhibitors and angiotensin receptor blockers, is a mainstay of care [57,58]. Reducing cardiovascular risk factors and managing diet, fluid, and electrolyte balance are also important adjuncts [59,60]. Medication and dietary non-adherence are prevalent and can limit the success of CKD treatment [61,62].

MDC in CKD could help bridge the gap between successful scientific advances and lagging population health. Since CKD is a heterogeneous disease, determining subgroups that benefit most is important. Younger patients and those with less albuminuria gained the most QALYs. These findings are likely due to longer baseline life expectancy, since we found similar relative gains in health in all subpopulations. This probably explains why MDC is more expensive in younger patients and patients with less albuminuria, since a longer lifespan leads to additional healthcare spending, including on MDC. Importantly, we found that MDC represents excellent value potential for patients with higher levels of albuminuria, where slowing kidney progression can delay the onset of dialysis. Our study could help providers and program developers identify the subgroups of patients most likely to benefit from MDC.

Surprisingly, MDC was cost-effective, albeit more expensive, in patients without albuminuria, suggesting that MDC may be worthwhile in this population. The added expense is likely due to poor generalizability of MDC to patients without albuminuria who are at low risk for developing ESRD [33]. Even though MDC was cost-effective in this group on average, many such patients probably would not benefit from an intensive MDC program, especially one that aims to slow progression to ESRD. In patients without albuminuria, benefits from MDC probably reflect reduced mortality from cardiovascular disease rather than slowed CKD progression [63]. This finding was corroborated by our sensitivity analysis in which we assumed that MDC did not attenuate progression to ESRD. Here, MDC remained cost-effective, except under the most pessimistic scenario. Future studies of MDC could focus on disentangling the components of MDC that reduce cardiovascular complications and mortality from those that slow progression to ESRD, which would increase its applicability to patients without albuminuria.

Few studies have attempted to assess the economic impact of MDC in CKD, and none to our knowledge has used a CKD progression model incorporating disease heterogeneity. Several simulation studies have suggested that more optimally managing CKD could lead to up to $20,000 of savings per patient (€17,700 per patient) [22] and $60 billion annually [13]. Because these studies aimed to quantify only the savings associated with slowing progression to ESRD, they did not consider the cost of the intervention used to improve CKD management. We found that the cost of an MDC program would likely exceed potential savings from preventing the onset of ESRD, but that these excess costs would be relatively modest. Other studies using data from MDC trials also reported that MDC could lead to cost savings [10,17,41]. However, these studies did not incorporate the cost of anemia and bone-mineral metabolism management, which can be expensive, and none accrued costs over patients’ lifetimes.

Our results indicate that a US Medicare-funded MDC program would likely have significant costs. Thus, it is unlikely that healthcare providers would independently deploy such a program without reimbursement, since MDC could cost a provider seeing 100 patients with CKD $800,000 to $2,000,000 annually, assuming Medicare reimburses at marginal cost. This could explain why MDC has not had widespread adoption, especially as Medicare has begun rewarding providers for demonstrating cost savings [64–66]. While we did not show a reduction in Medicare expenditures, we found that modest investment in CKD care management could yield substantial gains in health. Additionally, our findings in patients 45–64 years of age could be extended to non-Medicare US healthcare payers because the vast majority of patients do not qualify for Medicare until they turn 65. Beneficiaries of these healthcare payers potentially have the most to gain, since MDC is especially beneficial for younger patients.

Other countries could also see improvements in health with small investments in MDC programs. The global prevalence of CKD is reported to be 8%–16% worldwide and continues to grow [67–69]. MDC in CKD has been successfully tested in non-US populations, including in Canada [7,15,16,42,46,70], the United Kingdom [43], France [44], Italy [70], the Netherlands [11], South Korea [14], and Taiwan [17,19,41,45,47,48], and could reduce the growing need for renal replacement at reasonable cost. In developing countries with poor access to renal replacement, MDC could be an inexpensive alternative to providing dialysis, which requires additional investment in infrastructure and capital [71,72].

Although Wang et al. reported that MDC reduces mortality and ESRD in CKD [21], we believe that their effectiveness estimates were inflated. Many of the included studies were not randomized controlled trials, and probably reflected populations likely to benefit. Additionally, providers actively investigating MDC are probably among the best at care management. Implementing a national program may be less efficient, especially if adherence to the program is modest or poor. It seems unlikely that a large-scale MDC program would be as effective as our base case. However, when we tested programs with much smaller effect sizes, MDC remained cost-effective, particularly in more severe kidney disease. Testing MDC programs that were less effective than our base case also allowed us to capture scenarios where patient adherence is poor. Nevertheless, Medicare would probably benefit from pursuing a cautious approach. Our most pessimistic scenario was not cost-effective, especially in patients with mild to moderate, non-proteinuric CKD. Implementing a pilot program for patients vulnerable to progressing to ESRD could increase the certainty that a nationally implemented program would be cost-effective.

Our study had some important limitations. First, the literature likely overestimates the effectiveness of MDC. We attempted to address this issue by using conservative estimates for our base case and by extensively testing changes in cost and effectiveness. We found that substantially less effective programs remained cost-effective. Second, our simulations relied on a calibrated model based on reported CKD progression and mortality rates. Results from published literature have limited generalizability, and although we accounted for population-level heterogeneity, our model cannot fully incorporate differences in patient factors. Third, although our model was novel in that it accounted for aspects of population-level heterogeneity, including age, sex, race, and severity of kidney disease, our model was unable to capture other known determinants of CKD progression such as socioeconomic status [73–75]. Fourth, our analysis relied on prior studies that did not generally stratify effectiveness by CKD stage. We accounted for this by assuming that MDC was less effective in milder stages of CKD and by varying its effectiveness in different CKD stages. Finally, we limited our investigation to patients with mild to moderate CKD and only studied the effect of MDC on CKD progression and all-cause mortality. Given the absence of data on the effect of MDC on intermediate endpoints, we were unable to incorporate cardiovascular or other hospitalizations into our cost-effectiveness estimates.

Strengths of our study include our development and calibration of a novel CKD progression model, which was able to reliably reproduce long-term rates of mortality and progression to ESRD in many different subpopulations. Using this model, we were able to detect variation in the cost-effectiveness of MDC that incorporates heterogeneity in the population. Our model is also flexible enough to allow investigators to test other interventions in patients with CKD. Additionally, we tested a wide swath of effectiveness and cost scenarios, and our results were robust to these changes except in the most pessimistic scenarios. Finally, our probabilistic sensitivity analysis allowed us to simultaneously test variation in all our parameters while accounting for correlations. By fitting our parameters to probability distributions, we were able to ensure that the joint distribution of our model fit parameters reported in literature.

In conclusion, our model estimates suggest that a Medicare-funded MDC program, even if implemented with modest efficiency, is likely to be cost-effective in middle-aged to elderly patients with mild to moderate CKD. Reimbursing providers for intensive disease management could be a relatively inexpensive way to improve the health of patients with CKD.

## Supporting information

S1 Appendix Technical specifications. (DOCX)

S2 Appendix Calibration results. (DOCX)

S1 Table Costs under multidisciplinary care and usual care, by severity of kidney disease. (DOCX)

S2 Table Quality-adjusted life years under multidisciplinary care and usual care, by severity of kidney disease. (DOCX)

S3 Table Costs under multidisciplinary care and usual care, by age. (DOCX)

S4 Table Quality-adjusted life years under multidisciplinary care and usual care, by age. (DOCX)

S5 Table Costs under multidisciplinary care and usual care, by sex. (DOCX)

S6 Table Quality-adjusted life years under multidisciplinary care and usual care, by sex. (DOCX)

S7 Table Costs under multidisciplinary care and usual care, by race. (DOCX)

S8 Table Quality-adjusted life years under multidisciplinary care and usual care, by race. (DOCX)

S9 Table Cost-effectiveness when varying the effectiveness of multidisciplinary care in patients with eGFR of 59 ml/min/1.73 m^2^. (DOCX)

S10 Table Cost-effectiveness when varying the effectiveness of multidisciplinary care in patients with eGFR of 45 ml/min/1.73 m^2^. (DOCX)

S11 Table Cost-effectiveness when varying the effectiveness of multidisciplinary care in patients with eGFR of 30 ml/min/1.73 m^2^. (DOCX)

S1 Text Consolidated Health Economic Evaluation Reporting Standards (CHEERS) checklist. We report section and paragraph locations for each item recommended by the International Society for Pharmacoeconomics and Outcomes for cost-effectiveness analyses.
(DOC)

## Abbreviations

CDC: Centers for Disease Control and Prevention
CKD: chronic kidney disease
CPT: Current Procedural Terminology
eGFR: estimated glomerular filtration rate
ESRD: end-stage renal disease
ICER: incremental cost-effectiveness ratio
MDC: multidisciplinary care
QALY: quality-adjusted life year
UACR: urine albumin to creatinine ratio
USRDS: United States Renal Data System
WTP: willingness to pay
