# Supplementary material for: Cost-effectiveness of multidisciplinary care in mild to moderate chronic kidney disease in the United States: A modeling study
Source: PLoS Med. 2018 Mar 27;15(3):e1002532. doi: 10.1371/journal.pmed.1002532 (PMC5870947; doi:10.1371/journal.pmed.1002532)
Supplement: S1 Appendix — (DOCX) [file pmed.1002532.s001.docx]

# S1 APPENDIX: TECHNICAL SPECIFICATIONS

## Analysis 1: Chronic Kidney Disease Progression Model, Construction and Calibration

### CKD Progression Model

To simulate accrued costs and quality-adjusted life years (QALYs) over time, we developed a deterministic Markov model of chronic kidney disease (CKD) progression for different subpopulations: US patients of different ages (45-64, 65-74, and 75-84 years old), sexes (female and male), races (white, black, and other), estimated glomerular filtration rates (eGFRs from 20-59 cc/min/1.73 m^2^), and approximate albuminuria levels (1, 300, 1000, and 3000 mg/g). We simulated CKD progression by modeling estimated glomerular filtration rate (eGFR) decrements of 5 mL/min/1.73 m^2^ as individual health states from 59 to 5 mL/min/1.73 m^2^. We used monthly cycles, where patients can either decline to the next eGFR level, die, or remain at the same eGFR. We did not accommodate improvements in eGFR and also did not allow simulated patients to skip eGFR levels. From 15 to 5 mL/min/1.73 m^2^, patients could also progress to end-stage renal disease (ESRD).

Our study focused on multi-disciplinary care (MDC) programs that aim to slow progression to ESRD in patients with CKD stages 3 and 4. MDC programs that focus on CKD stages 3 and 4 would not address dialysis planning, since the majority of patients in these stages do not progress to ESRD. We therefore assumed that our MDC program of interest would not change the rate of patients receiving dialysis through a tunneled dialysis catheter or through peritoneal dialysis. For this reason, we modeled ESRD as a single health state that encompasses hemodialysis with a tunneled dialysis catheter, hemodialysis with an arteriovenous fistula or graft, peritoneal dialysis, and kidney transplant. In this Appendix, we call the CKD progression model, which considers ESRD as a single health state, the “final model” (**Fig 1**).

However, in developing and calibrating our model, we used additional health states to model the average ESRD health state and to allow the flexibility to test other interventions in other studies, such as MDC programs geared towards improving the transition from late stage CKD and ESRD. This model, which we call the “developmental model,” split the ESRD health state into two separate health states, hemodialysis with a tunneled catheter or other form of renal replacement (**S1a Fig**). We included transplanted patients in our model, and thus the “other form of renal replacement” health state is a combination of patients undergoing hemodialysis without a tunneled catheter, patients undergoing peritoneal dialysis, and patients with a kidney transplant. Prior literature suggests that MDC programs that focus on dialysis planning (typically in patients with late CKD stage 4 and CKD stage 5) are successful in reducing the rate of tunneled dialysis catheter use but are mixed on its effect on the use of peritoneal dialysis or transplant. We therefore did not split the ESRD health state further and only modeled the tunneled dialysis catheter health state separately.

After calibrating the development model, we recombined the two ESRD health states into a single ESRD health state to form the final model.


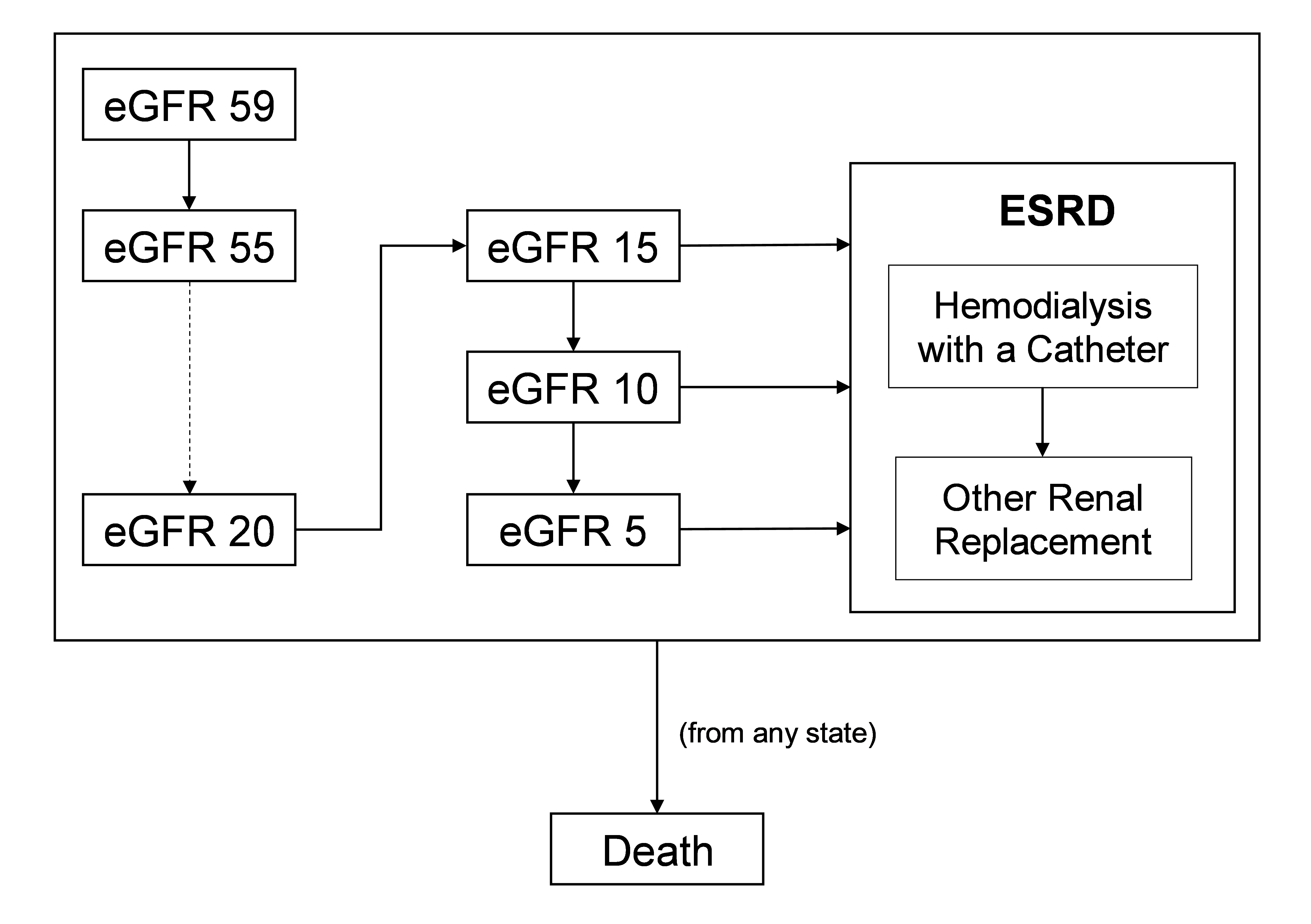


**S1a Fig: Developmental Model for Simulating Chronic Kidney Disease Progression.** We used the developmental model to calibrate all parameters. We modeled progression of chronic kidney disease (CKD) using levels of estimated glomerular filtration rate (eGFR). For simplicity, we modeled each level of eGFR in multiples of 5 mL/min/1.73 m^2^. Each cycle, patients have a probability of staying at the same eGFR level or dropping to the next eGFR. Between eGFRs of 5 and 15 mL/min/1.73 m^2^, patients also have the possibility of progressing to end-stage renal disease (ESRD). New onset ESRD can occur either with a dialysis catheter or without. Death can occur at any point. Abbreviations: eGFR = estimated glomerular filtration rate, CKD = chronic kidney disease, ESRD = end-stage renal disease

Because we were interested in modeling CKD progression in subpopulations stratified by age (45-64, 65-74, 75-84 years old), sex (male/female), race (white, black, other), starting urine albumin to creatinine ratio (UACR) (1, 300, 1000, and 3000 mg/g), and starting eGFR (59 to 20 mL/min/1.73 m^2^), we developed individual models for each group. Since patients age as they run through the simulation, we computed transition probabilities as dependent on age in decades. We employed a half cycle correction in our models and discounted all costs and QALYs at a rate of 3% per year, or 0.247% for a cycle length of one month.

### Overall Calibration Strategy

In order to reduce the degrees of freedom of our model and to smooth the progression rate of kidney disease, we assumed that the transition probabilities for progression to the next eGFR level was the same for eGFRs of 59 to 35, 30 to 20, and 15 to 10 mL/min/1.73 m^2^. We also assumed that the transition probability of developing ESRD from CKD was the same for eGFRs of 15 to 5 mL/min/1.73 m^2^. Conditional on age, sex, race, and UACR, we developed transition probability matrices with 19 unknowns each. As we describe later, we were able to directly estimate the proportion of incident patients who start with hemodialysis using a tunneled catheter, denoted as ‘a’ in **S1b** **Table**.

**S1b Table: Usual Care Transition Probability Matrix**

|  | **Ending Health State** | | | | | | | | | | | | | | |
| --- | --- | --- | --- | --- | --- | --- | --- | --- | --- | --- | --- | --- | --- | --- | --- |
| **Starting Health State** | **1** | **2** | **3** | **4** | **5** | **6** | **7** | **8** | **9** | **10** | **11** | **12** | **13** | **14** | **15** |
| 1. **eGFR 59** | ~ | P_1_ |  |  |  |  |  |  |  |  |  |  |  |  | D_1_ |
| 1. **eGFR 55** |  | ~ | P_1_ |  |  |  |  |  |  |  |  |  |  |  | D_2_ |
| 1. **eGFR 50** |  |  | ~ | P_1_ |  |  |  |  |  |  |  |  |  |  | D_3_ |
| 1. **eGFR 45** |  |  |  | ~ | P_1_ |  |  |  |  |  |  |  |  |  | D_4_ |
| 1. **eGFR 40** |  |  |  |  | ~ | P_1_ |  |  |  |  |  |  |  |  | D_5_ |
| 1. **eGFR 35** |  |  |  |  |  | ~ | P_1_ |  |  |  |  |  |  |  | D_6_ |
| 1. **eGFR 30** |  |  |  |  |  |  | ~ | P_2_ |  |  |  |  |  |  | D_7_ |
| 1. **eGFR 25** |  |  |  |  |  |  |  | ~ | P_2_ |  |  |  |  |  | D_8_ |
| 1. **eGFR 20** |  |  |  |  |  |  |  |  | ~ | P_2_ |  |  |  |  | D_9_ |
| 1. **eGFR 15** |  |  |  |  |  |  |  |  |  | ~ | P_3_ |  | a*E | (1-a)*E | D_10_ |
| 1. **eGFR 10** |  |  |  |  |  |  |  |  |  |  | ~ | P_3_ | a*E | (1-a)*E | D_11_ |
| 1. **eGFR 5** |  |  |  |  |  |  |  |  |  |  |  | ~ | a*E | (1-a)*E | D_12_ |
| 1. **HD with Catheter** |  |  |  |  |  |  |  |  |  |  |  |  | ~ | T | D_13_ |
| 1. **Other ESRD** |  |  |  |  |  |  |  |  |  |  |  |  |  | ~ | D_14_ |
| 1. **Death** |  |  |  |  |  |  |  |  |  |  |  |  |  |  | 1 |

P_i_ is the probability of transitioning from one eGFR state to the next (i=1 for eGFRs 59-35, 2 for eGFRs 30-20, 3 for eGFRs 15-5)

D_j_ is the probability of death from state j

E is the probability of transitioning from CKD (eGFR 15-5) to ESRD

a is the proportion of incident ESRD patients who hemodialysis with a tunneled catheter

T is the probability of moving from the hemodialysis with tunneled catheter state to ESRD without a tunneled catheter

~ is a positive number between 0 and 1 to ensure that each row sums to 1

Blank cells mean a transition probability of 0.

Estimating monthly transition probabilities is challenging given the positive correlation between severity of CKD and mortality. Ignoring these correlations would likely lead to overestimates of long-term rates of mortality and progression to ESRD. To address this concern, we calibrated each model to the following long-term targets of mortality and ESRD progression:

1. The 2-year and 5-year probabilities of developing ESRD as functions of age, sex, eGFR and UACR.
2. The 1-, 2-, 3-, 4-, 5-, and 10-year probabilities of mortality for patients with CKD as functions of age, sex, race, eGFR, and UACR.
3. The proportion of patients with new-onset ESRD who start hemodialysis with a dialysis catheter.
4. The 1-, 2-, and 3-year probabilities of mortality for patients with ESRD (patients starting hemodialysis with a tunneled catheter and all other patients with ESRD) as functions of age, sex, and race.
5. For patients undergoing hemodialysis with a tunneled catheter, the 1-, 2-, and 3-year probabilities of transitioning off catheter as functions of age, sex, and race. As described above, transitioning off catheter meant undergoing hemodialysis through an arteriovenous graft or fistula, receiving peritoneal dialysis, or having a functional transplant.

To compute the vector of transition probabilities for each model, we used a constrained Nelder-Mead algorithm that minimized the sum of percent differences between actual and computed long-term probability targets.[1] We chose a Nelder-Mead algorithm because it does not rely on gradients to minimize the objective function. More formally, we solved for X, the vector of transition probabilities, using the following objective function:

$$\min_{X} \sum_{i} \left[ \frac{\hat{f_{i}(X)}-f_{i}}{f_{i}} \right]^{2}$$

where $f_{i}$ is the actual probability of event ‘i’ (progression to ESRD or mortality for a given time frame) and $\hat{f_{i}(X)}$ is the Markov-model computed probability of event ‘i’ for the transition vector X. Each transition matrix is unique for a given age, sex, race, and UACR, but eGFR can vary within a transition matrix. Therefore, for a given age, sex, race, and UACR, we defined the set I (with $i\in I$) as the set of the following events:

1. The 2-year and 5-year probabilities of developing ESRD for patients with the starting eGFRs of 59 through 20 mL/min/1.73 m^2^.
2. The 1-, 2-, 3-, 4-, 5-, and 10-year probabilities of death for patients with the starting eGFRs of 59 through 20 mL/min/1.73 m^2^.
3. The 1-, 2-, and 3-year probabilities of mortality for patients starting hemodialysis with a tunneled catheter and for patients with other ESRD (hemodialysis without a tunneled catheter, peritoneal dialysis, and transplant).
4. For patients starting hemodialysis with a tunneled catheter, the 1-, 2-, and 3-year probabilities of having the catheter removed.

We chose an objective function that used percent differences (rather than squared differences or absolute value of differences) because our probability targets encompassed a wide range of values. Normalizing the differences ensured that we were not overly penalizing targets of larger magnitude. We chose to weight each objective probability equally because *a priori* we did not have a preference for one probability target over another.

### Calibration Target: Long-term Probability of Developing ESRD for Patients with CKD

We used the model developed by Tangri *et al*. to estimate the 2-year and 5-year probabilities of patients with CKD developing ESRD.[2] These probabilities were computed as functions of age, sex, eGFR, and albuminuria. Although the authors published the 95% confidence intervals for the coefficients (**S1c Table**), we could not directly compute confidence intervals for the predicted probabilities because we did not have the covariance matrix.

**S1c Table: Hazard Ratios for Computing the Long-Term Risk of ESRD**

| **Covariate** | **Coefficient** | **95% CI** |
| --- | --- | --- |
| Age (by decade) | 0.80 | 0.75, 0.86 |
| Male | 1.28 | 1.04, 1.58 |
| eGFR (per 5 cc/min/1.73 m^2^) | 0.57 | 0.54, 0.61 |
| Albuminuria (per log increase) | 1.57 | 1.44, 1.71 |

Instead, we approximated the 95% confidence interval of these predicted probabilities by computing the maximum and minimum probabilities when using the confidence intervals for each coefficient. To do this, we substituted coefficient values from the 95% confidence intervals into the following equation, from Tangri *et al.*:

$$P_{ESRD,i}=1-H_{i}^{exp(XB)}$$

where $H_{2}=0.975$ is the baseline hazard rate for developing ESRD in 2 years and $H_{5}=0.924$ is the baseline hazard rate for developing ESRD in 5 years and

$$XB=\beta_{age}*\left( \frac{age}{10}-7.036 \right)+\beta_{male}*\left( 1_{male}-0.5642 \right)+\beta_{eGFR}*\left( \frac{eGFR}{5}-7.222 \right)+\beta_{UACR}*\left( \ln\left( UACR \right)-5.137 \right)$$

### Calibration Target: Long-term Probability of Mortality for Patients with CKD

We first estimated the probability of dying within 1, 2, 3, 4, 5, and 10 years for patients with normal kidney function, stratified by age, sex, and race, using the Centers for Disease Control and Prevention (CDC) life tables.[3] Because life tables only exist for white and black people, we assumed that people of “other” race had the same life expectancy as white people.

We then applied hazard ratios based on eGFR and albuminuria from van der Velde *et al*. to these long-term probabilities of death.[4] Although the authors published hazard ratios for specific eGFR and albuminuria groups, their cutoffs did not correspond to our subgroups of interest. We therefore extracted the coefficients and 95% confidence intervals from Figure 1 of this paper using WebPlotDigitizer (<http://arohatgi.info/WebPlotDigitizer/>), a tool which extracts data from scientific images (**S1d Table**).

**S1d Table: Hazard Ratio Estimates for Mortality**

| **Characteristic** | **Hazard Ratio** | **95% CI** |
| --- | --- | --- |
| eGFR = 59 cc/min/1.73 m^2^ | 1.03 | 0.81, 1.33 |
| eGFR = 45 cc/min/1.73 m^2^ | 1.38 | 1.15, 1.65 |
| eGFR = 15 cc/min/1.73 m^2^ | 3.11 | 2.26, 4.27 |
| Albuminuria = 5 mg/g | 1 | None (Reference) |
| Albuminuria = 10 mg/g | 1.08 | 1.01, 1.16 |
| Albuminuria = 30 mg/g | 1.38 | 1.23, 1.56 |
| Albuminuria = 300 mg/g | 2.16 | 1.99, 2.35 |

The log of the hazard ratios appeared to vary linearly with eGFR (between 15 to 59 mL/min/1.73 m^2^) and with the log of albuminuria. Therefore, we generated lines of best fit for the point estimates and lower and upper confidence intervals for eGFR and albuminuria. Our estimates yielded the following equations:

$$\ln\left( HR_{death}(eGFR) \right)=Intercept_{death,eGFR}+\gamma_{eGFR}*eGFR$$

$$\ln\left( HR_{death}(UACR) \right)=Intercept_{death,UACR}+\gamma_{UACR}*\ln\left( UACR \right)$$

where the coefficients are given in **S1e Table**.

**S1e Table: Coefficients for Computing Hazard Ratios for Death**

| **Parameter** | **Intercept** | **γ (slope)** |
| --- | --- | --- |
| Hazard Ratio (eGFR), point estimate | 1.4921 | -0.0249 |
| Hazard Ratio (eGFR), 2.5% quantile | 1.1586 | -0.0228 |
| Hazard Ratio (eGFR), 97.5% quantile | 1.8157 | -0.0267 |
| Hazard Ratio (UACR), point estimate | -0.3362 | 0.1931 |
| Hazard Ratio (UACR), 2.5% quantile | -0.4621 | 0.2009 |
| Hazard Ratio (UACR), 97.5% quantile | -0.2895 | 0.2030 |

After computing hazard ratios for each of the eGFRs and urine albumin excretion values, we then applied them to the CDC mortality estimates to obtain point estimates and 95% confidence intervals for the 1, 2, 3, 4, 5, and 10 year probability of death conditional on age, sex, race, eGFR and urine albumin to creatinine ratio. We did this by assuming that these probability distributions followed an exponential distribution. In other words:

$$Pr_{y} \left( death|age,sex,race,eGFR,UACR \right)=1-e^{HR_{UACR}*HR_{eGFR}*\ln\left( 1-Pr_{y}(death|age,sex,race) \right)}$$

where $Pr_{y} \left( death|age,sex,race,eGFR,UACR \right)$ is the probability of death in ‘y’ years for a given age, race, sex, eGFR, and UACR and $Pr_{y}(death|age,sex,race)$ is the probability of death in ‘y’ years for a given age, race, and sex for a person without kidney disease (from CDC life tables).

### Calibration Target: Proportion of Incident Patients Starting Hemodialysis with a Catheter

We used data from the United States Renal Data System (USRDS) to compute the proportion of incident patients starting hemodialysis with a catheter.[5] We did this by taking all adult patients who had their first day of ESRD between July 1, 2010 and November 30, 2013 and who had Medicare Parts A and B at day 1. We made the latter restriction because we were studying a theoretical Medicare-funded MDC program. We included patients who received a preemptive transplant (i.e., kidney transplant prior to any dialysis) in the sample. We used the Medical Evidence Form (CMS 2728) to determine if a patient started hemodialysis with a tunneled catheter. To compute a 95% confidence interval around this estimate, we bootstrapped 10,000 samples, which yielded a point estimate of 0.7247 with a 95% CI of [0.7232, 0.7261].

### Calibration Target: Long-term Probability of Mortality for Patients with ESRD

Using the same patient population from the USRDS, we obtained the date of death. For both ESRD subpopulations (patients who started hemodialysis with a catheter and all other ESRD patients), we modeled time to death using the Kaplan-Meier estimator, stratifying by age, sex, and race and censoring for end of the follow-up period. We did not adjust for any other factors. From this model, we obtained point estimates and 95% CI’s for the long-term probability of death.

### Calibration Target: Likelihood of Catheter Removal

For patients who started hemodialysis with a catheter, we determined the dates of the following events: first transplant, change in modality to peritoneal dialysis, first use of arteriovenous fistula (AVF) or graft (AVG), and death. We used the patient database to determine the dates of transplant and death. The USRDS has a longitudinal treatment database, which we used to obtain the date that a patient switched to peritoneal dialysis. We used outpatient dialysis 72x claims to determine type of dialysis access. A modifier code of V5 in conjunction with a dialysis claim signified dialysis with a vascular catheter. Modifier codes V6 and V7 corresponded to AVG and AVF respectively. We then used the Kaplan-Meier estimator, stratified by age, sex, and race, to model the time to first catheter removal, which we defined as the composite outcome of hemodialysis with an AVF or AVG, transplant, or change to peritoneal dialysis, censoring for death and end of follow-up.

### Calibration Results

For our base case, we solved for the vector of transition probabilities for each set I, conditional on age, sex, race, and UACR. We manually inspected each calibrated model to ensure that they yielded reasonable long-term estimates of mortality and progression to ESRD (**S13** **Appendix**). In these figures, we compare the probability targets to the estimated probability from our Markov models. We show 95% confidence intervals for each of the probability targets.

## Analysis 2: Modeling the Cost-Effectiveness of MDC

### Estimating the Effectiveness of MDC

To estimate transition probabilities for patients receiving MDC, we first took the transition probability matrices computed under usual care. We then assumed that MDC could modify these transition probabilities along one of 3 dimensions: (1) CKD progression, (2) CKD mortality, and (3) transitioning from CKD to ESRD. For each CKD progression model, we assigned 3 unknowns, α_1_, α_2_, and α_3_ which corresponded to each of these dimensions respectively. More formally, the MDC transition probabilities can be expressed as in **S1f Table**:

**S1f Table: MDC Transition Probability Matrix**

|  | **Ending Health State** | | | | | | | | | | | | | | |
| --- | --- | --- | --- | --- | --- | --- | --- | --- | --- | --- | --- | --- | --- | --- | --- |
| **Starting Health State** | **1** | **2** | **3** | **4** | **5** | **6** | **7** | **8** | **9** | **10** | **11** | **12** | **13** | **14** | **15** |
| 1. **eGFR 59** | ~ | α_1_*P_1_ |  |  |  |  |  |  |  |  |  |  |  |  | α_3_*D_1_ |
| 1. **eGFR 55** |  | ~ | α_1_*P_1_ |  |  |  |  |  |  |  |  |  |  |  | α_3_*D_2_ |
| 1. **eGFR 50** |  |  | ~ | α_1_*P_1_ |  |  |  |  |  |  |  |  |  |  | α_3_*D_3_ |
| 1. **eGFR 45** |  |  |  | ~ | α_1_*P_1_ |  |  |  |  |  |  |  |  |  | α_3_*D_4_ |
| 1. **eGFR 40** |  |  |  |  | ~ | α_1_*P_1_ |  |  |  |  |  |  |  |  | α_3_*D_5_ |
| 1. **eGFR 35** |  |  |  |  |  | ~ | α_1_*P_1_ |  |  |  |  |  |  |  | α_3_*D_6_ |
| 1. **eGFR 30** |  |  |  |  |  |  | ~ | α_1_*P_2_ |  |  |  |  |  |  | α_3_*D_7_ |
| 1. **eGFR 25** |  |  |  |  |  |  |  | ~ | α_1_*P_2_ |  |  |  |  |  | α_3_*D_8_ |
| 1. **eGFR 20** |  |  |  |  |  |  |  |  | ~ | α_1_*P_2_ |  |  |  |  | α_3_*D_9_ |
| 1. **eGFR 15** |  |  |  |  |  |  |  |  |  | ~ | α_1_*P_3_ |  | α_2_*a*E | α_2_*(1-a)*E | α_3_*D_10_ |
| 1. **eGFR 10** |  |  |  |  |  |  |  |  |  |  | ~ | α_1_*P_3_ | α_2_*a*E | α_2_*(1-a)*E | α_3_*D_11_ |
| 1. **eGFR 5** |  |  |  |  |  |  |  |  |  |  |  | ~ | α_2_*a*E | α_2_*(1-a)*E | α_3_*D_12_ |
| 1. **HD with Catheter** |  |  |  |  |  |  |  |  |  |  |  |  | ~ | T | D_13_ |
| 1. **Other ESRD** |  |  |  |  |  |  |  |  |  |  |  |  |  | ~ | D_14_ |
| 1. **Death** |  |  |  |  |  |  |  |  |  |  |  |  |  |  | 1 |

P_i_ is the probability of transitioning from one eGFR state to the next under the control case (i=1 for eGFRs 59-35, 2 for eGFRs 30-20, 3 for eGFRs 15-5)

D_j_ is the probability of death from state j under the control

E is the probability of transitioning from CKD (eGFR 15-5) to ESRD under the control

a is the proportion of incident ESRD patients who hemodialysis with a tunneled catheter

T is the probability of moving from the hemodialysis with tunneled catheter state to ESRD without a tunneled catheter

α_1_ is the factor that MDC slows progression of CKD

α_2_ is the factor that MDC reduces mortality of CKD

α_3_ is the factor that MDC reduces the transition from CKD to ESRD

~ is a positive number between 0 and 1 to ensure that each row sums to 1

Blank cells mean a transition probability of 0.

For each subpopulation, we found the set of α_1_, α_2_, α_3_, which gave odds ratios for mortality and progression to ESRD similar to those published in Wang *et al*. over 4.9 years of follow-up.[6] More formally, we solved for the vector A = {α_1_, α_2_, α_3_}, using a constrained Nelder-Mead algorithm, which minimized the following function:

$$\min_{A} \sum_{j\in J} \left[ \left( \frac{{\frac{g_{j}\left( A,X \right)}{1-g_{j}\left( A,X \right)}}/{\frac{g_{j}\left( 1,X \right)}{1-g_{j}\left( 1,X \right)}}-OR_{mortality}}{OR_{mortality}} \right)^{2}+\left( \frac{{\frac{h_{j}\left( A,X \right)}{1-h_{j}\left( A,X \right)}}/{\frac{h_{j}\left( 1,X \right)}{1-h_{j}\left( 1,X \right)}}-OR_{ESRD}}{OR_{ESRD}} \right)^{2} \right]$$

where

J is the set of populations with starting eGFRs from 20 to 59 mL/min/1.73 m^2^ given a specific age, sex, race, and UACR,

X is the vector of transition probabilities under usual care for a given age, sex, race, and UACR,

A = {α_1_, α_2_, α_3_} is the vector of MDC factors as defined above,

$g_{j}\left( A,X \right)$ is the probability of mortality in 4.9 years predicted by the Markov model given the usual care transition probability vector X and the MDC factor vector A for the population j,

$h_{j}\left( A,X \right)$ is the probability of ESRD in 4.9 years predicted by the Markov model given the usual care transition probability vector X and the MDC vector A for the population j,

$OR_{mortality}$ is the odds ratio for mortality: 0.62 (95% CI: 0.44, 0.88),

$OR_{ESRD}$ is the odds ratio for progression to ESRD: 0.59 (95% CI: 0.38, 0.92).

This process yielded transition probabilities for an MDC program that was 100% effective.

### Discounting the Effectiveness of MDC under the Base Case

The published literature for MDC is of limited quality. Most of the included studies in the Wang *et al*. meta-analysis were observation or single-center randomized trials.[6] Although the majority were published in the last decade, some were published ten to twenty years ago. Furthermore, none of the studies stratified the effect of MDC by CKD stage, even though in aggregate, they included patients with CKD stages 3 through 5.

Given these limitations, we discounted effectiveness estimates of MDC in earlier CKD stages, using the intuition that MDC is less effective in milder forms of CKD. We started by calibrating the effectiveness of MDC to odds ratios reported in Wang *et al*. using the previously described calibration method: 0.62 for mortality and 0.59 for progression to ESRD over a 4.9 year follow-up period (**Table 1**).[6] This process yielded the transition probabilities estimated above in **S1f Table**, estimated for a 100% effective MDC program. For the base case, we then adjusted our model, so that MDC was 25% effective in CKD stage 3, 50% effective in CKD stage 4, and 100% effective in CKD stage 5.

To vary the effectiveness of MDC in different stages of CKD, we defined $\delta$, or the effectiveness of the MDC program. More formally, given a base case transition probability P_i_ and the corresponding MDC transition probability $\alpha_{i}*P_{i}$, the amount that MDC decreases the transition probability is $\left( 1-\alpha_{i} \right)*P_{i}$. If the MDC program instead operates at an effectiveness of $\delta\in[0, 1]$, the amount that MDC decreases the transition probability is $\left( 1-\alpha_{i} \right)*P_{i}*\delta$. Then the transition probability of the inefficient MDC program is $\left( 1-\delta+\alpha_{i}*\delta\right)*P_{i}$. (Notably, for ESRD patients, we assume that MDC has no effect on their transition probabilities and thus assume $\alpha_{i}=1$. For these transition probabilities, $\left( 1-\delta+\alpha_{i}*\delta\right)*P_{i}=P_{i}$ for all $\delta$). We then varied $\delta$ for different CKD stages to obtain a program that was 25% effective in CKD stage 3, 50% effective in CKD stage 4, and 100% effective in CKD stage 5.

Since percent effectiveness measured as a discounted transition probability is difficult to interpret, we computed hazard ratios for death and progression to ESRD in each subgroup. To do this, we simulated outcomes for 10,000 usual care patients and 10,000 MDC patients.

The large degree of uncertainty around the program’s effectiveness prompted us to vary these assumptions in sensitivity analyses, which we describe in later sections.

### Estimating Costs of Usual Care

We estimated the costs of CKD and ESRD care using data from the USRDS, which contains Medicare Parts A and B claims for 5% of the Medicare population with CKD and 100% of the Medicare population with ESRD.

For patients with CKD, we first identified those greater than or equal to 25 years old who had continuous parts A and B coverage for the entirety of 2011 to 2013. This allowed for a full year of claims to determine the stage of CKD and a second year of claims to compute costs. We excluded patients not in the US or who had previous dialysis or transplant.

Because Medicare claims do not contain laboratory data, we could not calculate costs for specific eGFRs. We instead summarized costs by CKD stage. For the first calendar year of each cohort, we identified patients with at least one institutional claim or at least two non-institutional claims with one of the following ICD-9 diagnosis codes: 585.3 (CKD stage 3), 585.4 (CKD stage 4), 585.5 (CKD stage 5). This allowed us to categorize patients by CKD stage. Ties were broken by using the latest appearing code.

For each patient, we summed the costs of all claims in the second calendar year after inflation adjusting to the US dollar value in January, 2017.[7] Because we were interested in estimating the distribution of costs stratified by age, sex, race, and CKD stage, we modeled cost as a linear outcome dependent on each of the aforementioned subcategories. We converted age into a categorical variable: 25 to 44, 45 to 64, 65 to 74, 75 to 84, 85 to 94, and greater than 94 years old. We also combined CKD stages 4 and 5 into a single category given the low population count of patients with CKD stage 5 (910 patients from the 5% sample, with some subcategories having only 1 patient). From our regression estimates (**S1g Table**), we computed the estimated average cost for each subpopulation and used the delta method with robust standard errors to estimate 95% confidence intervals.

**S1g Table: Regression Estimates of Annual Medicare Costs of Patients with CKD**

| **Characteristic** | **Coefficient** | **95% Confidence Interval** | |
| --- | --- | --- | --- |
| Intercept | $26,674 | $18,719 | $34,630 |
| **Sex** |  |  |  |
| Female | -- |  |  |
| Male | $1,236 | $820 | $1,652 |
| **Race** |  |  |  |
| White | -- |  |  |
| Black | $1,453 | $773 | $2,134 |
| Other | - $108 | - $1,080 | $863 |
| **CKD Stage** |  |  |  |
| Stage 3 | -- |  |  |
| Stage 4-5 | $3,846 | $3,219 | $4,472 |
| **Age (years)** |  |  |  |
| 25-44 | -- |  |  |
| 45-64 | - $7,520 | - $15,595 | $554 |
| 65-74 | - $11,503 | - $19,496 | - $3,510 |
| 75-84 | - $12,173 | - $20,152 | - $4,193 |
| 85-94 | - $10,483 | - $18,457 | - $2,508 |
| 95+ | - $9,918 | - $18,073 | - $1,763 |

Rounded to nearest dollar

-- Denotes reference case

We were interested in capturing the costs of ESRD for both groups of patients: those undergoing hemodialysis with a tunneled catheter and those with ESRD but without a tunneled catheter. To do this, we combined two populations. First, we constructed the cohort of patients who were undergoing hemodialysis with a tunneled catheter. We did this by taking patients who had continuous Medicare Parts A and B coverage with the first date of dialysis after January 1, 2012 and who started hemodialysis with a tunneled catheter according to the CMS 2728 form. We also determined the date when these patients exited the sample: when they switched to peritoneal dialysis, received a transplant, died, or switched to an AVF or AVG. Second, to find patients with ESRD without a tunneled catheter, we again took patients with continuous Medicare Parts A and B coverage. We then included the following groups of patients: (i) patients with first date of ESRD after January 1, 2012, starting either peritoneal dialysis or preemptive transplant, (ii) patients with first date of ESRD after January 1, 2012 and starting hemodialysis using an AVF or AVG according to the CMS 2728 form, and (iii) patients already on hemodialysis who switched to an AVF or AVG after January 1, 2012. We also determined the date when these patients died, which is when they exited the sample. Of note, patients could contribute an observation to each of the two subcohorts.

For each patient in this combined group, we totaled the inflation-adjusted costs (to the value of the US dollar in January 1, 2017) of all claims between the start and exit date. After normalizing these costs to 365 days, we modeled annual cost as a linear outcome dependent on age, sex, race, and whether the patient had a tunneled catheter, with robust standard errors. Using these regression estimates (**S1h Table**), we computed the estimated average cost for each subpopulation and used the delta method to estimate 95% confidence intervals.

**S1h Table: Regression Estimates of Annual Medicare Costs of Patients with ESRD**

| **Characteristic** | **Coefficient** | **95% Confidence Interval** | |
| --- | --- | --- | --- |
| Intercept | $37,004 | $34,925 | $39,084 |
| **Sex** |  |  |  |
| Female | -- |  |  |
| Male | - $1,681 | - $2,884 | - $479 |
| **Race** |  |  |  |
| White | -- |  |  |
| Black | - $2,607 | - $3,930 | - $1,284 |
| Other | - $5,289 | - $8,311 | - $2,266 |
| **Tunneled Catheter** |  |  |  |
| No | -- |  |  |
| Yes | $38,251 | $37,052 | $39,451 |
| **Age (years)** |  |  |  |
| 25-44 | -- |  |  |
| 45-64 | $10,087 | - $8,173 | $12,001 |
| 65-74 | $35,564 | $33,442 | $37,687 |
| 75-84 | $43,101 | $40,892 | $45,311 |
| 85+ | $50,777 | $47,649 | $53,905 |

Rounded to nearest dollar

-- Denotes reference case

To compute Part D costs, we used the Annual Data Report (ADR) from the USRDS, which gives the average annual costs by age, race, and whether patients were on the low-income subsidy (LIS).[8,9] We took the estimates for 2013, inflation adjusted them to the value of the US dollar in January 1, 2017, and computed the weighted average of annual costs for CKD and ESRD, stratified by age and sex (**S1i Table**). These estimates did not have confidence intervals, so we used the point estimates.

**S1i Table: Annual Part D Costs for CKD and ESRD**

| **Age** | **Race** | **CKD Cost** | **ESRD Cost** |
| --- | --- | --- | --- |
| 45-64 | White | $7,877 | $6,891 |
| 45-64 | Black | $8,309 | $7,476 |
| 45-64 | Other | $8,252 | $7,229 |
| 65-74 | White | $2.933 | $4,718 |
| 65-74 | Black | $3,893 | $5,699 |
| 65-74 | Other | $3,394 | $5,769 |
| 75-84 | White | $1,968 | $3,469 |
| 75-84 | Black | $2,830 | $4,504 |
| 75-84 | Other | $2,620 | $4,801 |

Rounded to the nearest dollar

### Estimating the Costs of MDC

In the literature, MDC spans a myriad of interventions ranging from conservative (nursing only) to robust (nursing, advanced practitioner, pharmacist, dietician, and social worker). It increases in intensity through increased provider visits as kidney function worsens and stops once patients progress to ESRD.

Because many studies did not explicitly describe each activity, we were unable to disentangle the effect of each component of MDC. Although our effectiveness estimates from Wang *et al*. represent the average effectiveness of MDC, we calculated MDC costs based on the most expensive program documented.[6] This ensured our cost-effectiveness estimates were conservative.

The most expensive MDC program was described by Levin *et al.*, so we matched each MDC activity to a Current Procedural Terminology (CPT) code and its corresponding cost from the 2017 Medicare Physician Fee Schedule (**Tables 1-2**).[10,11] We assumed that patients receiving MDC had an initial visit cost of $273.11 and subsequent recurring costs that increased with progression of CKD. Because Levin *et al*. did not detail all aspects of their intervention, especially the amount of time per visit, we made assumptions based on their qualitative description. The authors indicated that caring for a patient receiving usual care took on average 7 to 15 hours a year depending on CKD severity. On the other hand, caring for patients on MDC averaged 15 to 33 hours a year. Extrapolating, we attempted to ensure that our program added close to 8 to 18 hours per year over usual care. Our program added 5.5, 10.5, and 14.5 hours annually for CKD stages 3, 4, and 5 respectively, with an additional 2 hours for the initial visit.

In general, published studies did not investigate whether MDC increased other healthcare expenses, such as medications and laboratory tests. We were unable to use data to estimate these costs because of the paucity of data for these services. Instead, we accounted for this uncertainty by assuming that MDC increased the use of medications and laboratory tests for our base case (**Tables 1 and 3**). Specifically, we assumed that MDC led to the use of epoetin alfa 5,000 units per week and calcitriol 1 mcg three times a week in 25% of patients with an eGFR of 30 mL/min/1.73 m^2^ and below. We obtained the prices of these drugs from the Medicare Part B Physician Fee Schedule.[10] Although many patients receive oral Vitamin D, we priced the injectable version because it is more expensive and we did not have access to oral medication prices in Medicare. Since estimating the baseline number of patients on erythropoietin and activated Vitamin D is difficult, we chose to use an absolute increase of medication use in the population, rather than a relative increase. We used 25% of the population because it was an overestimate of this increase, making our cost-effectiveness estimates more conservative.

We also assumed that for our base case, MDC increased laboratory testing in 25% of patients with an eGFR of less than 45 mL/min/1.73 m^2^ and below. We assumed that these patients received a standard battery of CKD-related laboratory tests (complete metabolic panel, phosphorus, complete blood count, iron panel, ferritin, Vitamin D, and intact parathyroid hormone) every 3 months.

Since we were uncertain of the degree that MDC increased the use of medications and laboratory tests, we varied these assumptions widely in sensitivity analyses.

### Health Outcomes and Their Valuation

As described in the Methods, we limited the scope of our study to CKD progression because the literature has not definitively shown that MDC has effectively mitigated the risk of hospitalizations. We also chose not to study the effect on tunneled catheter rates because our theoretical intervention focused on CKD stages 3 and 4 before most patients consider dialysis access placement.

We used QALY’s from a recent systematic review by Wyld *et al*., to estimate the utilities associated with CKD and ESRD.[12] Although Wyld *et al*. did not differentiate between the different stages of CKD, published literature do not show substantial differences in health-related quality of life across different CKD stages.[13,14] We therefore used the estimate of 0.80 QALYs for CKD (95% CI: 0.70, 0.90) and 0.71 QALYs for ESRD (95% CI: 0.62, 0.80) (**Table 1**).

We determined the cost-effectiveness of MDC for Medicare by computing the incremental cost-effectiveness ratio (ICER), or the quotient of the change in total lifetime cost (MDC *versus* usual care) divided by the change in total lifetime QALYs. For our base case, we assumed a willingness to pay (WTP) of $150,000 per QALY gained.

To obtain estimates for population aggregates (e.g., all females or all patients 45-64 years old), we took the weighted average of the above outcomes, using population counts by CKD stage, age, sex, and race, which we obtained from the USRDS. For ICERs, we first took the weighted average of the change in costs and change in QALYs, then took the ratio of these averages.

## Analysis 3: Sensitivity Analyses

As described in the Methods, there was a high degree of uncertainty surrounding estimates of effectiveness and cost of MDC. Therefore, we repeated our analyses where we extensively varied the effectiveness and the cost of MDC (**Table 4**).

*Varying the Effectiveness of MDC*

We varied the effectiveness of MDC by varying $\delta$ (defined previously as the effectiveness of MDC) under the following scenarios:

1. 50% of the base case:

$$\delta=\left\{ \begin{aligned} 50\% for CKD stage 3 \\ 25\% for CKD stage 4 \\ 12.5\% for CKD stage 5 \end{aligned} \right.$$

2. 25% of the base case:

$$\delta=\left\{ \begin{aligned} 50\% for CKD stage 3 \\ 25\% for CKD stage 4 \\ 12.5\% for CKD stage 5 \end{aligned} \right.$$

3. 100%, Non-Discounted: $\delta=100\% for all CKD stages$
4. 50%, Non-Discounted: $\delta=50\% for all CKD stages$
5. 25%, Non-Discounted: $\delta=25\% for all CKD stages$
6. Only mortality, base case:

$$\delta=\left\{ \begin{aligned} 100\% for CKD stage 3, mortality \\ 50\% for CKD stage 4, mortality \\ 25\% for CKD stage 5, mortality \\ 0\% for ESRD progression \end{aligned} \right.$$

7. Only mortality, 50% of the base case:

$$\delta=\left\{ \begin{aligned} 50\% for CKD stage 3, mortality \\ 25\% for CKD stage 4, mortality \\ 12.5\% for CKD stage 5, mortality \\ 0\% for ESRD progression \end{aligned} \right.$$

8. Only mortality, 25% of the base case:

$$\delta=\left\{ \begin{aligned} 25\% for CKD stage 3, mortality \\ 12.5\% for CKD stage 4, mortality \\ 6.25\% for CKD stage 5, mortality \\ 0\% for ESRD progression \end{aligned} \right.$$

From these scenarios we calculated the cost-effectiveness. Because effectiveness computed in terms of transition probabilities is difficult to understand conceptually, we also estimated the hazard ratios for death and progression to ESRD for these scenarios. We did this by simulating 10,000 usual care patients and 10,000 MDC patients and comparing their outcomes with an unadjusted Cox proportional hazards model.

*Varying the Cost of MDC*

We then computed different scenarios for which MDC had different costs:

1. MDC did not lead to any increase in laboratory tests or medications and did not change in cost.
2. MDC led to an increase in laboratory tests in 10% of patients with an eGFR ≤ 45 mL/min/1.73 m^2^ and medications in 10% of patients with an eGFR ≤ 30 mL/min/1.73 m^2^. These medications and laboratory tests were previously defined in **Table 3**.
3. MDC led to an increase in laboratory tests in 50% of patients with an eGFR ≤ 45 mL/min/1.73 m^2^ and medications in 50% of patients with an eGFR ≤ 30 mL/min/1.73 m^2^.
4. MDC led to an increase in laboratory tests in 100% of patients with an eGFR ≤ 45 mL/min/1.73 m^2^ and medications in 100% of patients with an eGFR ≤ 30 mL/min/1.73 m^2^.
5. The initial cost of MDC remained fixed, but all recurring costs increased two-fold (this is analogous to patients receiving twice the visits).
6. The initial cost of MDC remained fixed, but all recurring costs increased five-fold.

For each scenario we computed the cost-effectiveness of MDC. Since varying the costs did not change the effectiveness of MDC, we did not compute hazard ratios.

### Probabilistic Sensitivity Analysis

We then performed probabilistic sensitivity analyses for the base case and for each of the above effectiveness and cost scenarios.

To compute the joint distribution for usual care transition probabilities, we fit our target probabilities to beta distributions according to their 95% confidence intervals. More specifically, we fit beta distributions for:

1. The 2-year and 5-year probabilities of developing ESRD as functions of age, sex, eGFR and UACR.
2. The 1, 2, 3, 4, 5, and 10 year probabilities of mortality for patients with CKD as functions of age, sex, race, eGFR, and UACR.
3. The proportion of patients with new-onset ESRD who start hemodialysis with a dialysis catheter.
4. The 1, 2, and 3 year probabilities of mortality for patients with ESRD (patients starting hemodialysis with a tunneled catheter and all other patients with ESRD) as functions of age, sex, and race.
5. For patients undergoing hemodialysis with a tunneled catheter, the 1, 2, and 3 year probabilities of transitioning off catheter as functions of age, sex, and race. We defined ESRD without a tunneled catheter as undergoing hemodialysis without a tunneled catheter, receiving peritoneal dialysis, or having a functional transplant.

For each of these distributions, we took 250 random and independent draws. Because these draws ignored inherent correlations in the data (e.g., older patients, all else being equal, die at a higher rate than younger patients), we used a method described by Goldhaber-Fiebert *et al*. to induce correlations.[15] This preserved the marginal distributions of each draw but ensured that logical inconsistencies did not arise in each scenario. We then computed a vector of transition probabilities using the constrained Nelder-Mead algorithm described earlier using each of these draws. This process yielded a joint distribution for the usual care transition probabilities.

To obtain the distribution for the MDC transition probabilities, we fit the odds ratios for death and progression to ESRD to lognormal distributions, using the published 95% confidence intervals.[6] We took 5000 random samples from each distribution and randomly assigned 20 samples to each of the 250 usual care vectors. We calibrated our model to each of these 5000 samples, which yielded the distribution of MDC transition probability vectors.

For each probability draw, we modified the MDC transition probabilities based on the assumed effectiveness of MDC on different CKD stages. To do this, we defined $\delta$ as above to achieve discounted transition probabilities. The result was a joint distribution for MDC transition probabilities for each of the defined effectiveness sensitivity analyses.

We fit QALYs and costs to beta and gamma distributions respectively using the derived 95% confidence intervals and took 5000 random samples from each. For QALYs, we induced correlations to ensure that patients with CKD had a higher quality of life than ESRD.

For each of the 5000 draws of transition probabilities, costs, and QALYs, we computed all outcomes, including total costs, change in costs, total QALYs, change in QALYs, and hazard ratios for death and ESRD. Because negative changes in costs and QALYs can be difficult to interpret in terms of ICERs, we instead calculated the net monetary benefit (NMB) for each probability draw at different WTP thresholds. Numerically, we computed:

$$NMB_{\lambda,i}=\left( Change in QALYs_{i} \right)*\lambda-\left( Change in cost_{i} \right)$$

where $\lambda$ is a specific WTP threshold and i is a specific probability draw. A positive $NMB_{\lambda,i}$ indicates that MDC is cost-effective for a WTP threshold of $\lambda$ and probability draw i. For each WTP threshold, we determined the proportion of probability draws that were cost-effective and used these to generate cost-effectiveness acceptability curves.

We also computed 95% confidence intervals for the hazard ratios for mortality and progression to ESRD using the joint distribution of transition probabilities. We used the lower and upper 2.5% quantiles to generate the confidence intervals for each estimate.

To obtain estimates for population aggregates (e.g., all females or all patients 45-64 years old), for each of the 5000 samples, we took the weighted average of the above outcomes, using population counts by CKD stage, age, sex, and race. For ICERs and NMBs, we first took the weighted average of the change in costs and change in QALYs, then computed the respective statistics.

# S1 APPENDIX REFERENCES

1. Nelder JA, Mead R. A Simplex Method for Function Minimization. Comput J. 1965;7: 308–313. doi:10.1093/comjnl/7.4.308

2. Tangri N, Grams ME, Levey AS, Coresh J, Appel LJ, Astor BC, et al. Multinational Assessment of Accuracy of Equations for Predicting Risk of Kidney Failure: A Meta-analysis. JAMA. 2016;315: 164–174. doi:10.1001/jama.2015.18202

3. Arias E. United States life tables, 2011. Natl Vital Stat Rep. 2015;64. Available: https://www.cdc.gov/nchs/data/nvsr/nvsr64/nvsr64_11.pdf

4. van der Velde M, Matsushita K, Coresh J, Astor BC, Woodward M, Levey A, et al. Lower estimated glomerular filtration rate and higher albuminuria are associated with all-cause and cardiovascular mortality. A collaborative meta-analysis of high-risk population cohorts. Kidney Int. 2011;79: 1341–1352. doi:10.1038/ki.2010.536

5. United States Renal Data System. 2015 USRDS Annual Data Report: Epidemiology of kidney disease in the United States. Bethesda, MD: National Institutes of Health, National Institute of Diabetes and Digestive and Kidney Diseases; 2015.

6. Wang S-M, Hsiao L-C, Ting I-W, Yu T-M, Liang C-C, Kuo H-L, et al. Multidisciplinary care in patients with chronic kidney disease: A systematic review and meta-analysis. Eur J Intern Med. 2015; doi:10.1016/j.ejim.2015.07.002

7. Bureau of Labor and Statistics. CPI Inflation Calculator. Available: http://data.bls.gov/cgi-bin/cpicalc.pl

8. United States Renal Data System. Chapter 7: Medicare Part D Prescription Drug Coverage in Patinets with CKD. 2016 USRDS annual data report: Epidemiology of kidney disease in the United States. Bethesda, MD: National Institutes of Health, National Institute of Diabetes and Digestive and Kidney Diseases; 2016.

9. United States Renal Data System. Chapter 12: Medicare Part D Prescription Drug Coverage in Patients with ESRD. 2016 USRDS annual data report: Epidemiology of kidney disease in the United States. Bethesda, MD: National Institutes of Health, National Institute of Diabetes and Digestive and Kidney Diseases; 2016.

10. Department of Health and Human Services, Centers for Medicare & Medicaid Services. Medicare Program; Revisions to Payment Policies Under the Physician Fee Schedule and Other Revisions to Part B for CY 2017; Medicare Advantage Bid Pricing Data Release; Medicare Advantage and Part D Medical Loss Ratio Data Release; Medicare Advantage Provider Network Requirements; Expansion of Medicare Diabetes Prevention Program Model; Medicare Shared Savings Program Requirements [Internet]. Nov 15, 2016 pp. 80170–80562. Available: https://www.gpo.gov/fdsys/pkg/FR-2016-11-15/pdf/2016-26668.pdf

11. Levin A, Lewis M, Mortiboy P, Faber S, Hare I, Porter EC, et al. Multidisciplinary predialysis programs: quantification and limitations of their impact on patient outcomes in two Canadian settings. Am J Kidney Dis Off J Natl Kidney Found. 1997;29: 533–540.

12. Wyld M, Morton RL, Hayen A, Howard K, Webster AC. A systematic review and meta-analysis of utility-based quality of life in chronic kidney disease treatments. PLoS Med. 2012;9: e1001307. doi:10.1371/journal.pmed.1001307

13. Gorodetskaya I, Zenios S, McCulloch CE, Bostrom A, Hsu C-Y, Bindman AB, et al. Health-related quality of life and estimates of utility in chronic kidney disease. Kidney Int. 2005;68: 2801–2808. doi:10.1111/j.1523-1755.2005.00752.x

14. Cruz MC, Andrade C, Urrutia M, Draibe S, Nogueira-Martins LA, Sesso R de CC. Quality of life in patients with chronic kidney disease. Clin São Paulo Braz. 2011;66: 991–995.

15. Goldhaber-Fiebert JD, Jalal HJ. Some Health States Are Better Than Others: Using Health State Rank Order to Improve Probabilistic Analyses. Med Decis Mak Int J Soc Med Decis Mak. 2016;36: 927–940. doi:10.1177/0272989X15605091
