# Supplementary figures and images for: Cost-effectiveness of multidisciplinary care in mild to moderate chronic kidney disease in the United States: A modeling study
Source: PLoS Med. 2018 Mar 27;15(3):e1002532. doi: 10.1371/journal.pmed.1002532 (PMC5870947; doi:10.1371/journal.pmed.1002532)

# S2 APPENDIX: CALIBRATION RESULTS

**
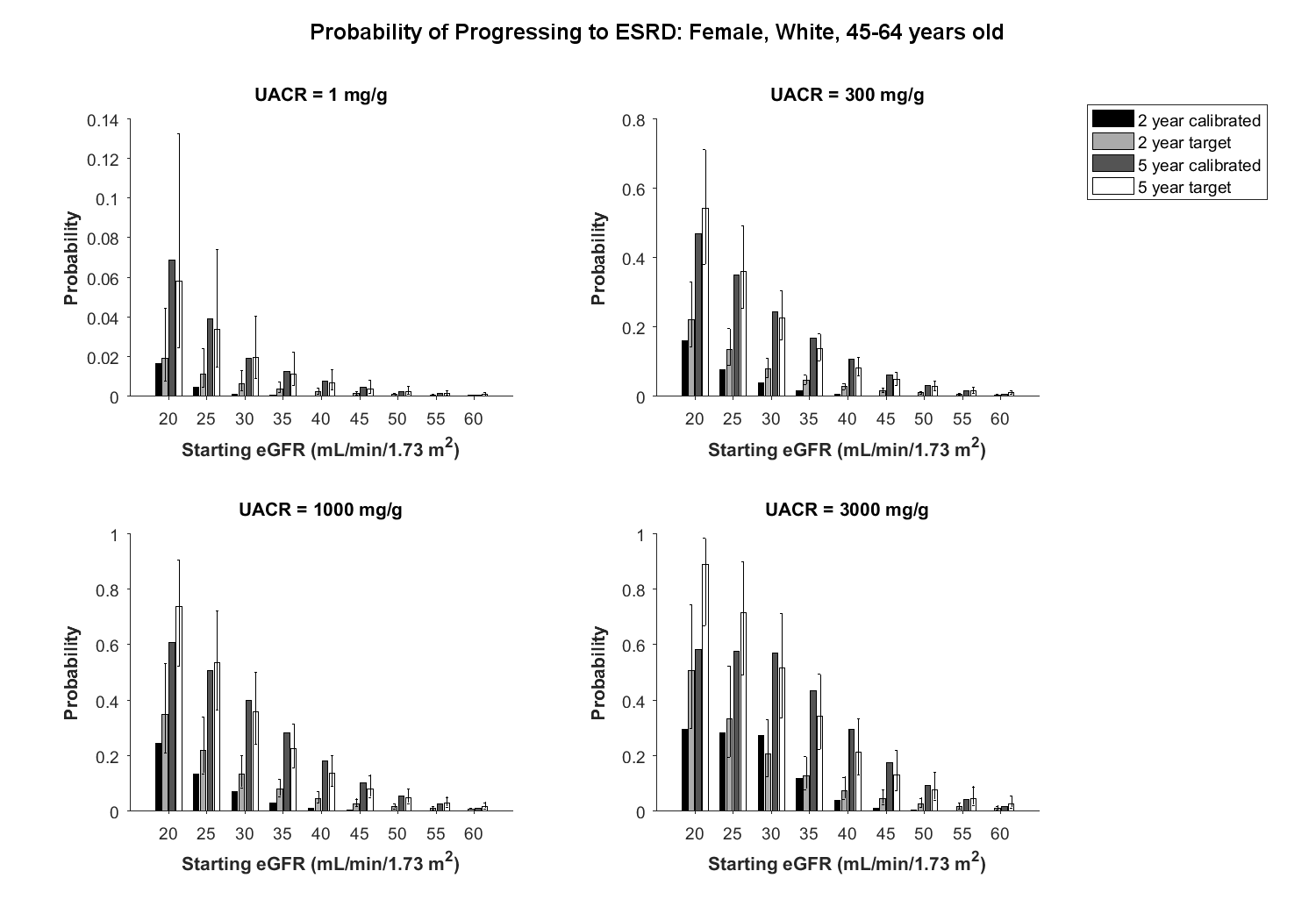
**

**
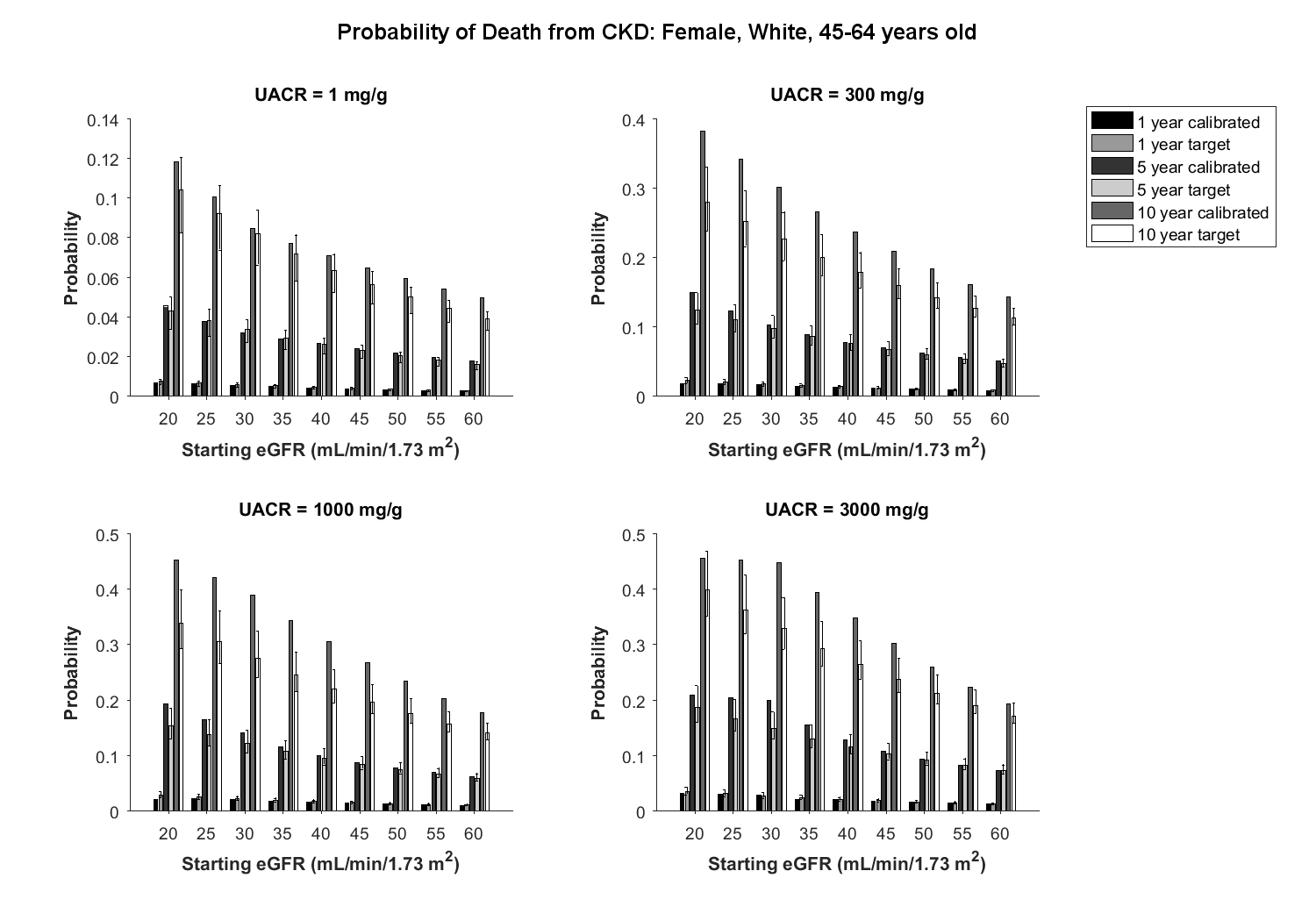
**

**
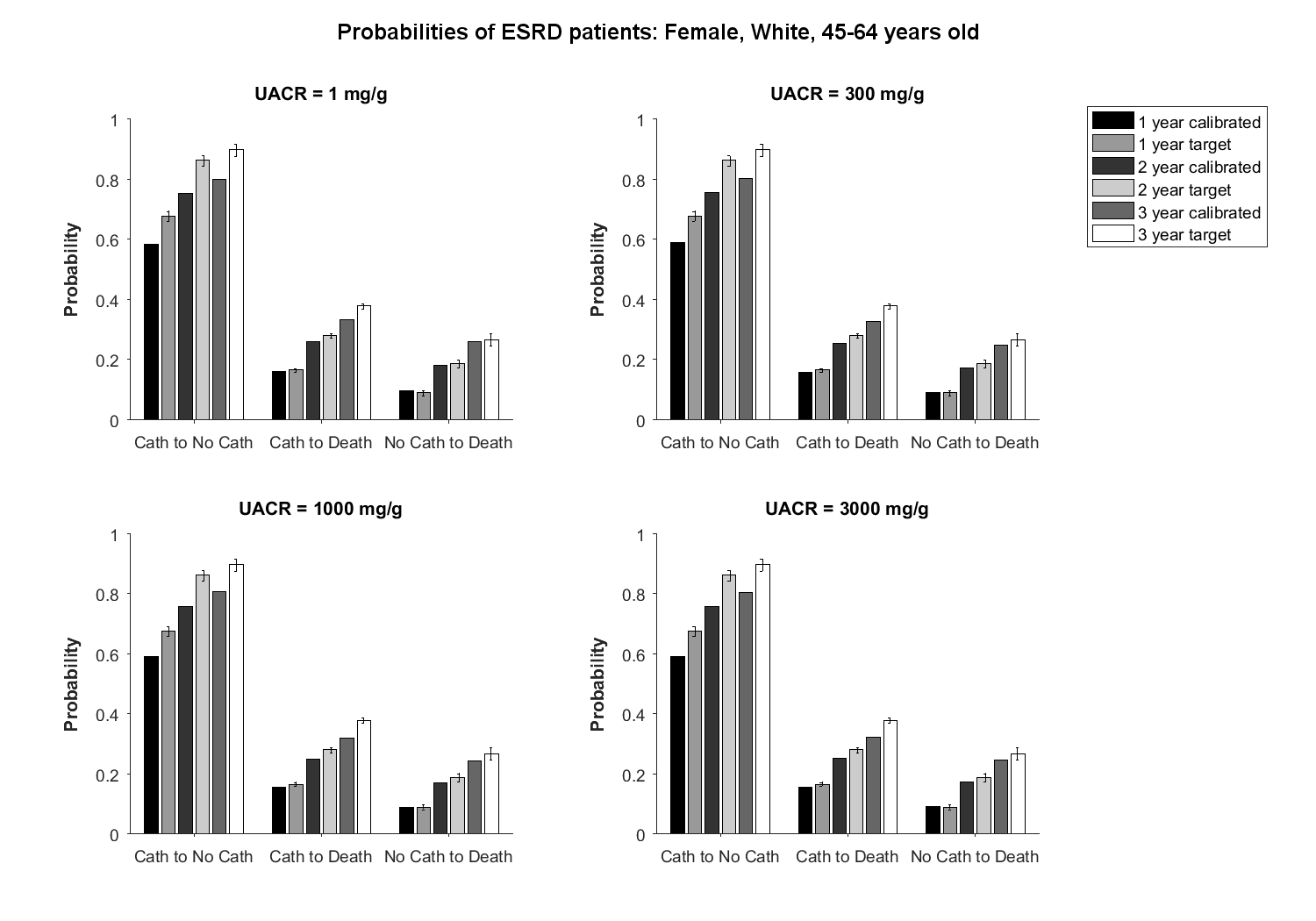
**

**
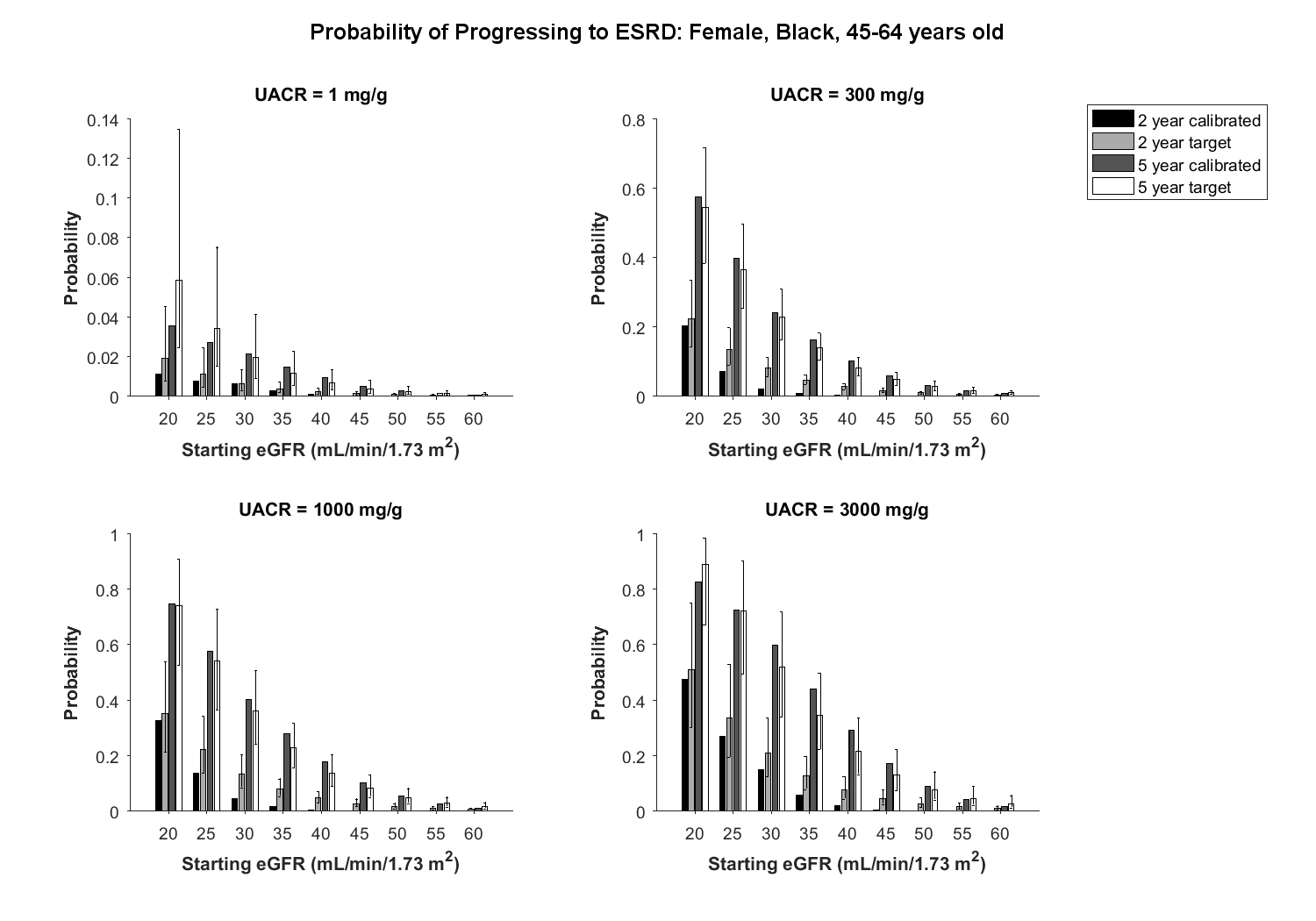
**

**
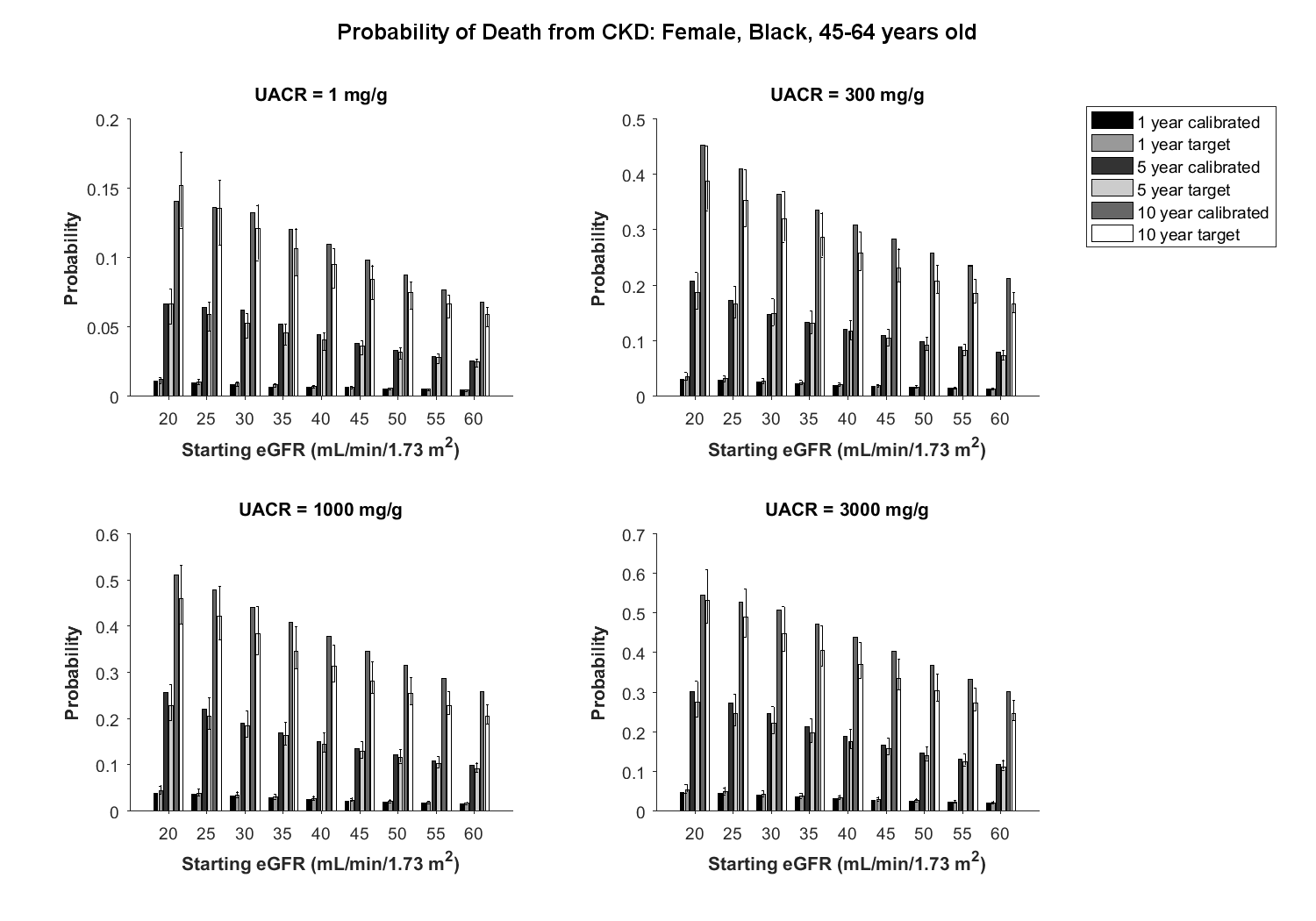
**

**
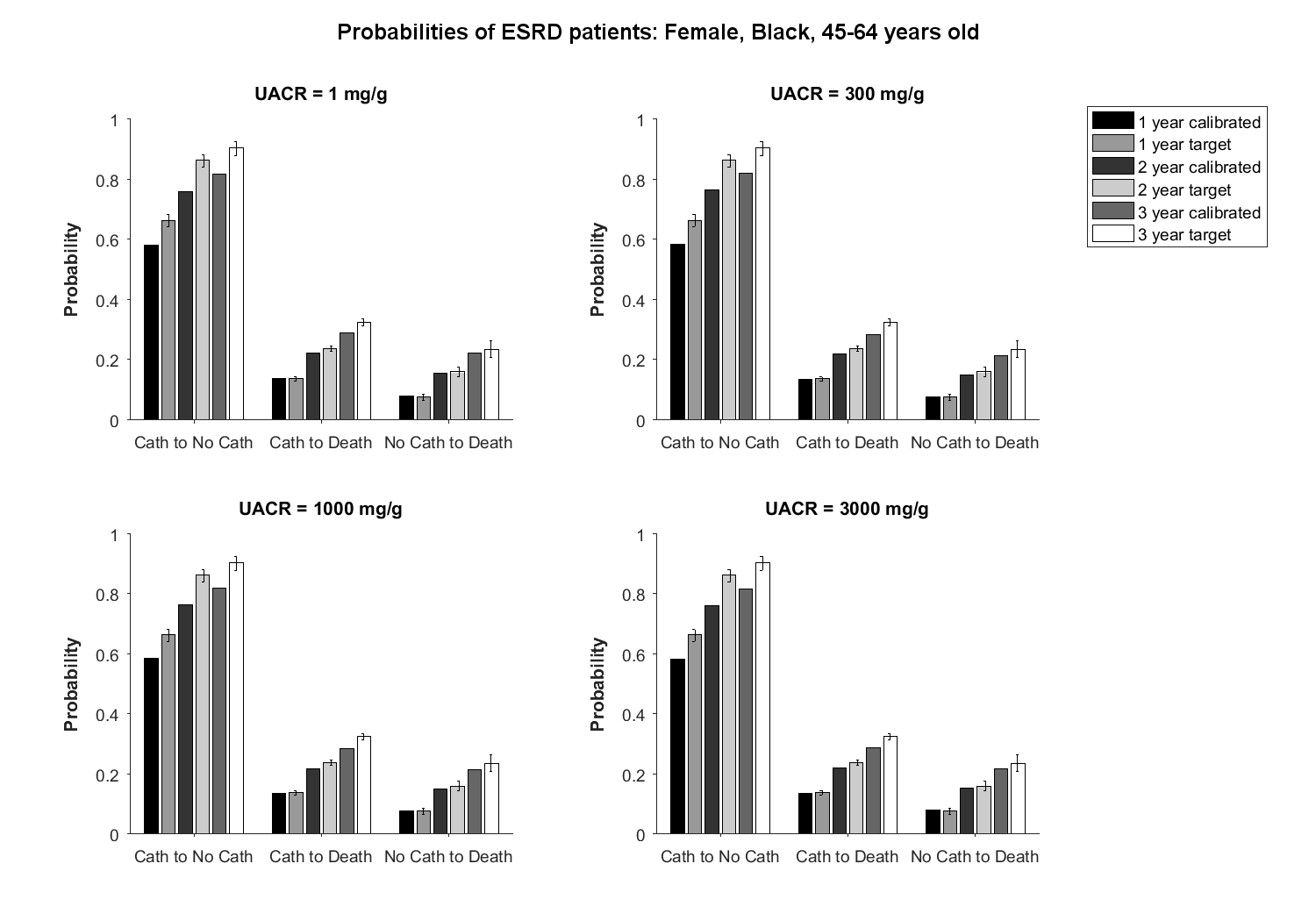
**

**
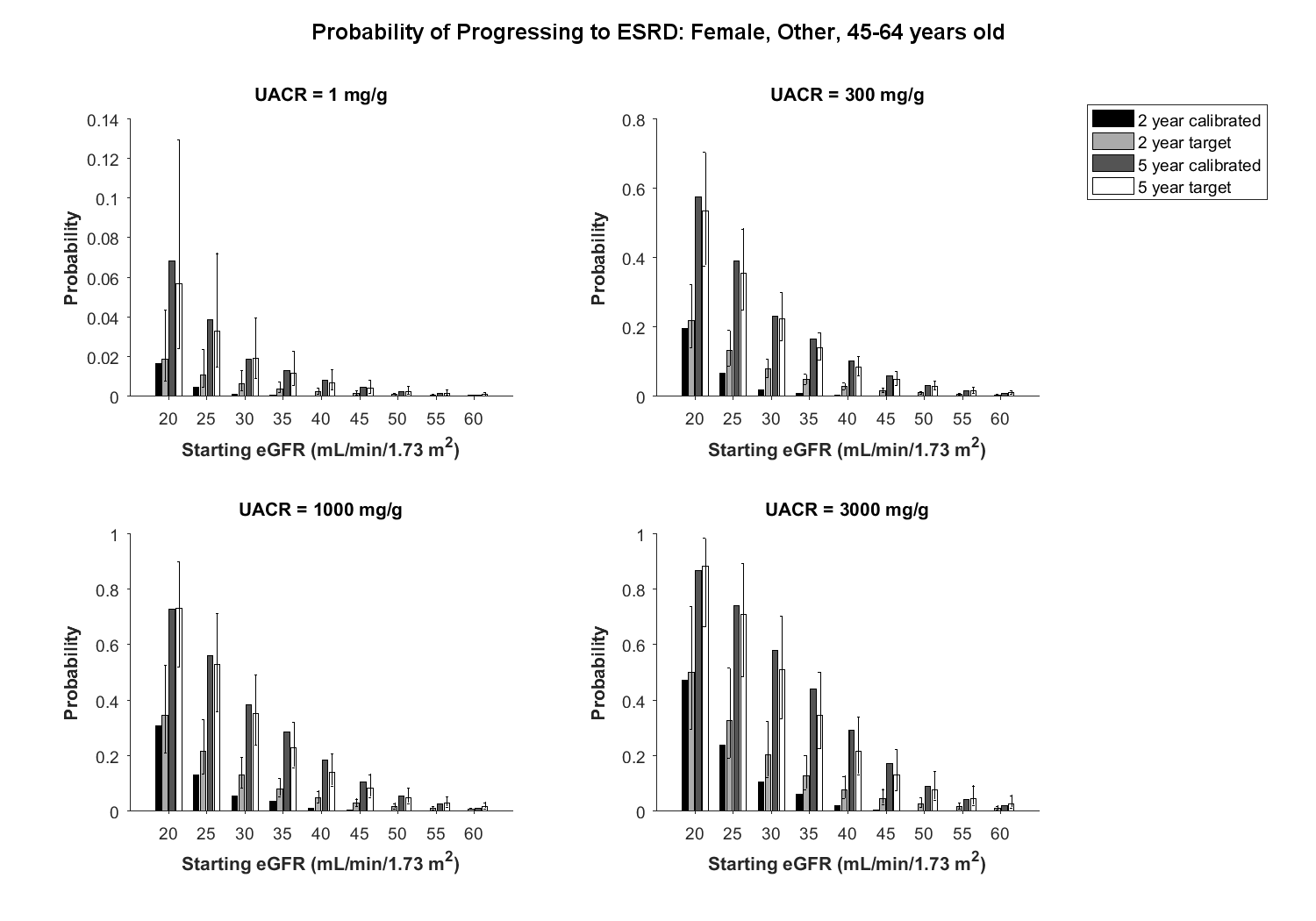
**

**
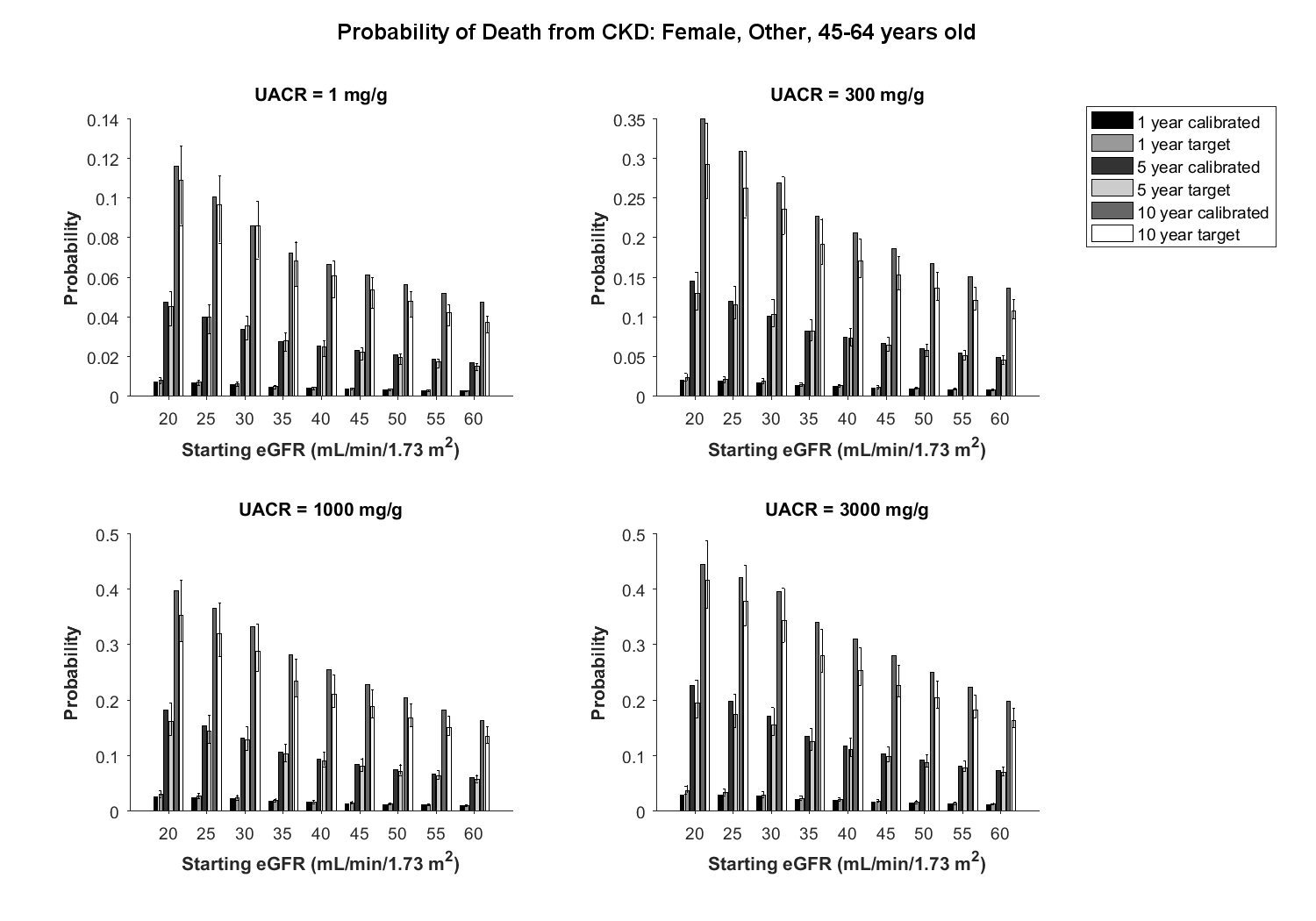
**

**
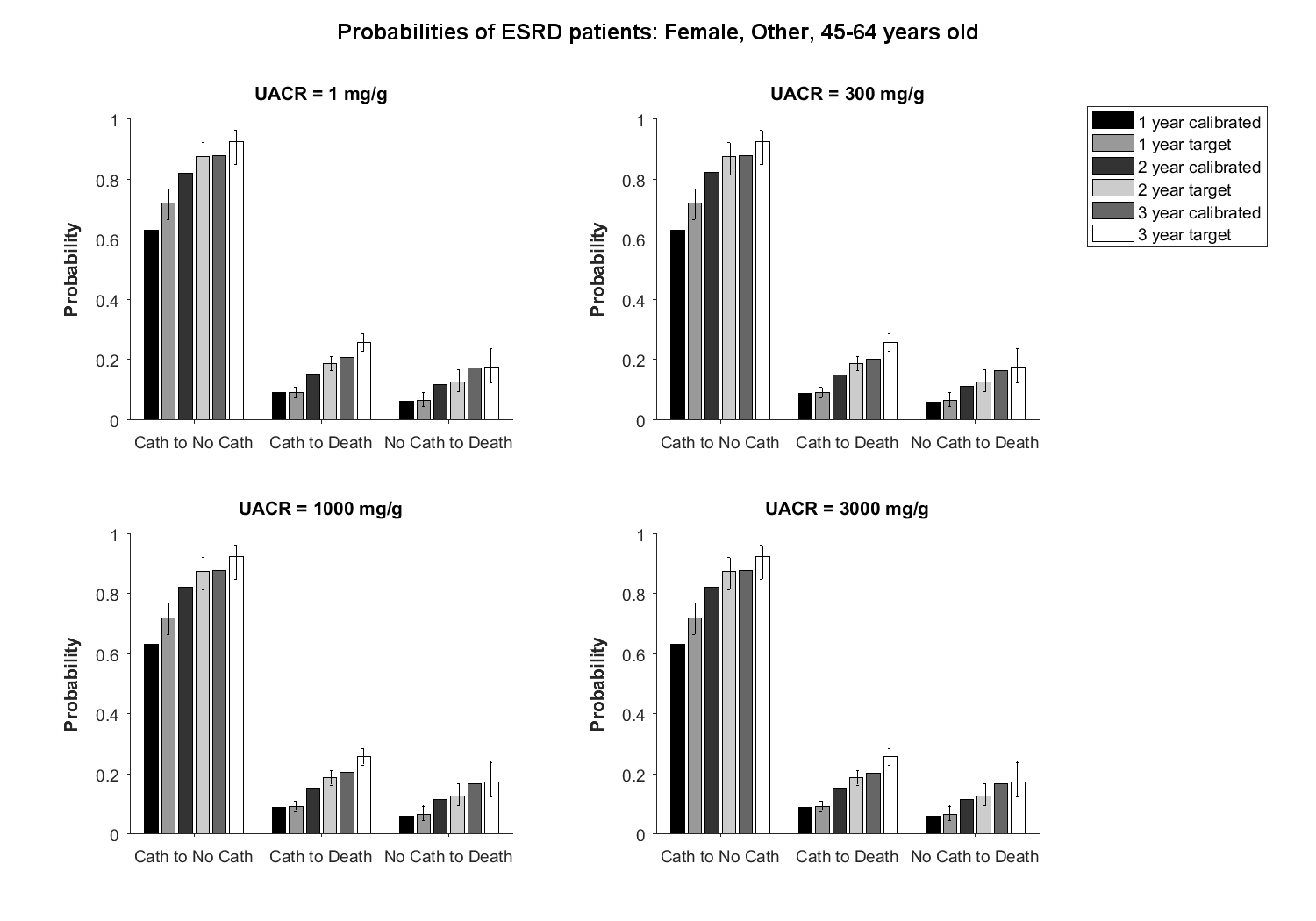
**

**
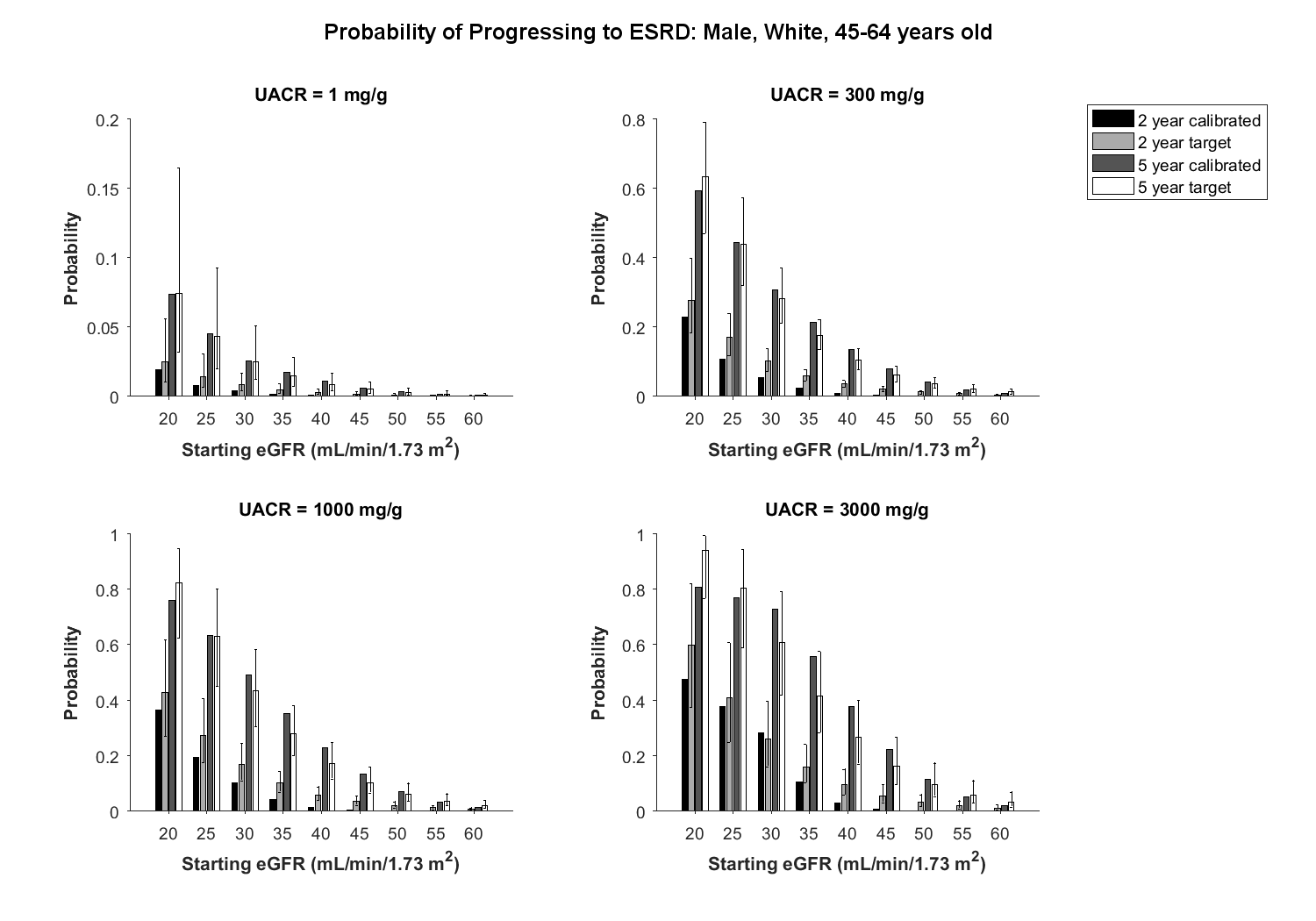
**

**
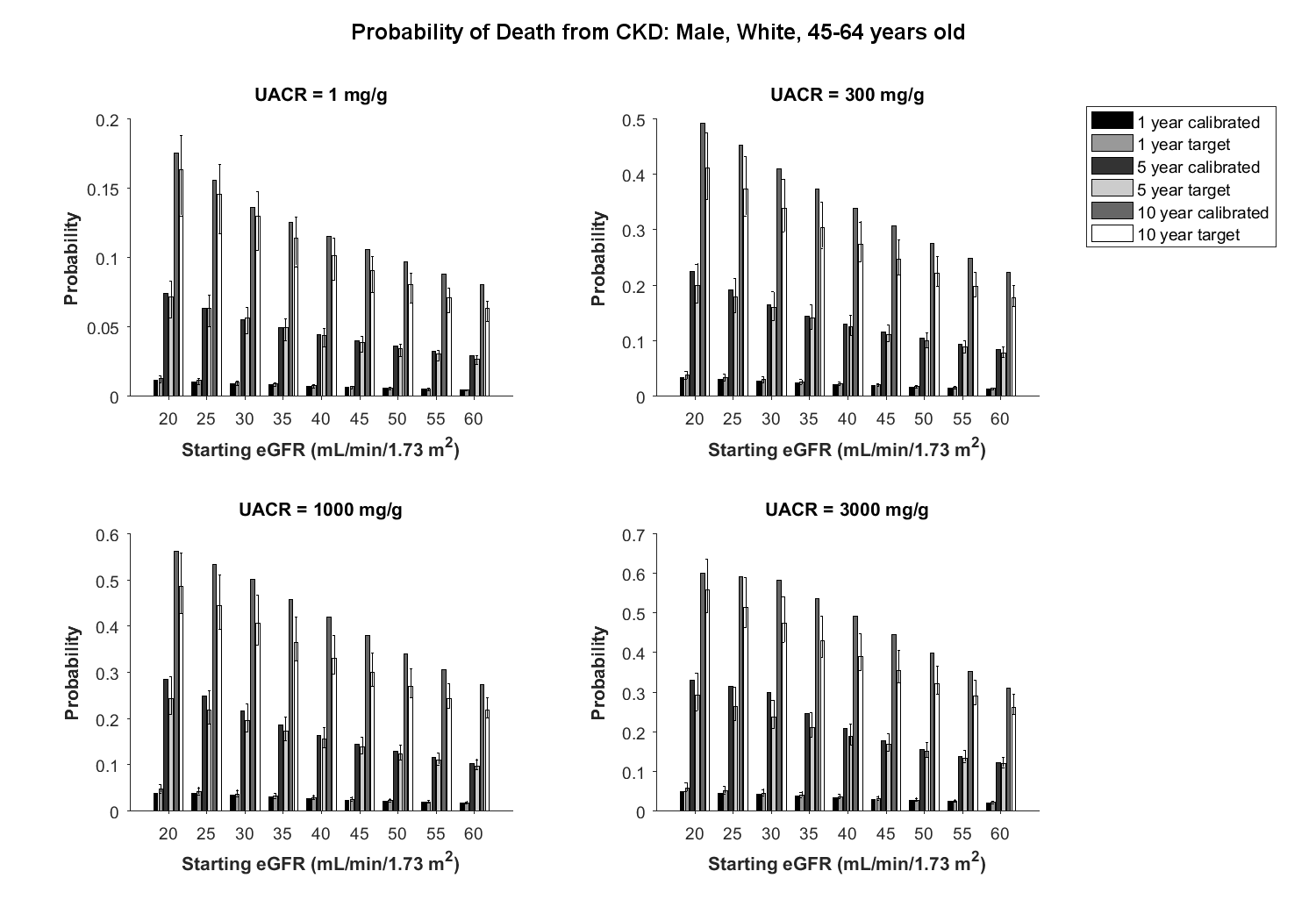
**

**
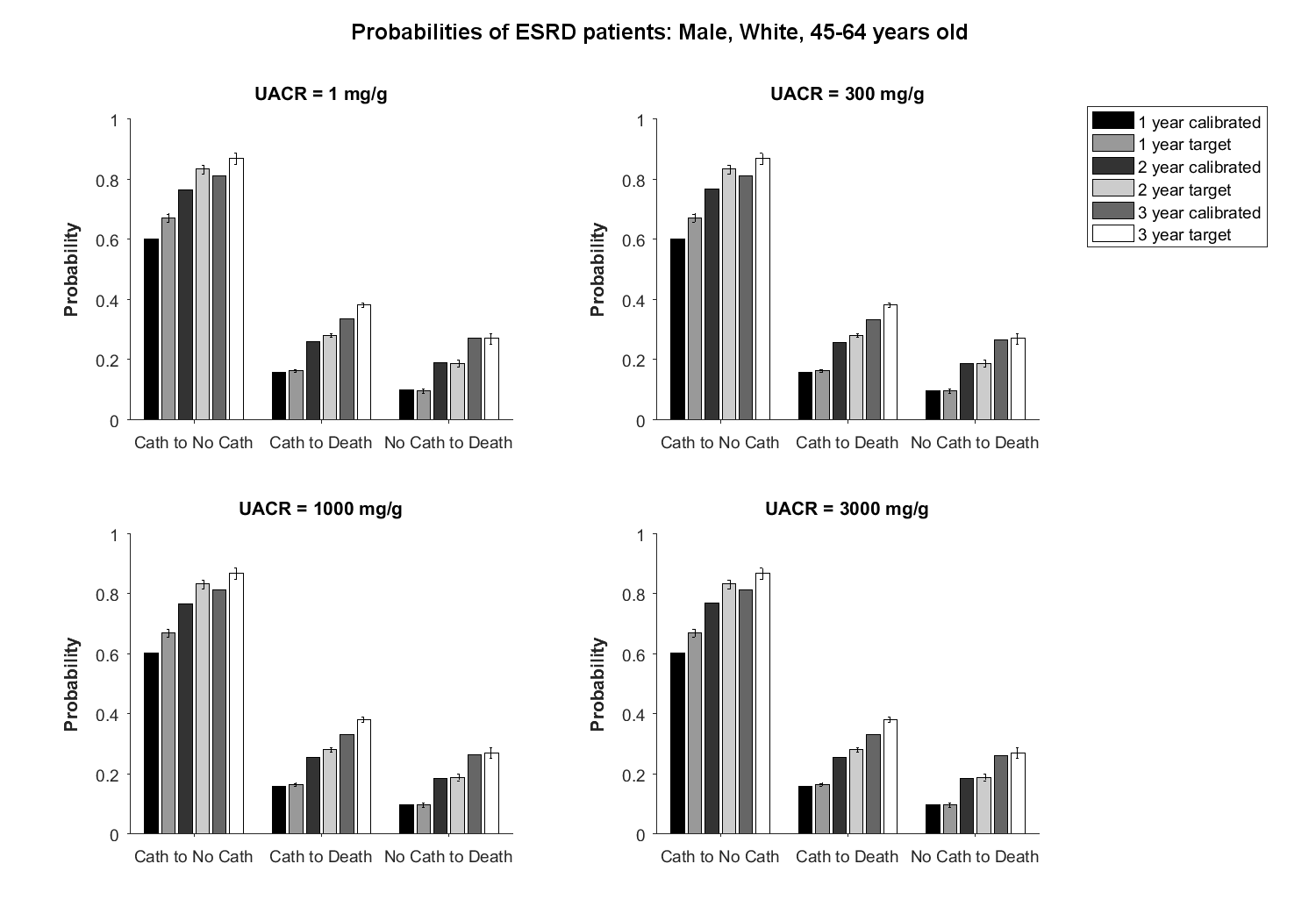
**

**
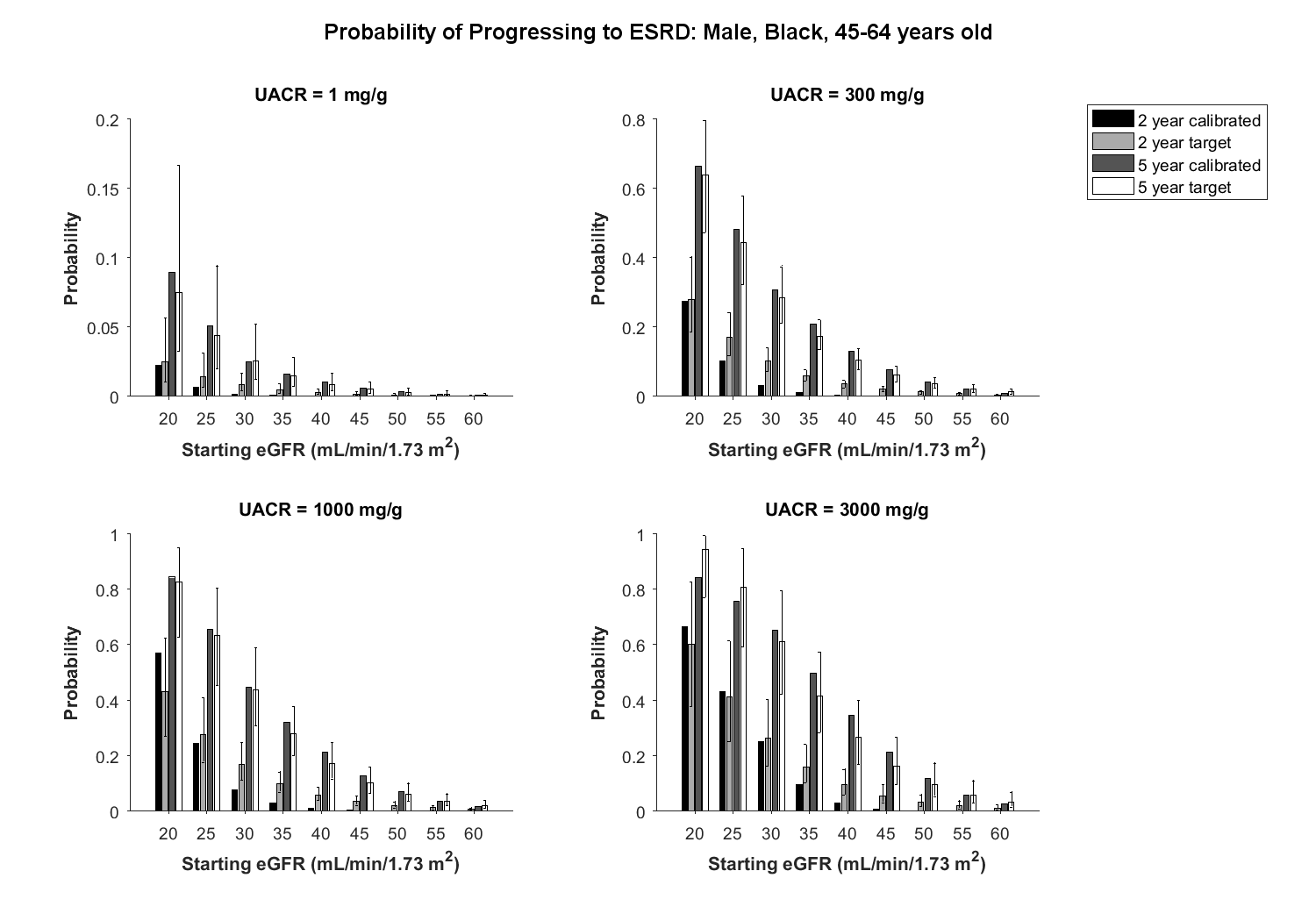
**

**
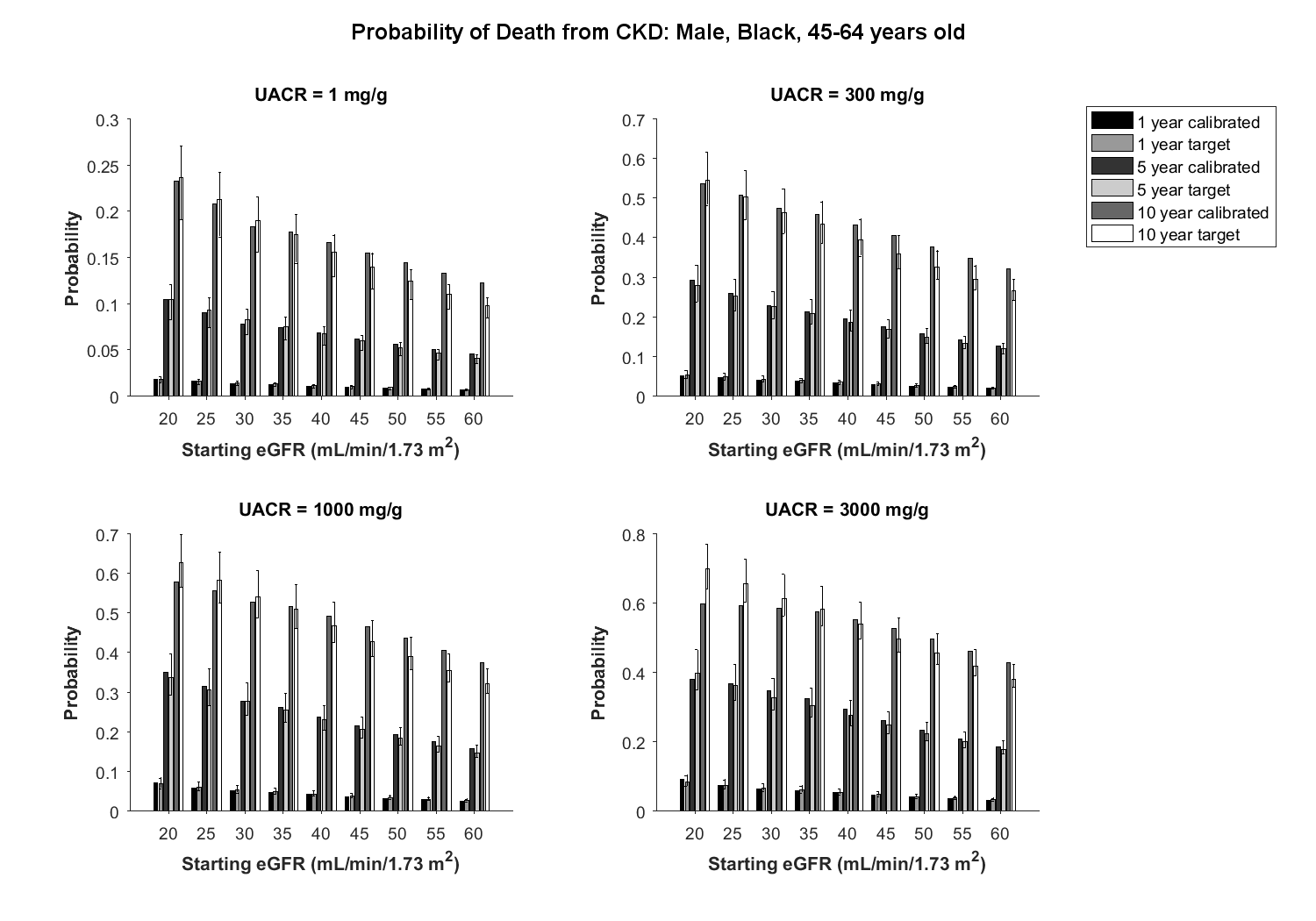
**

**
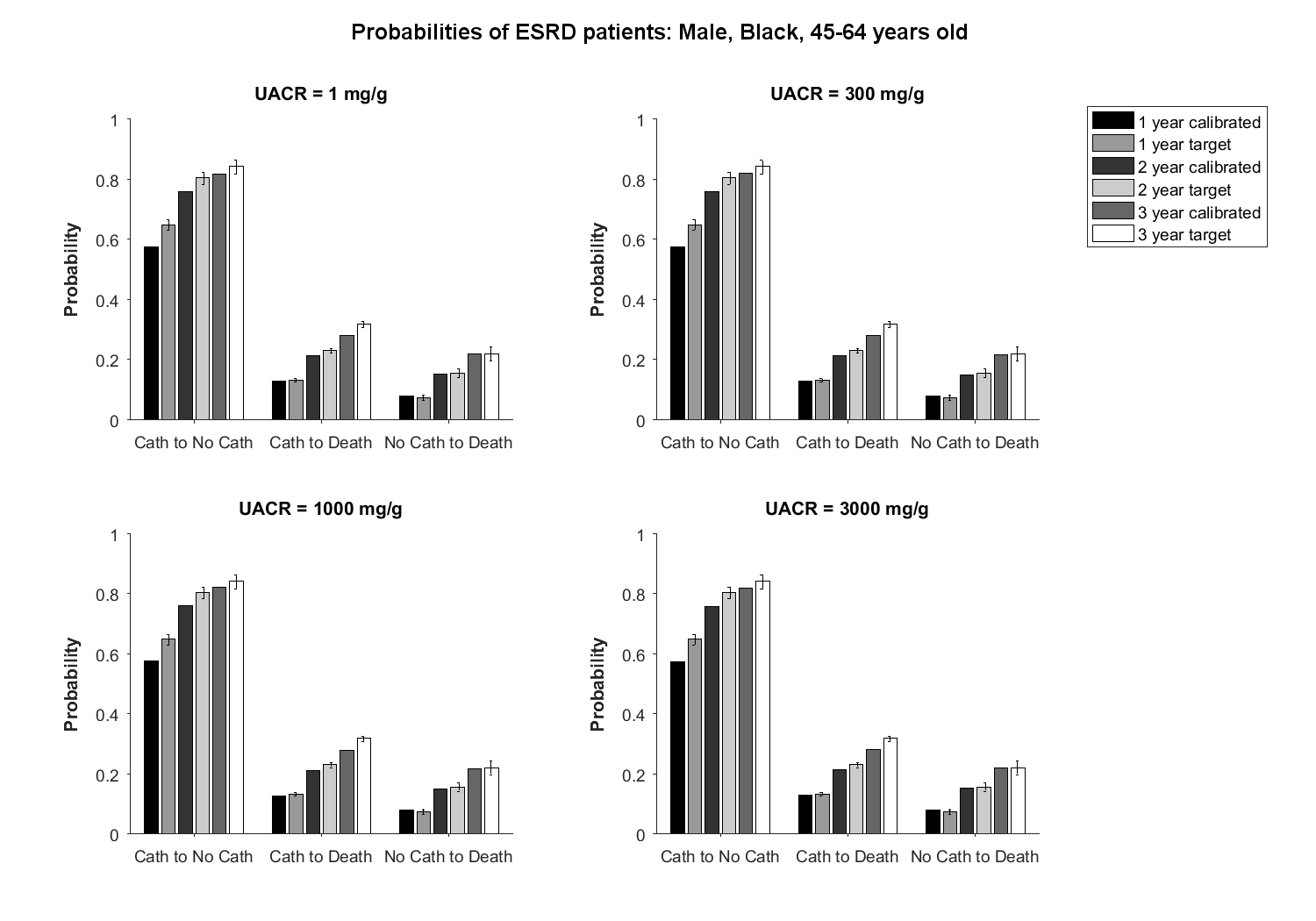
**

**
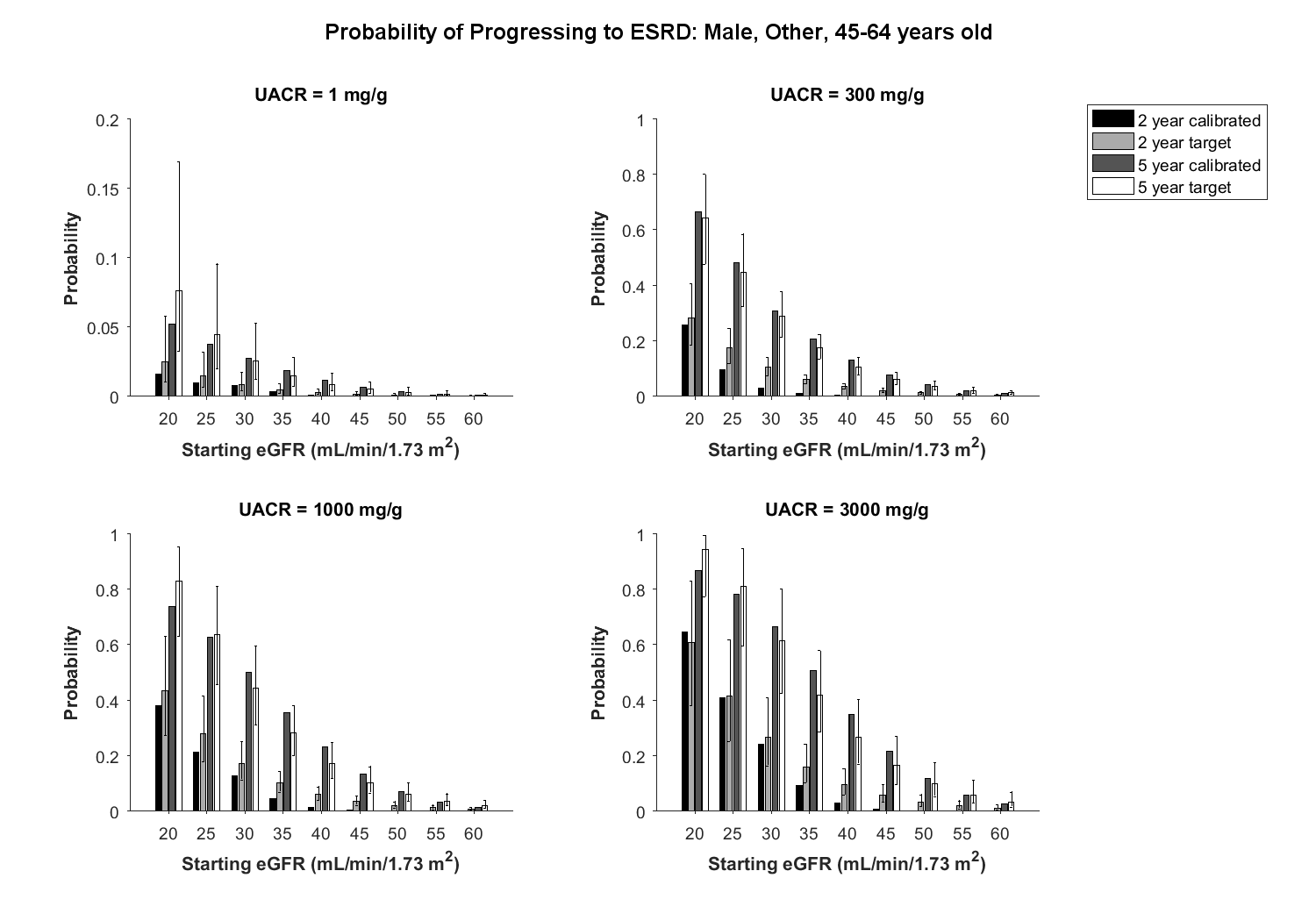
**

**
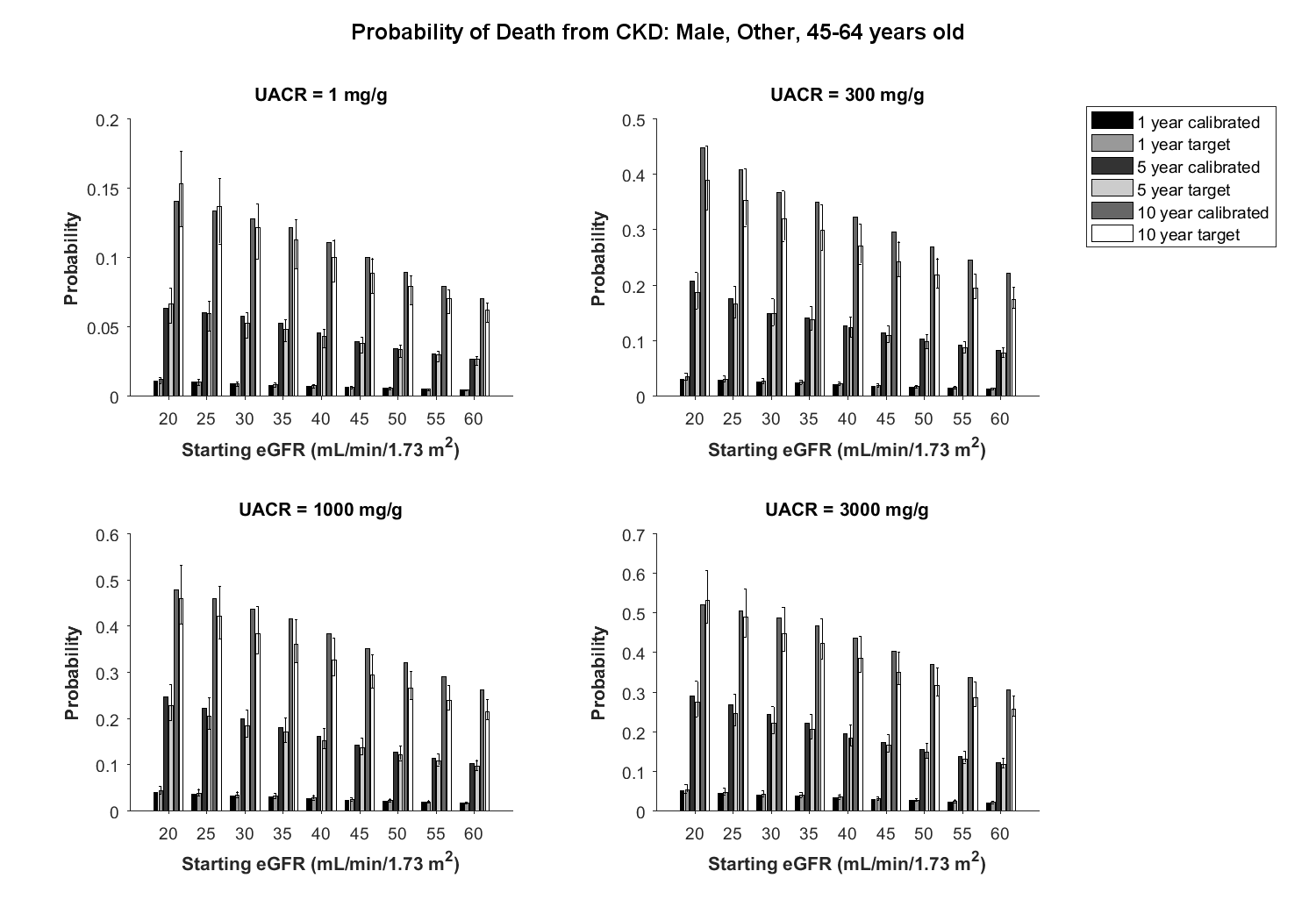
**

**
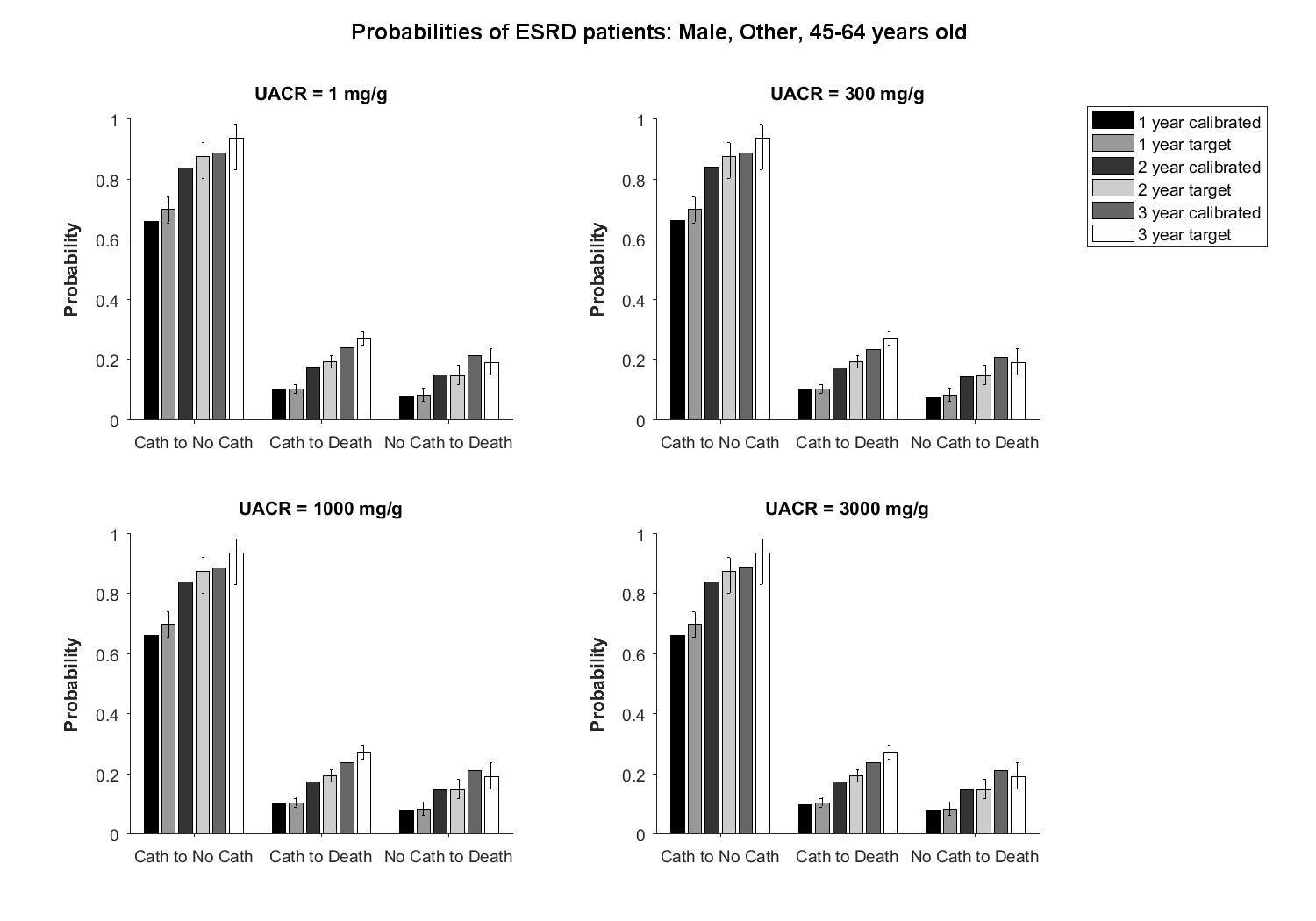
**

**
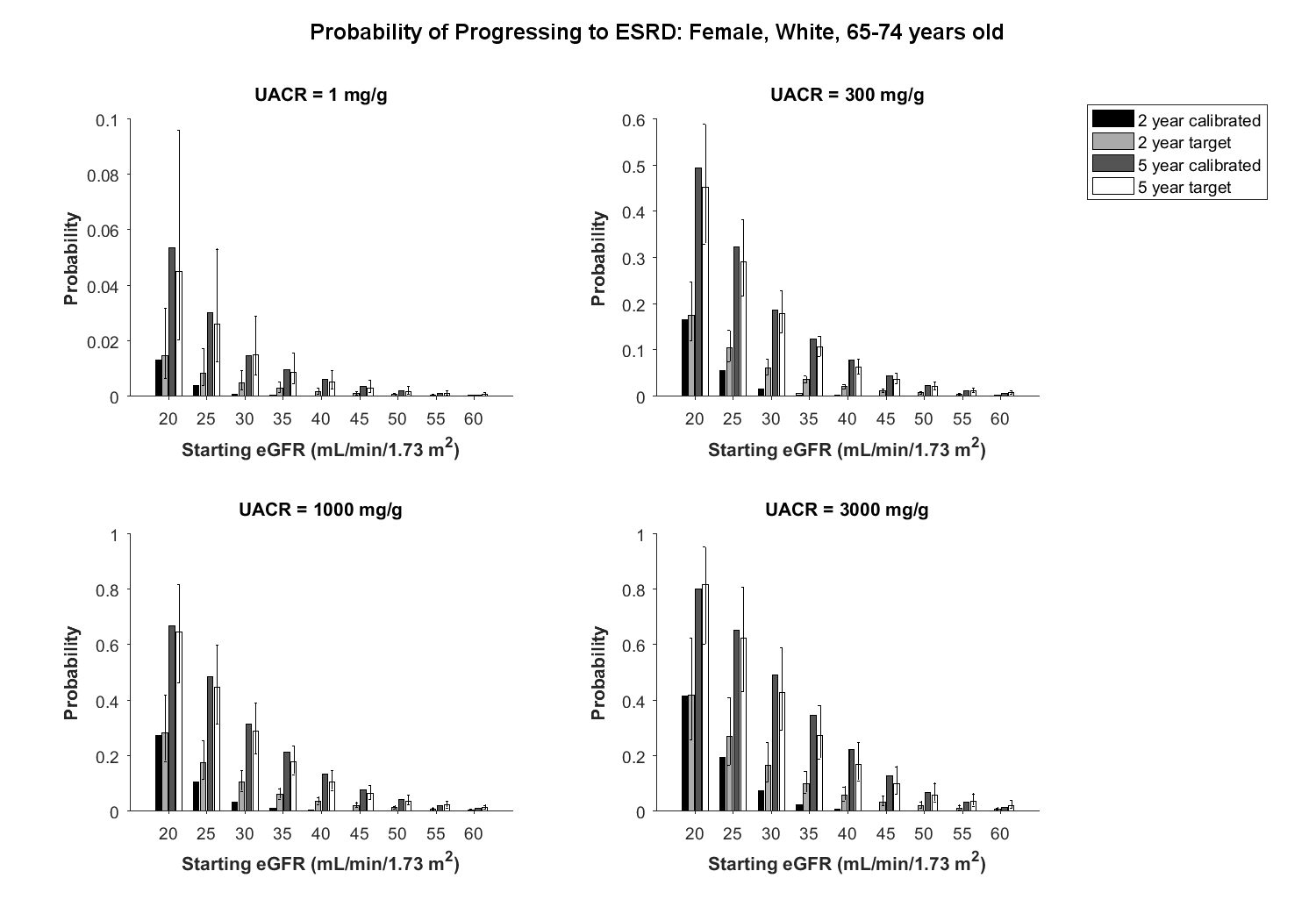
**


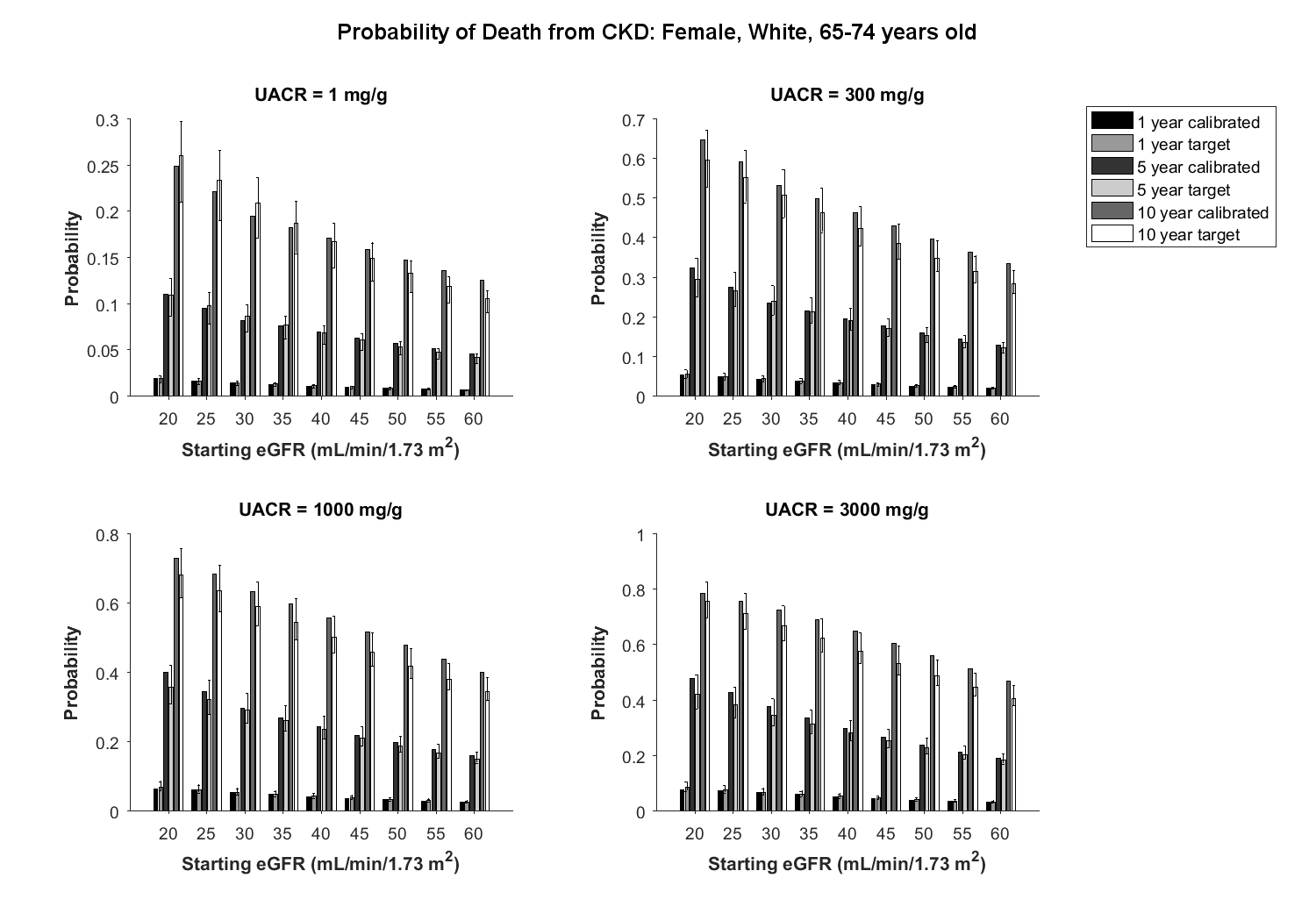


**
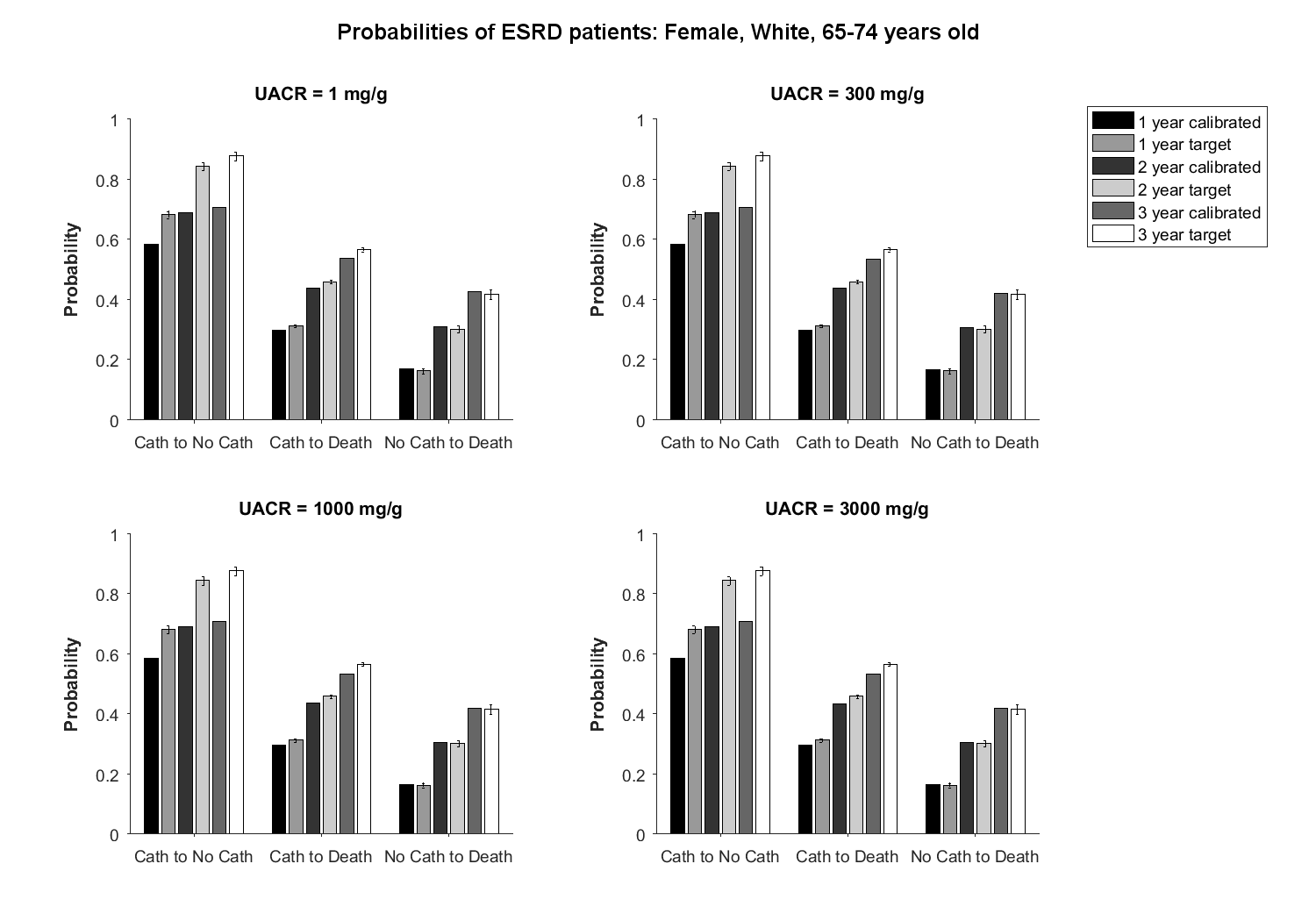
**

**
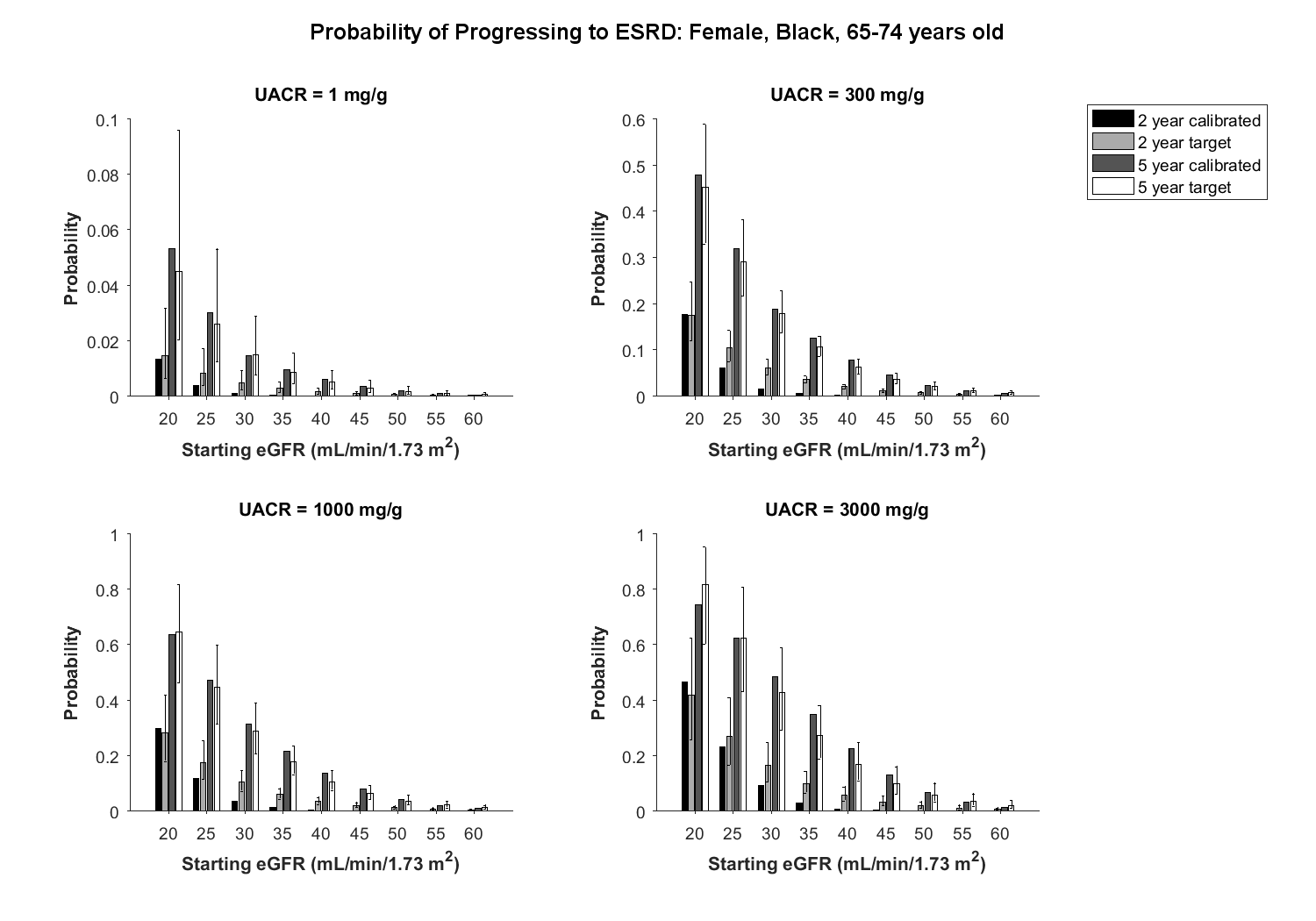
**

**
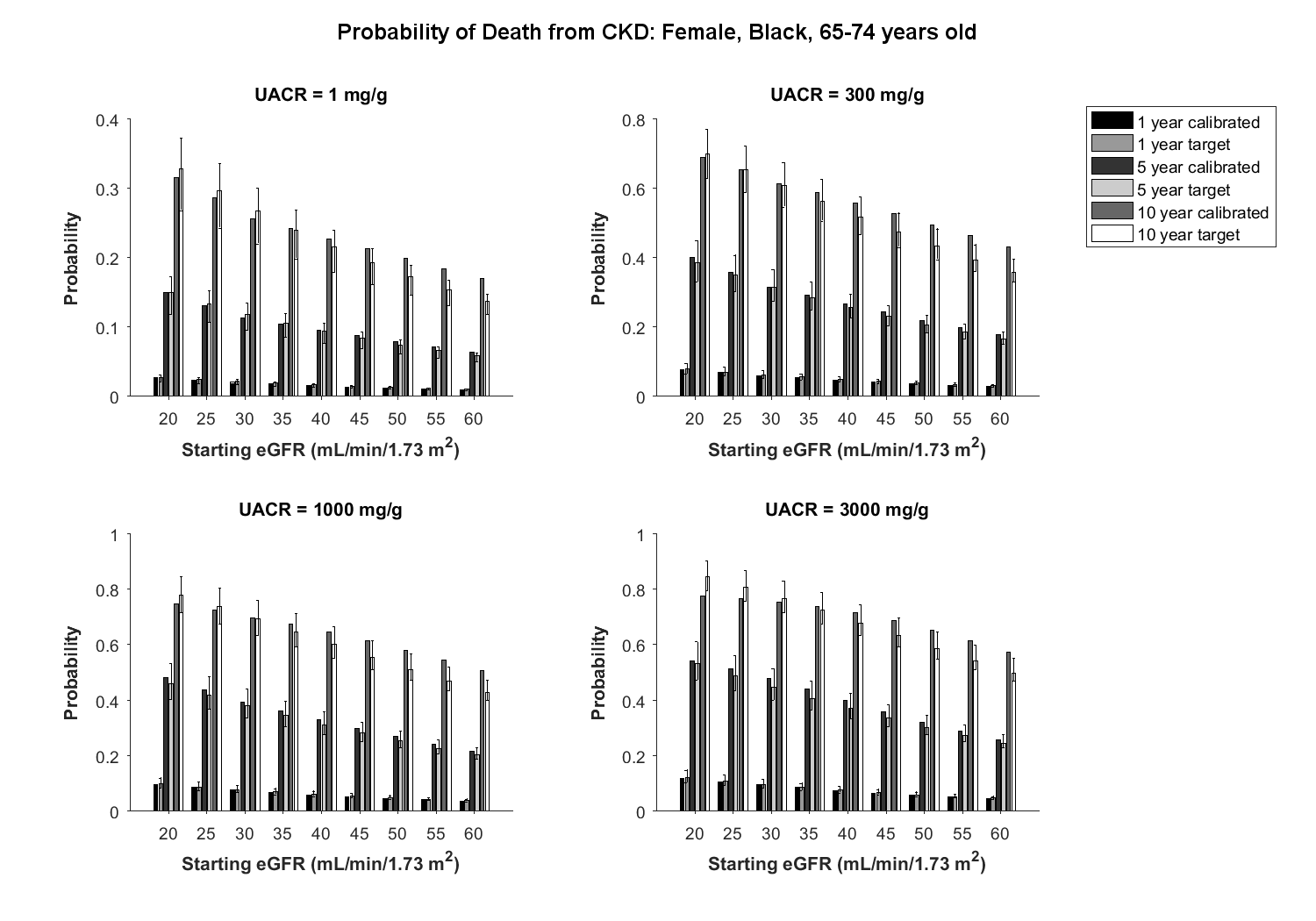
**

**
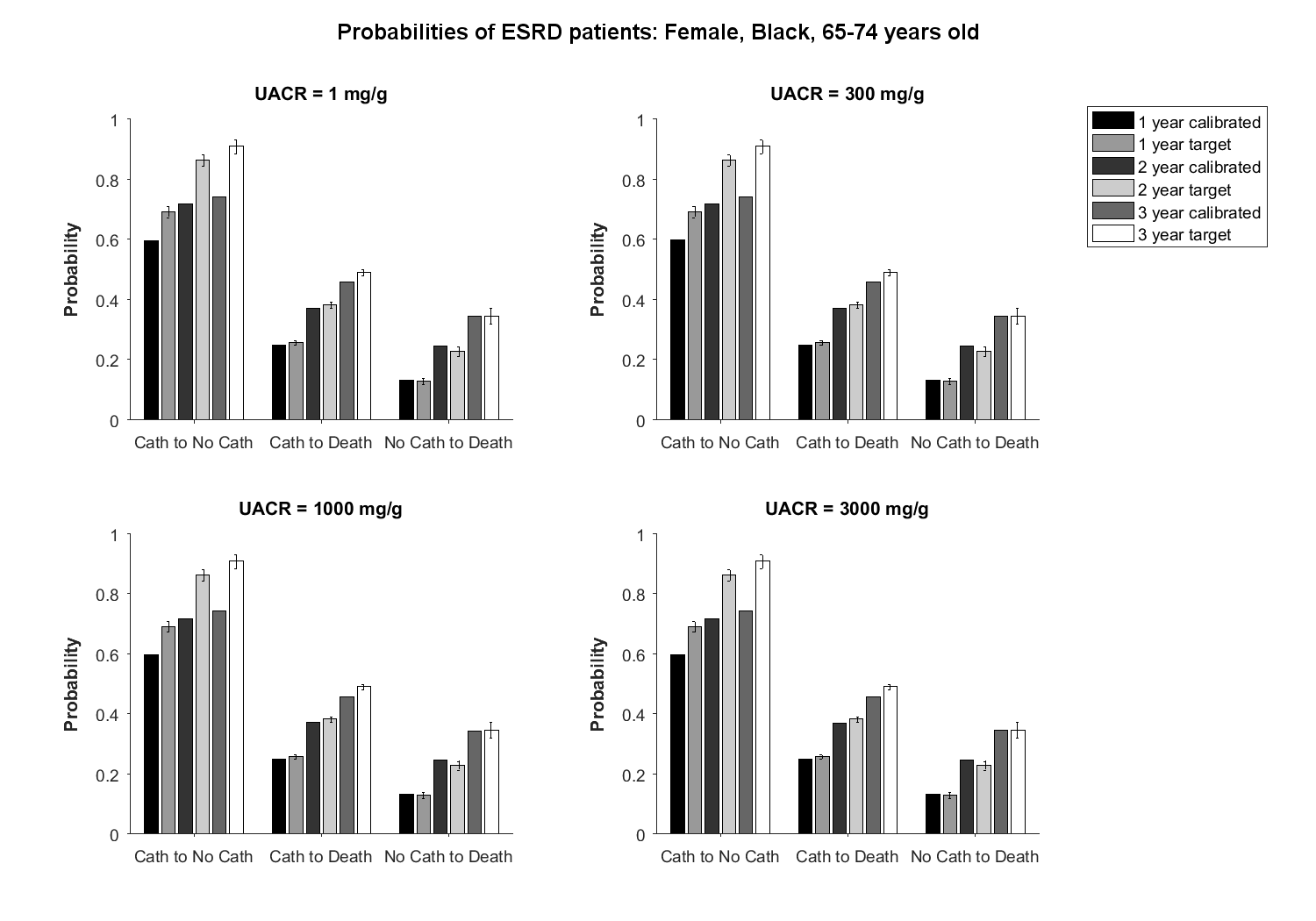
**

**
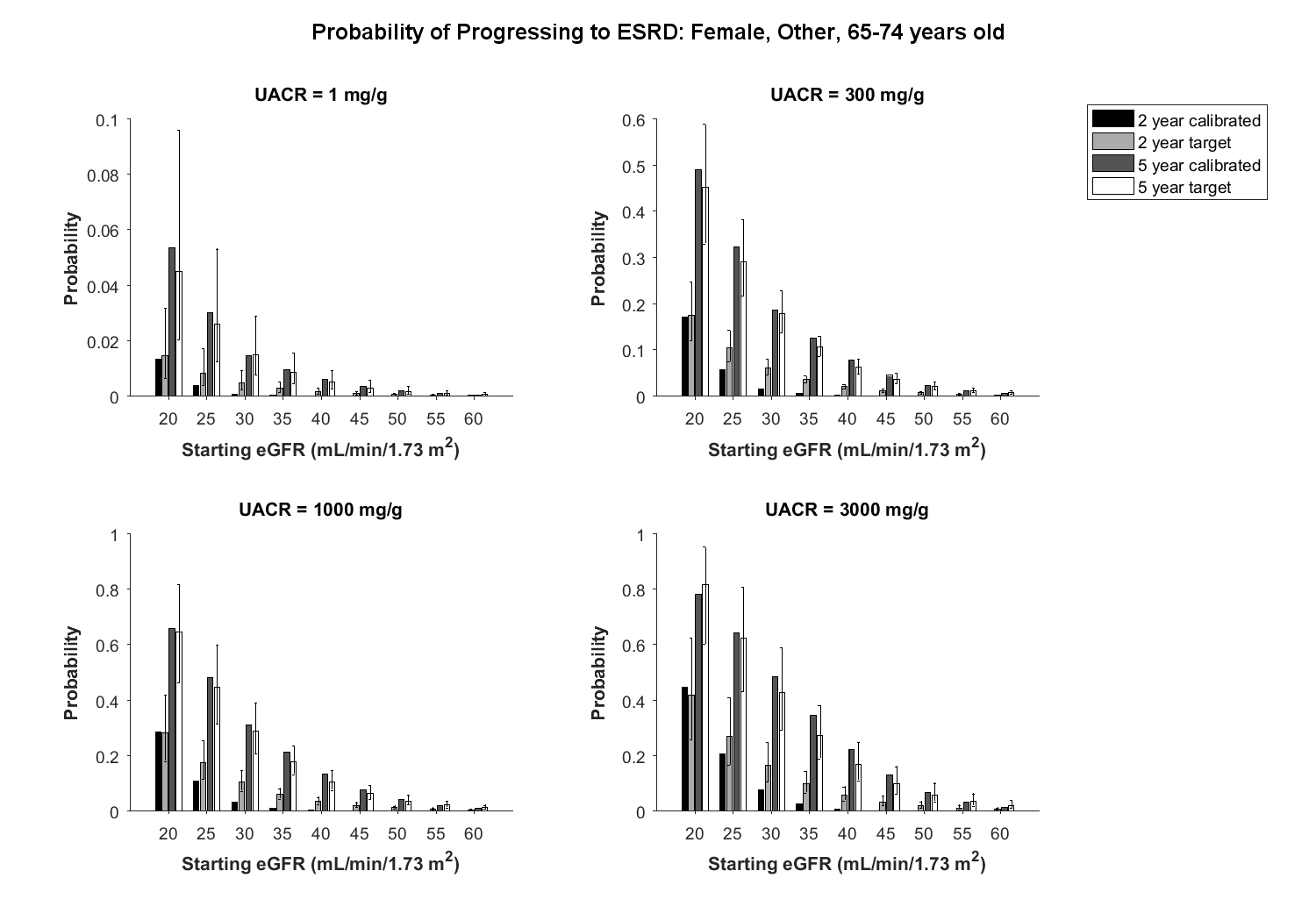
**

**
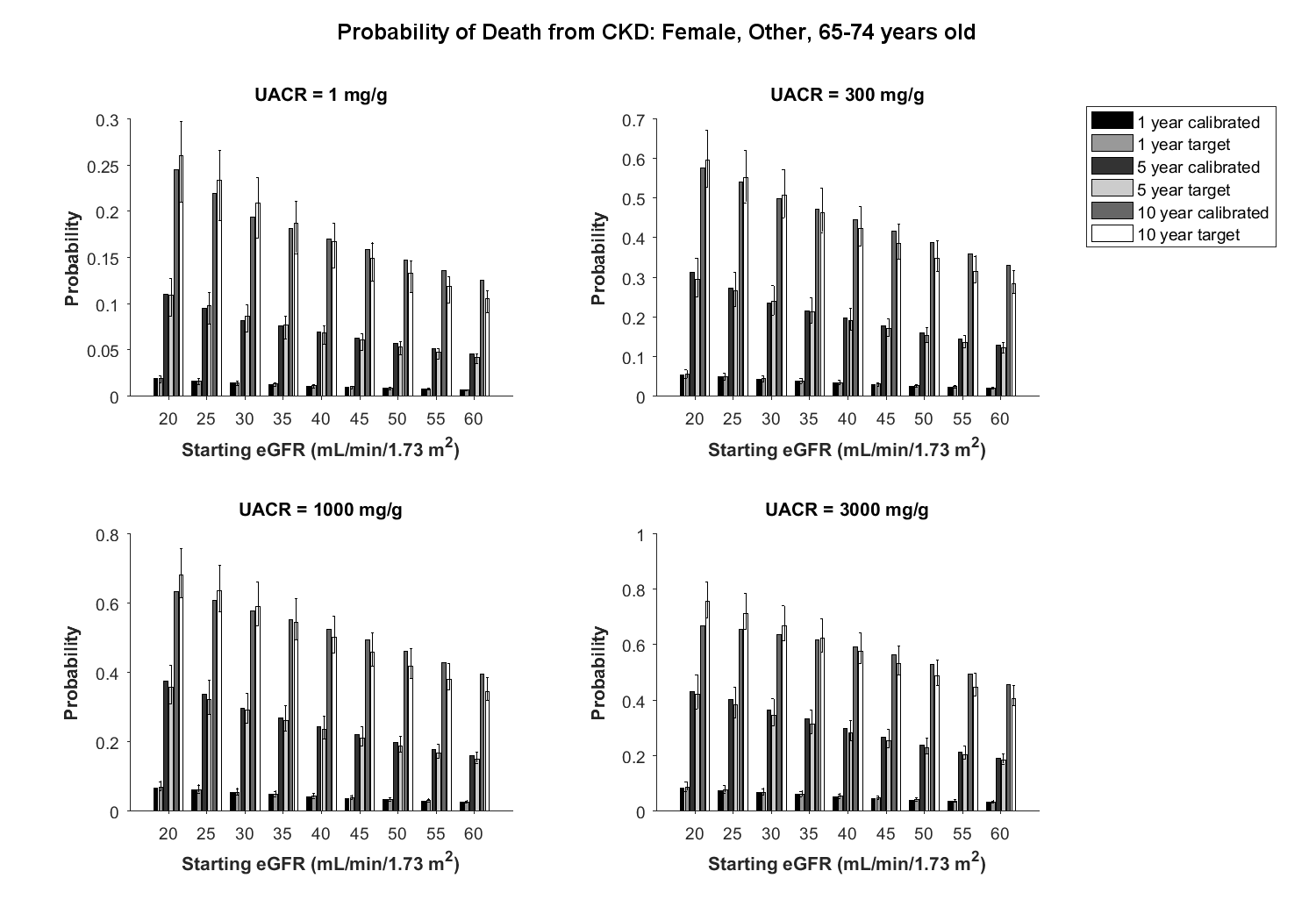
**

**
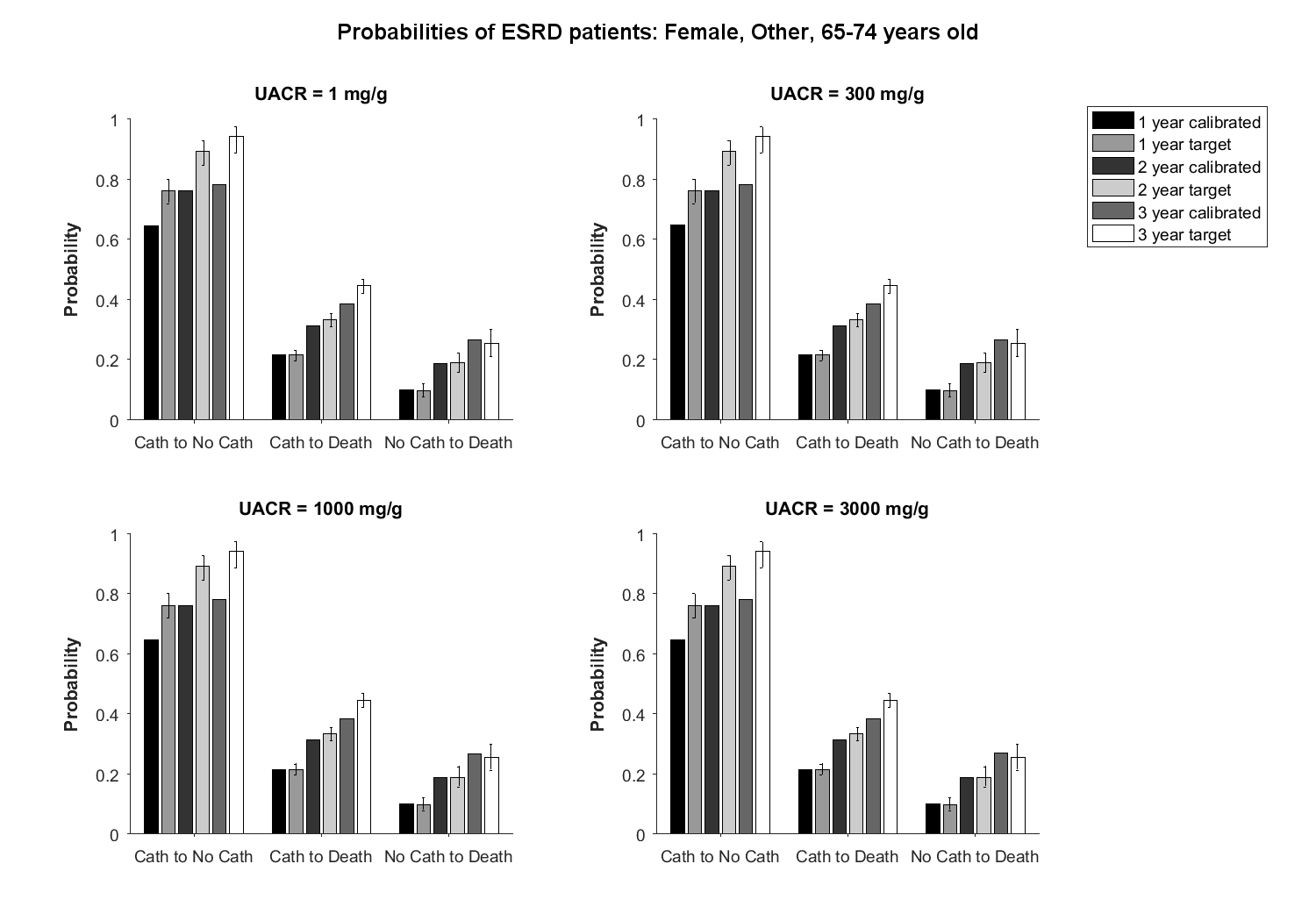
**

**
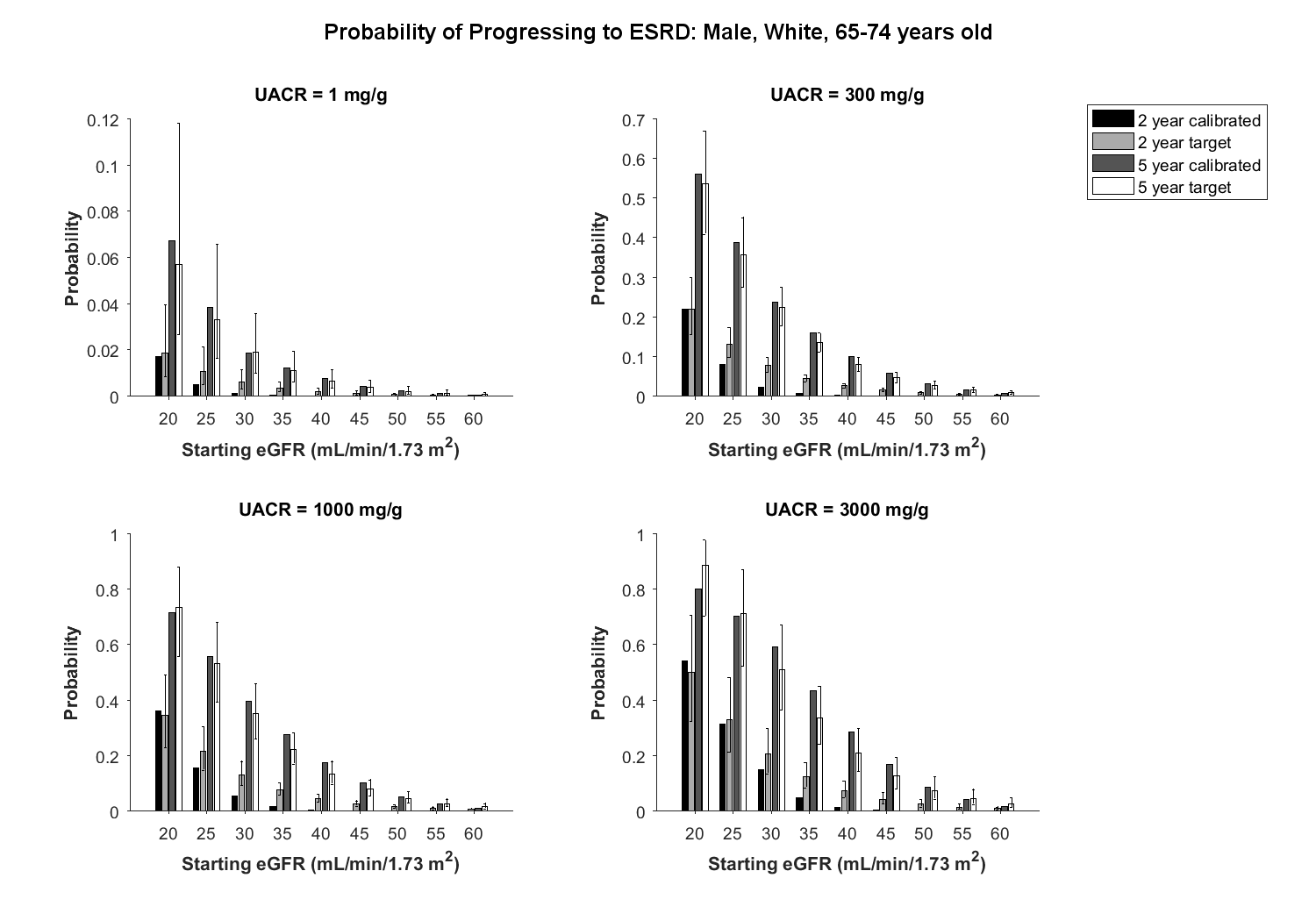
**

**
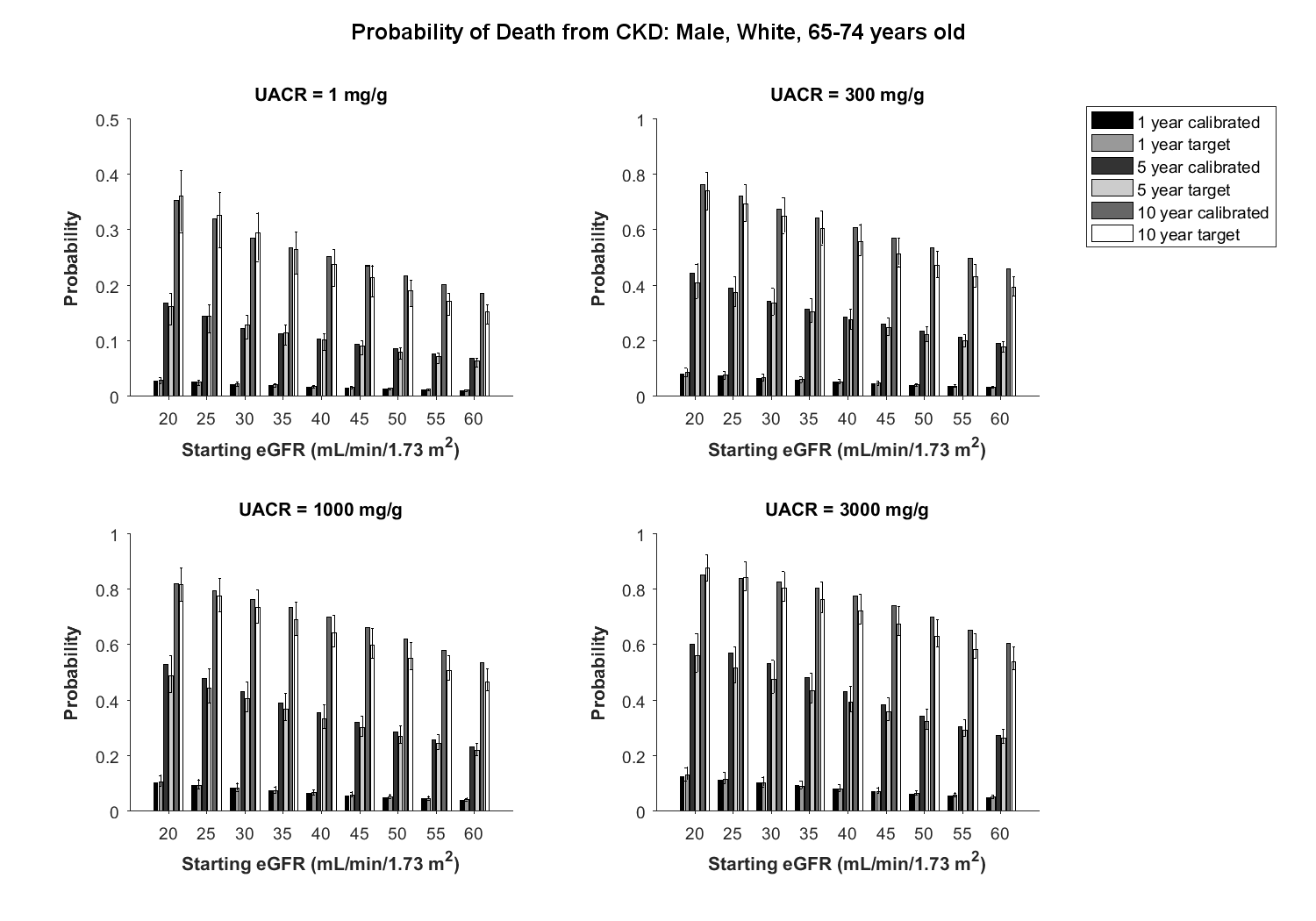
**

**
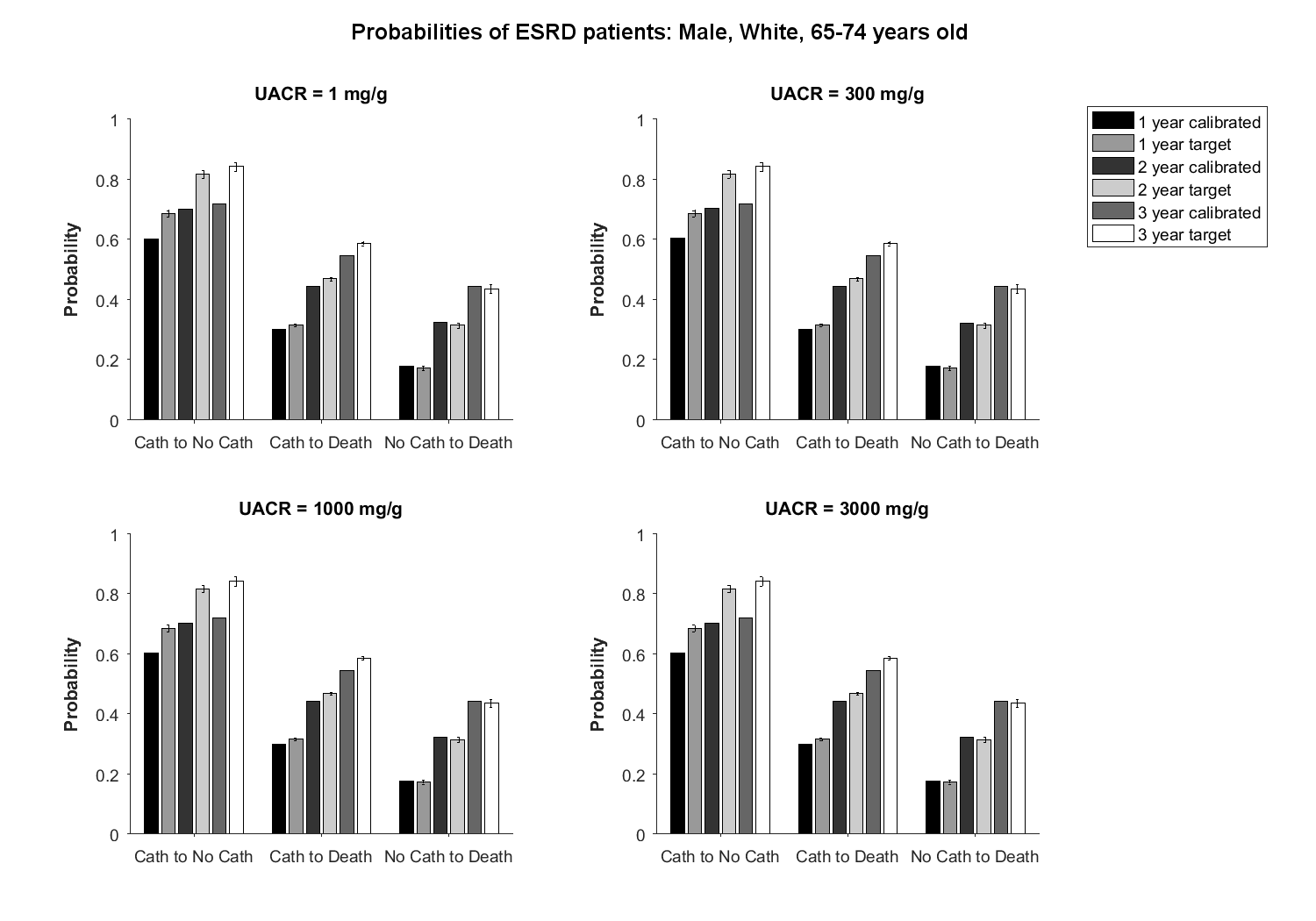
**

**
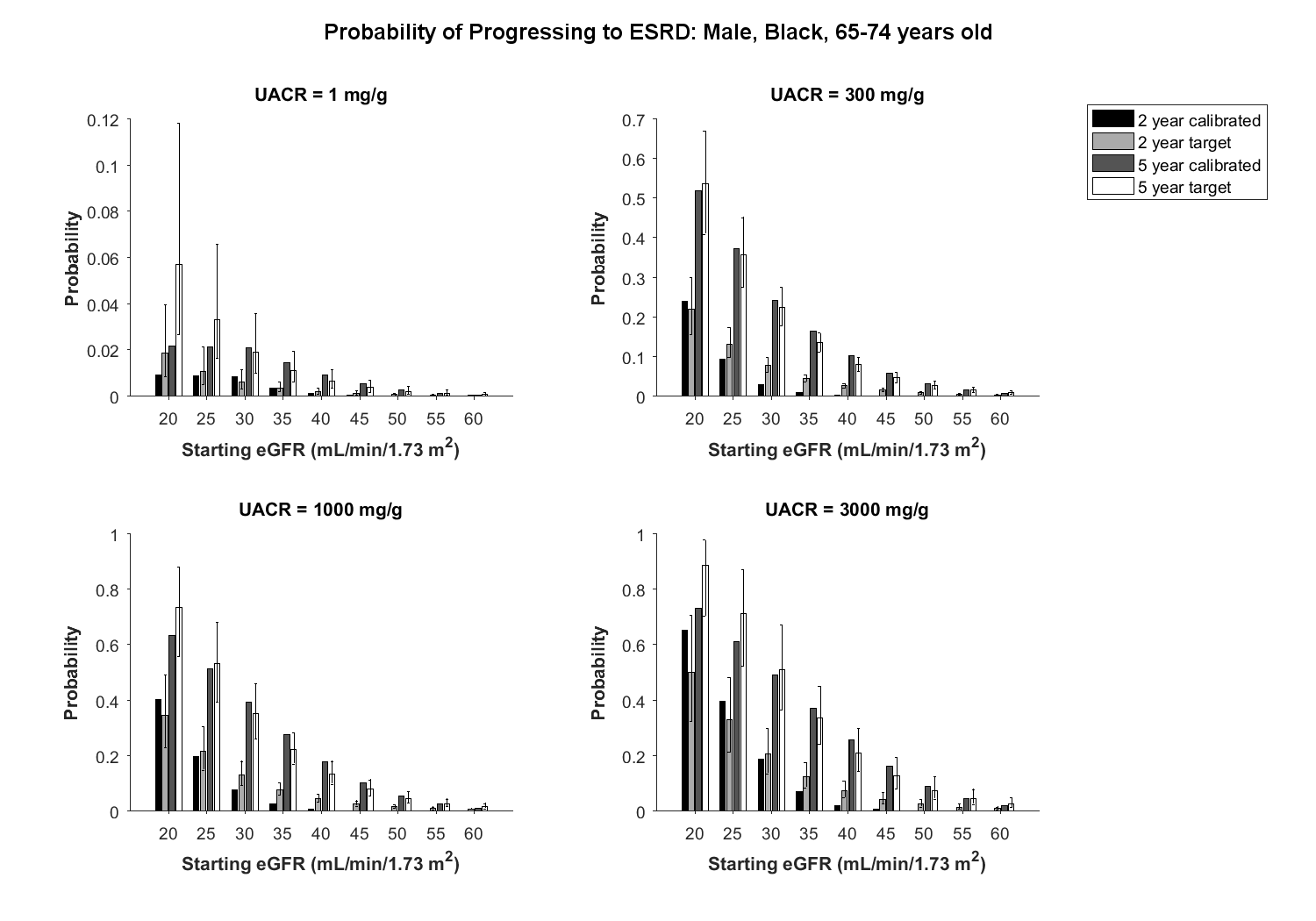
**

**
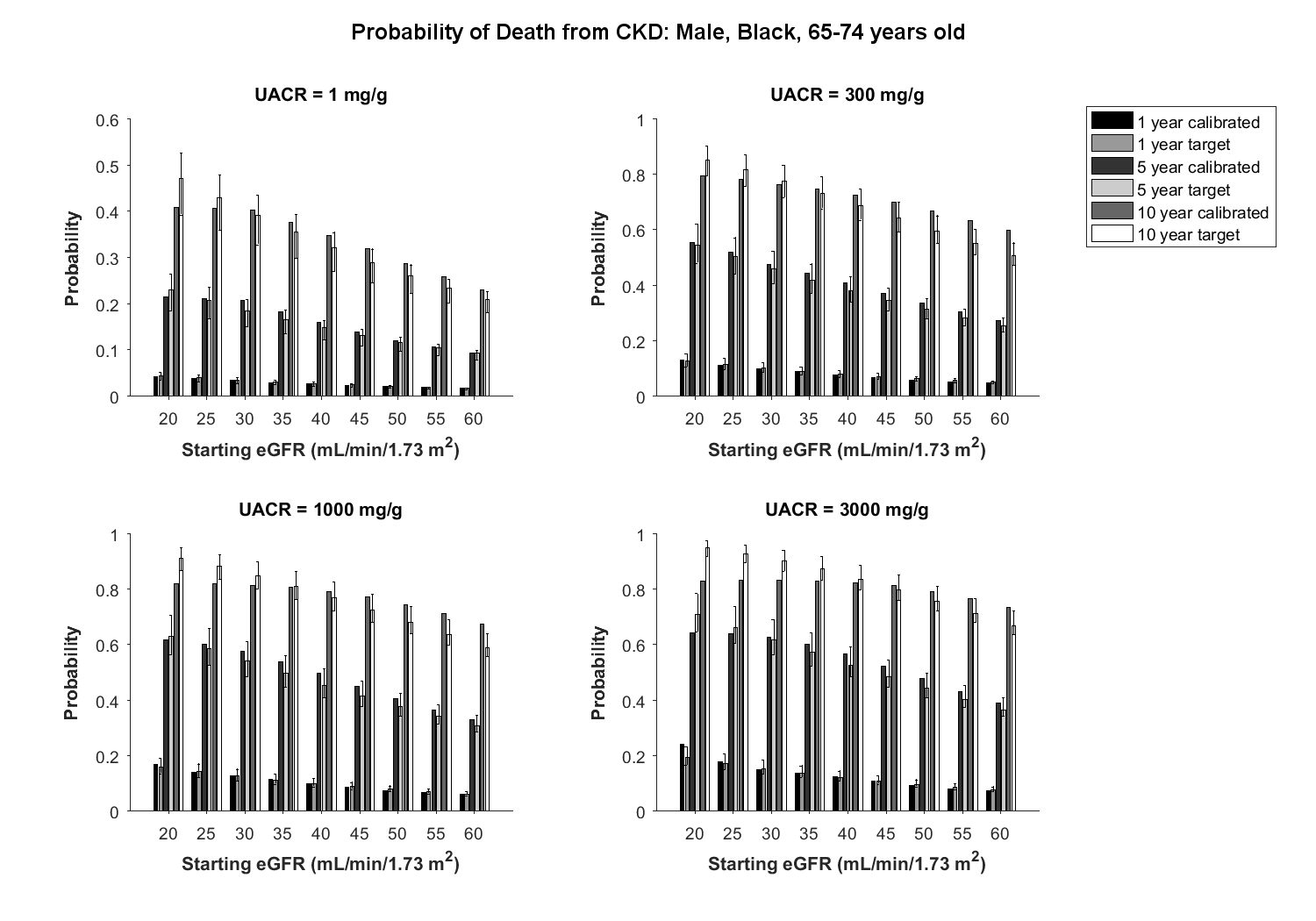
**

**
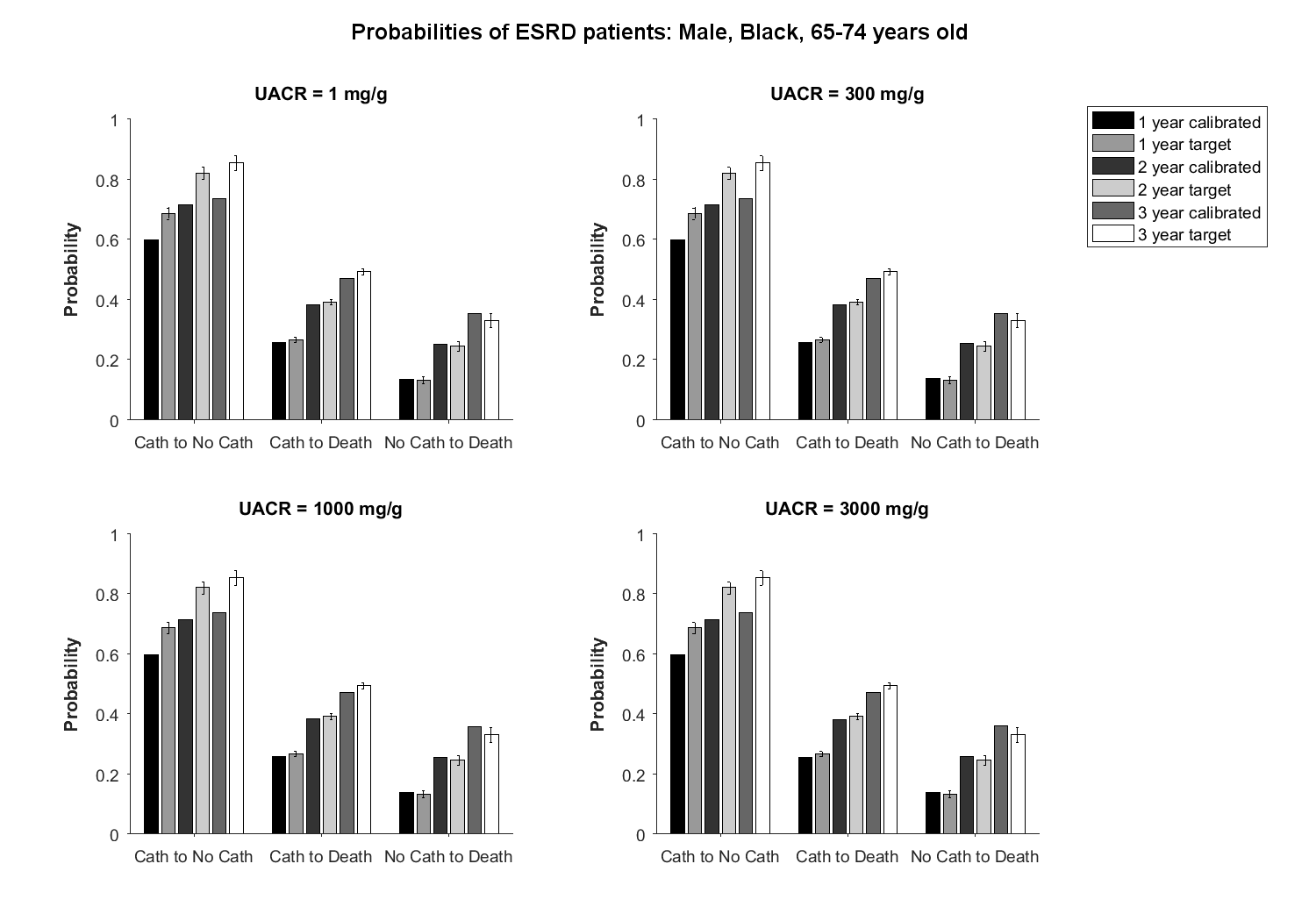
**

**
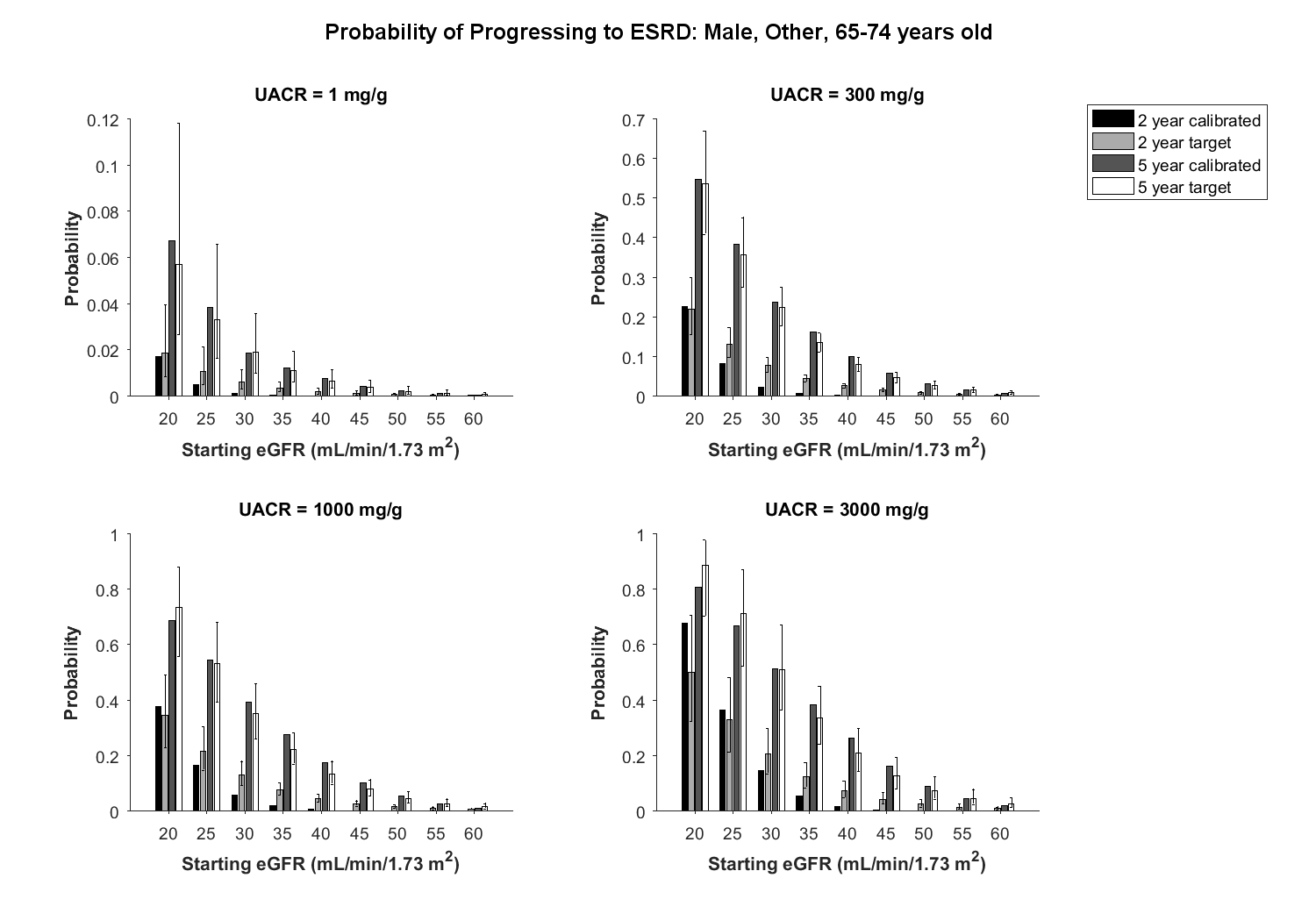
**

**
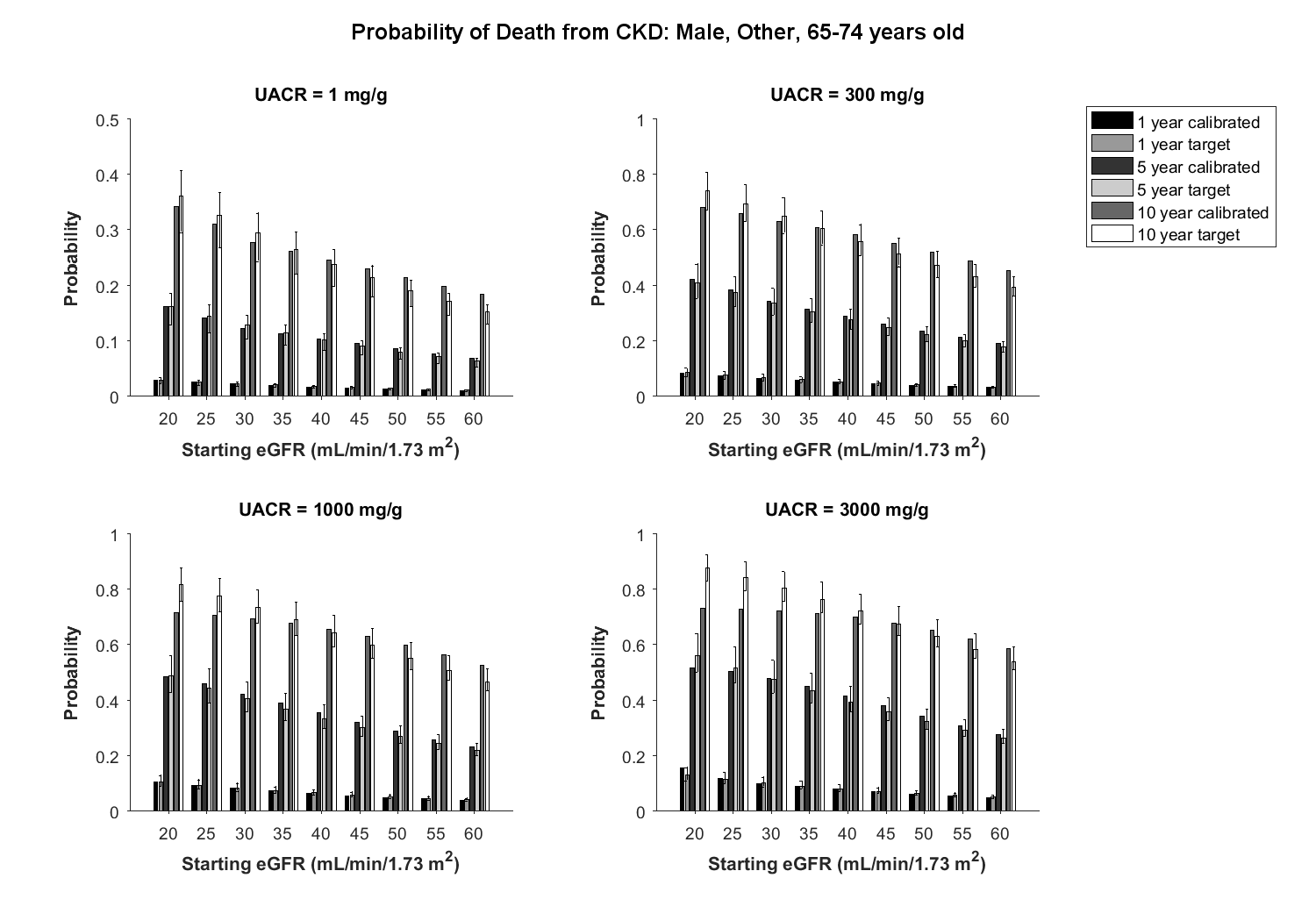
**

**
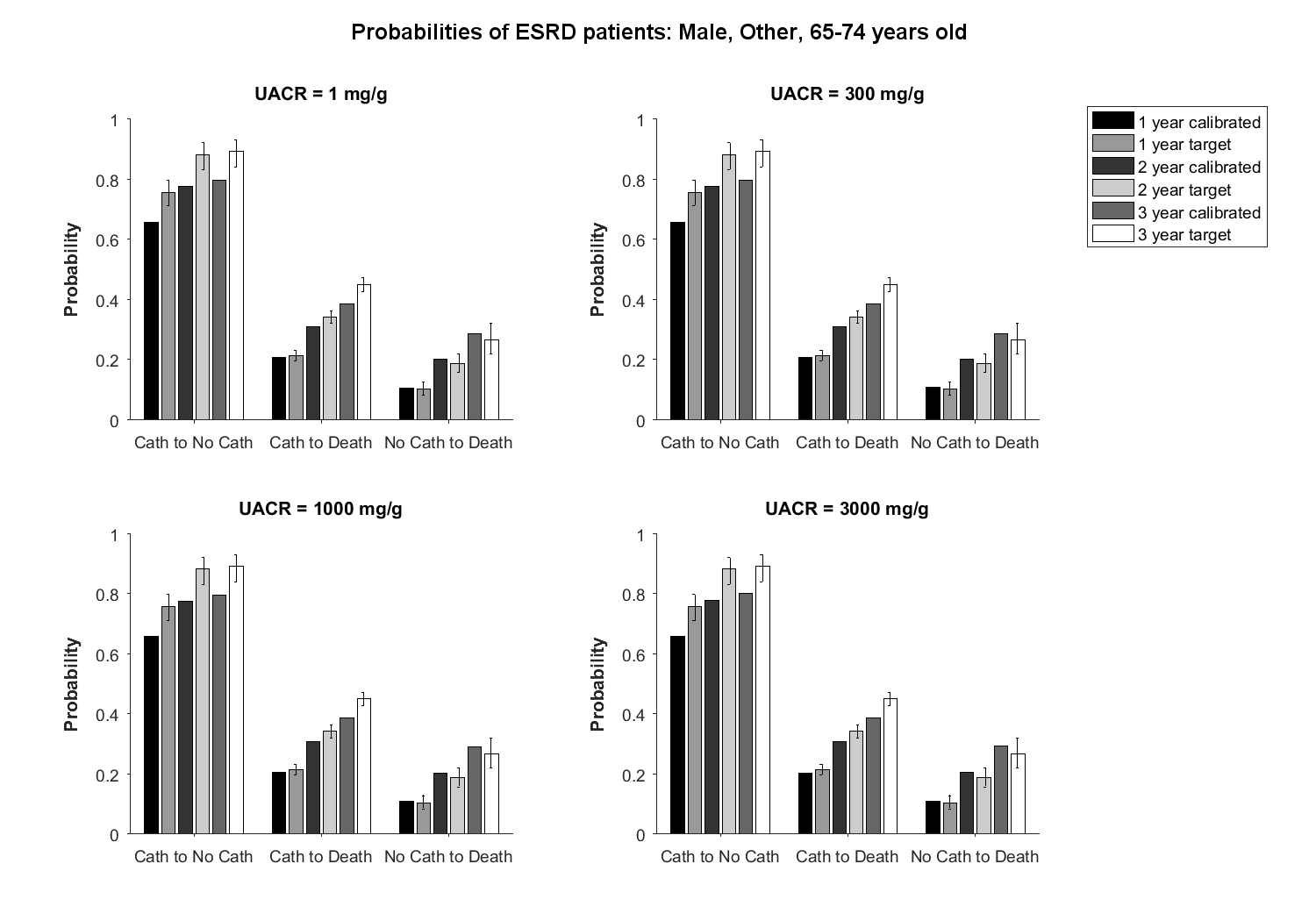
**


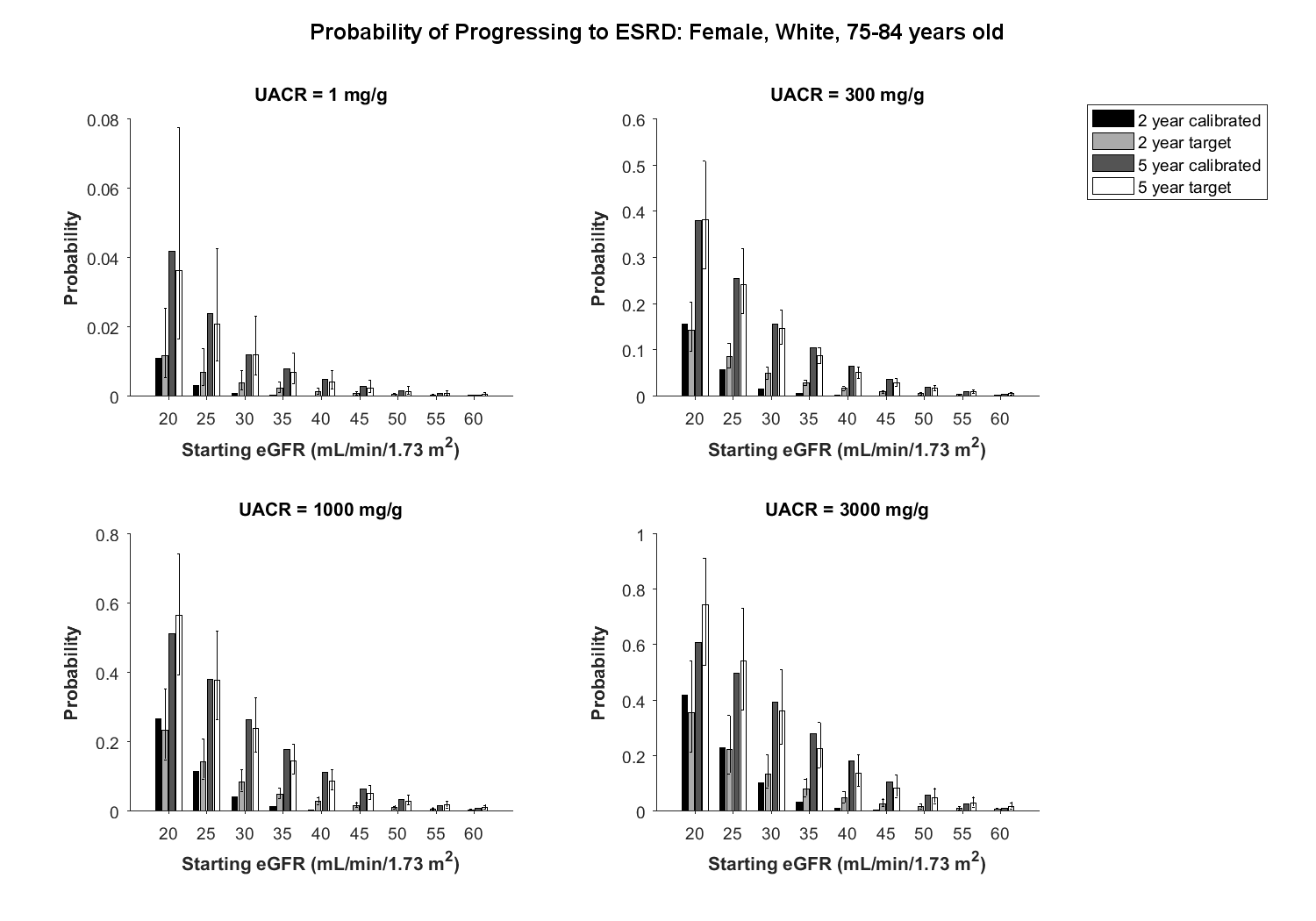


**
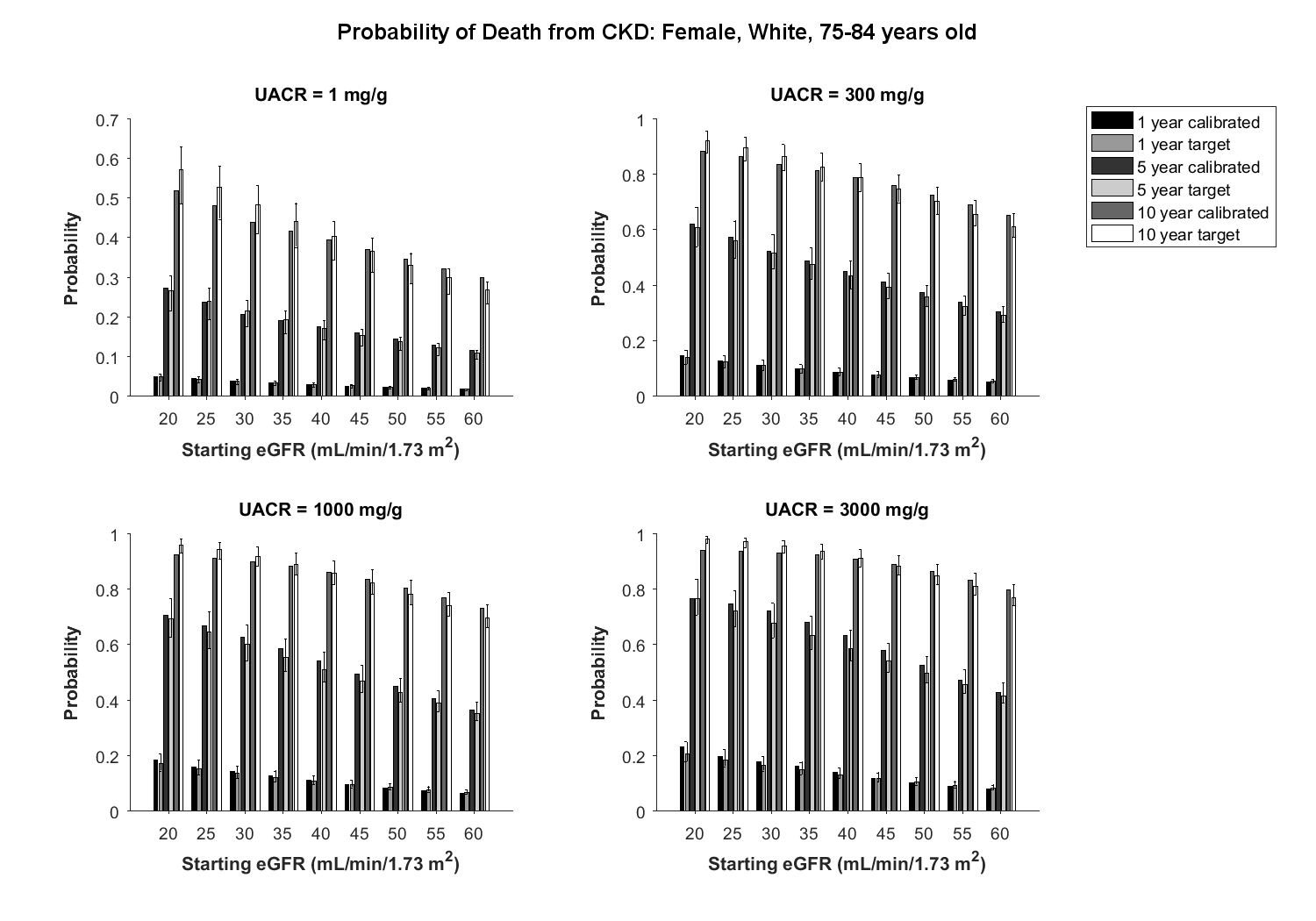
**

**
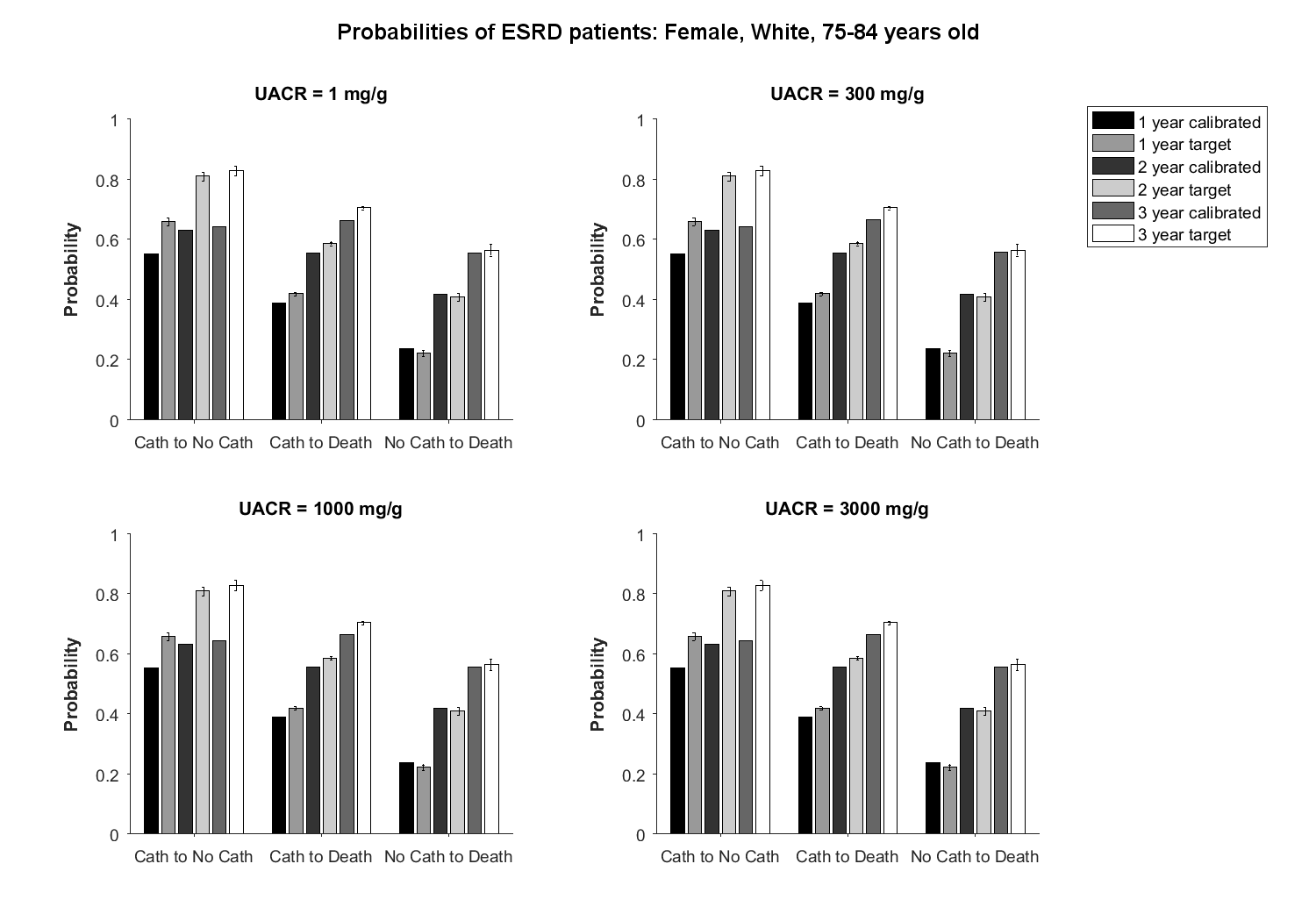
**

**
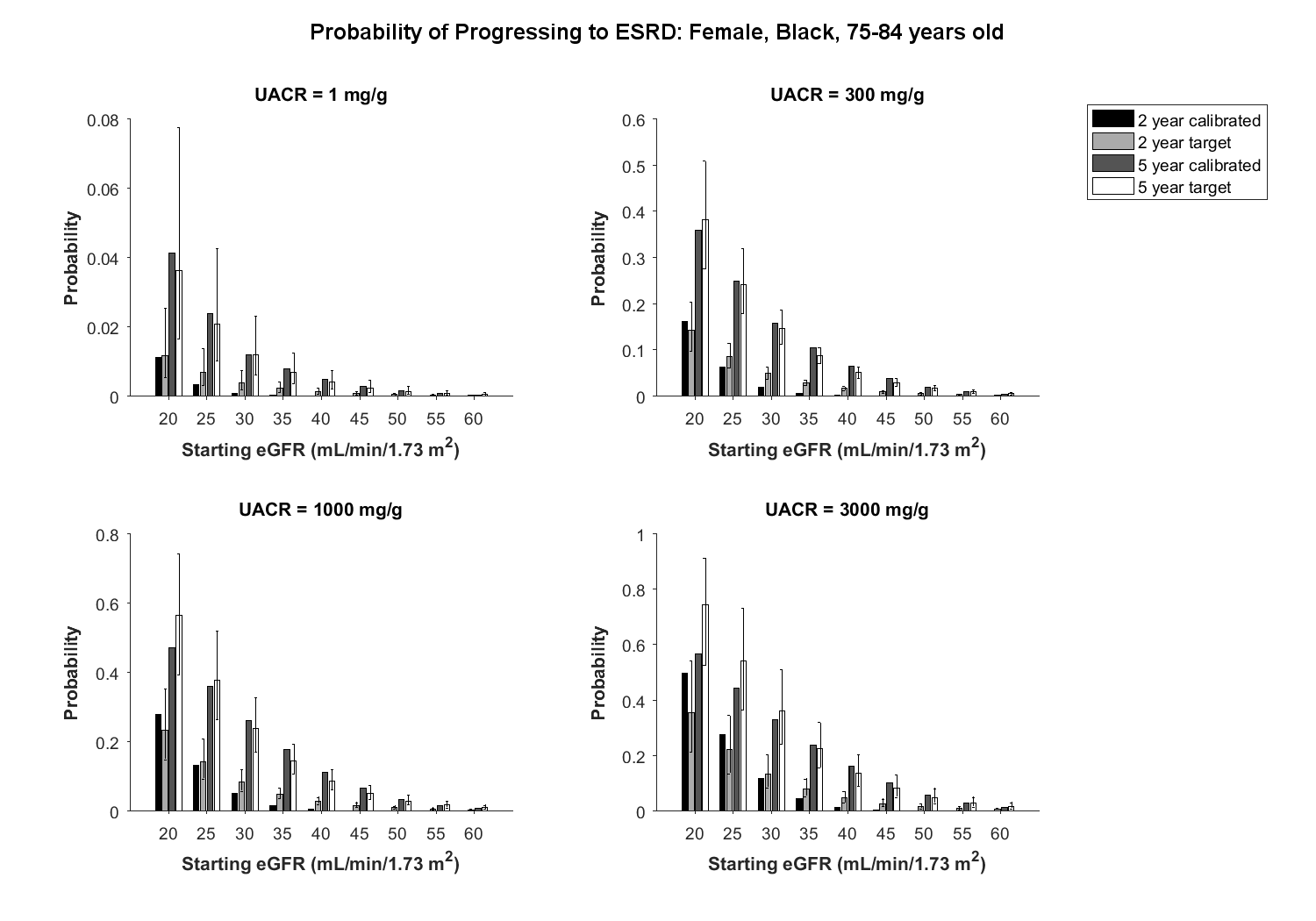
**

**
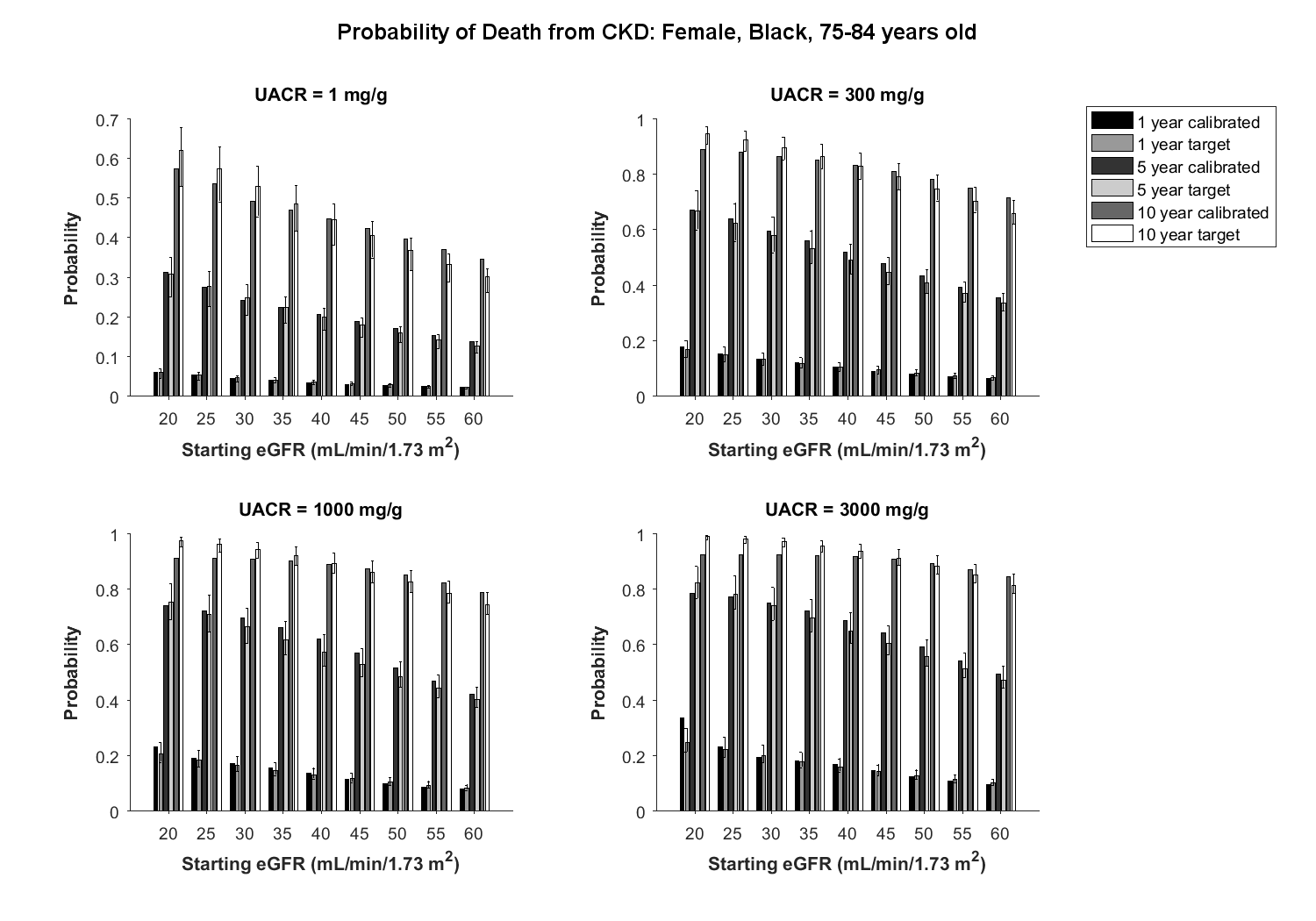
**

**
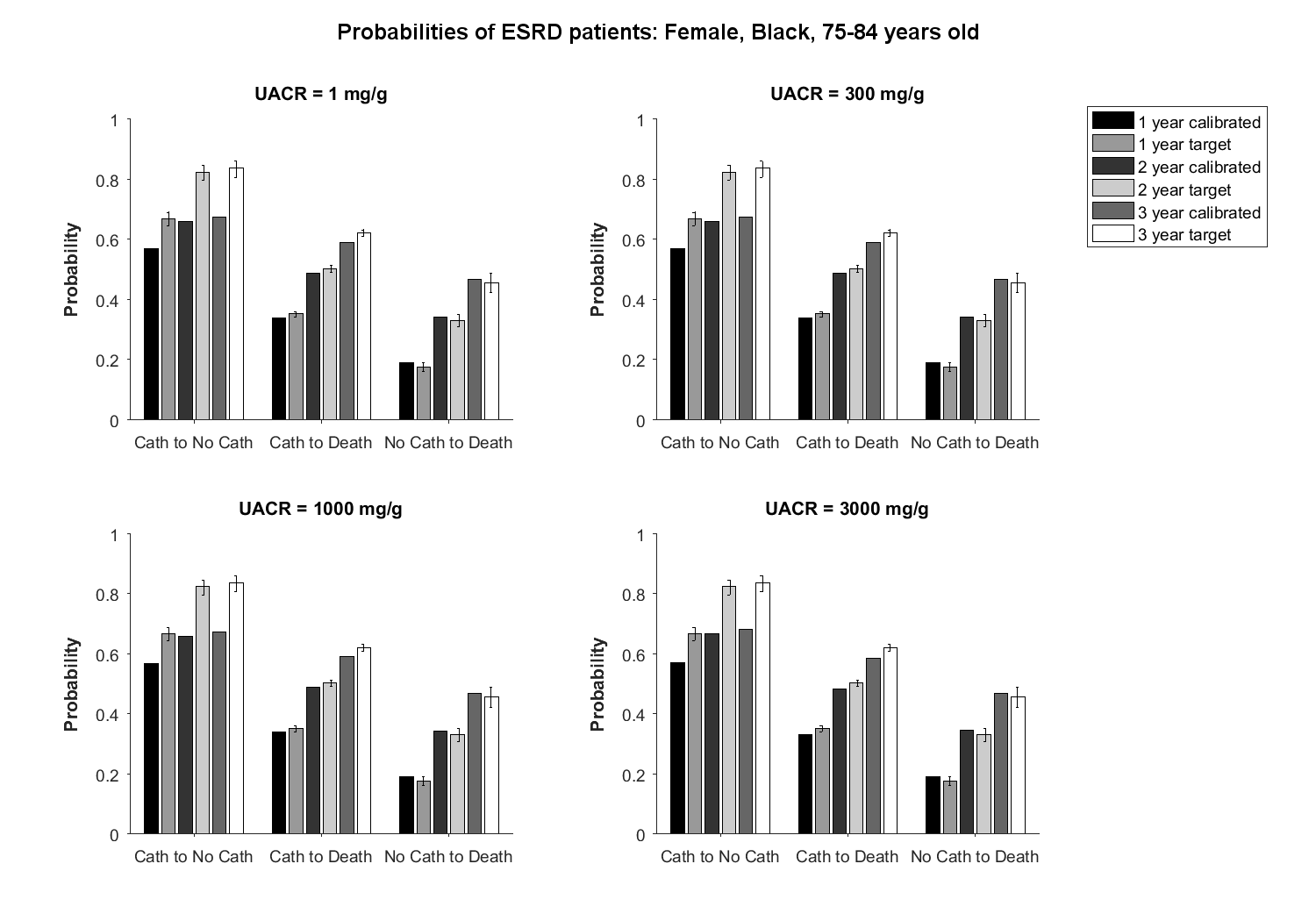
**

**
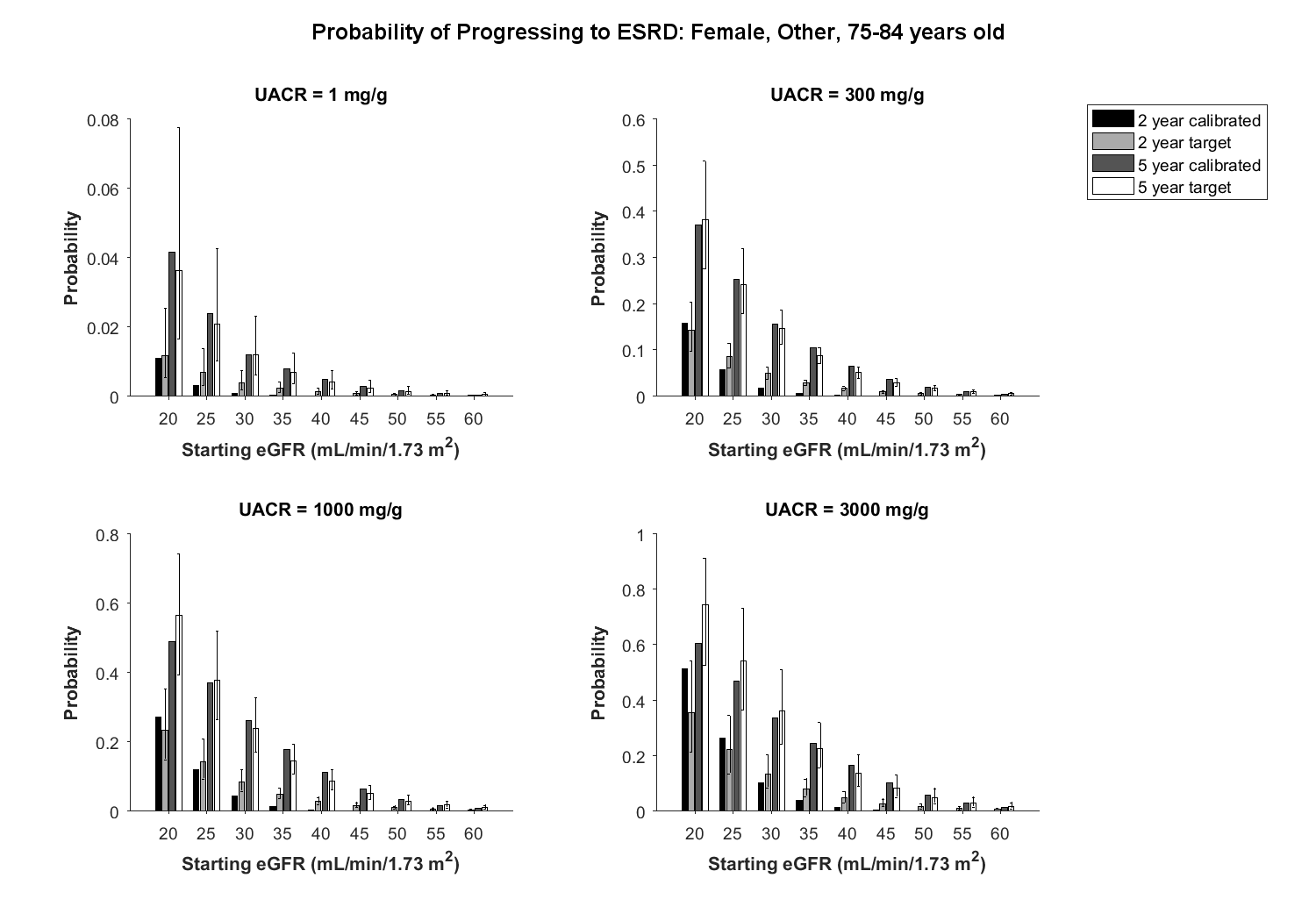
**

**
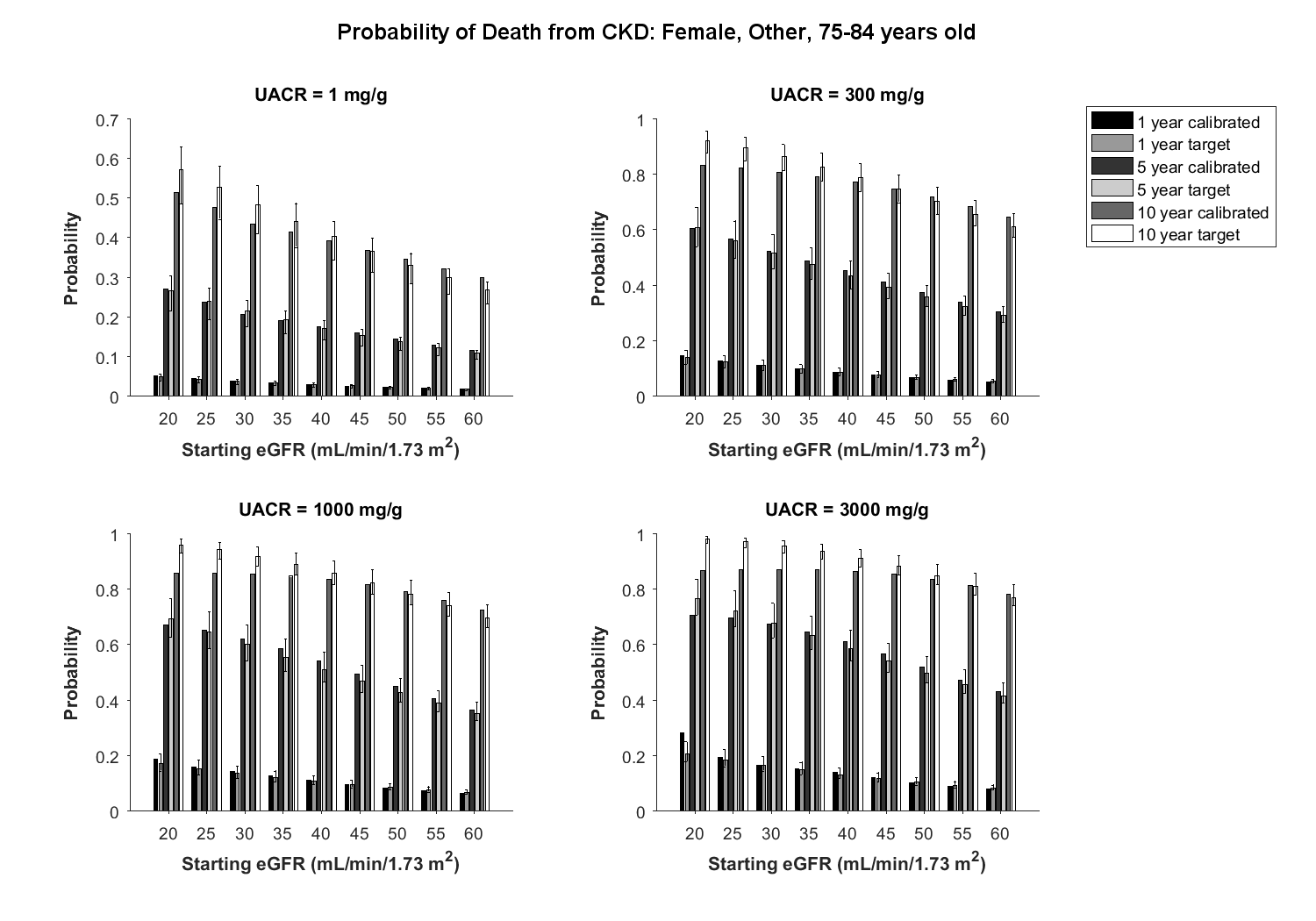
**

**
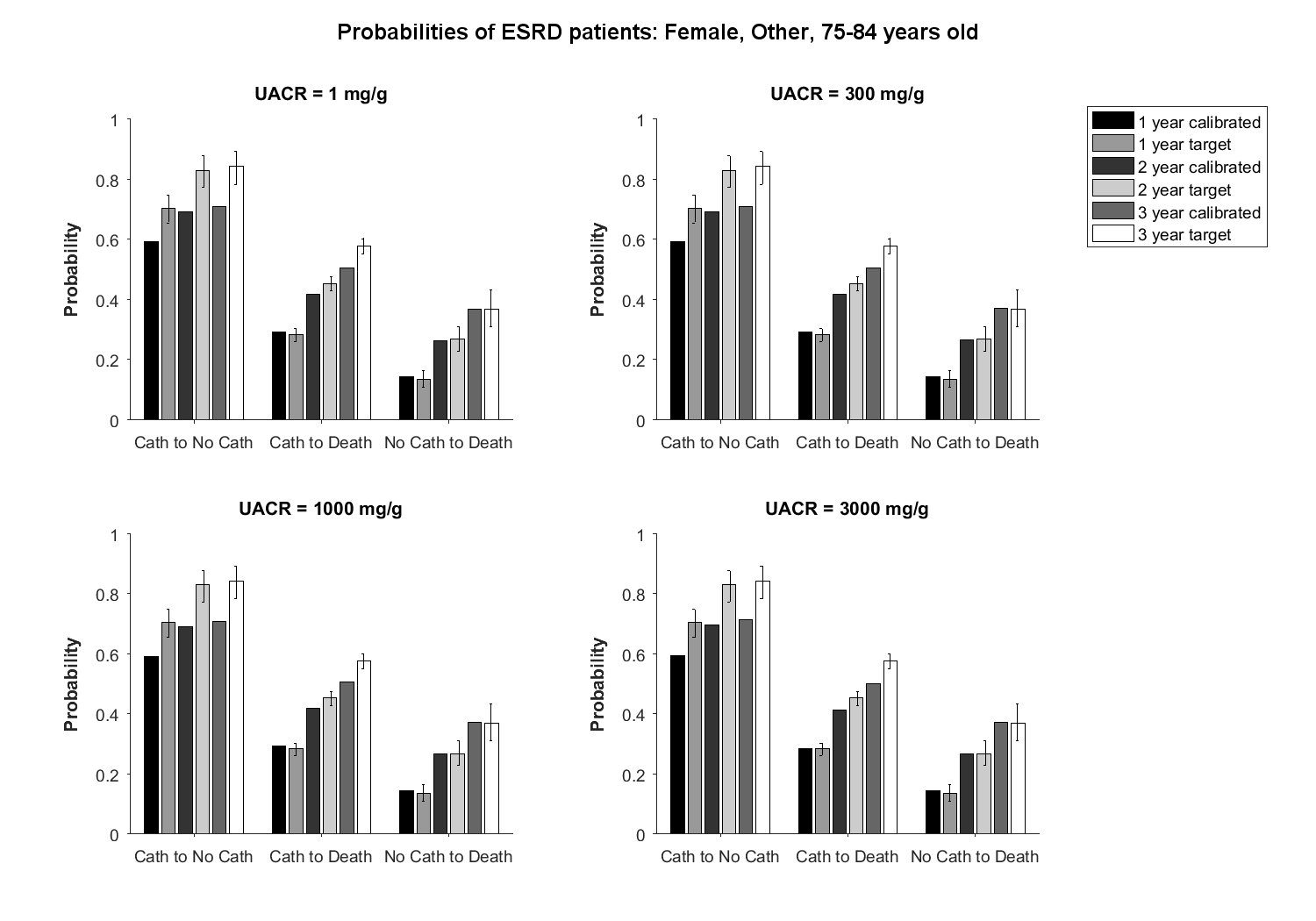
**

**
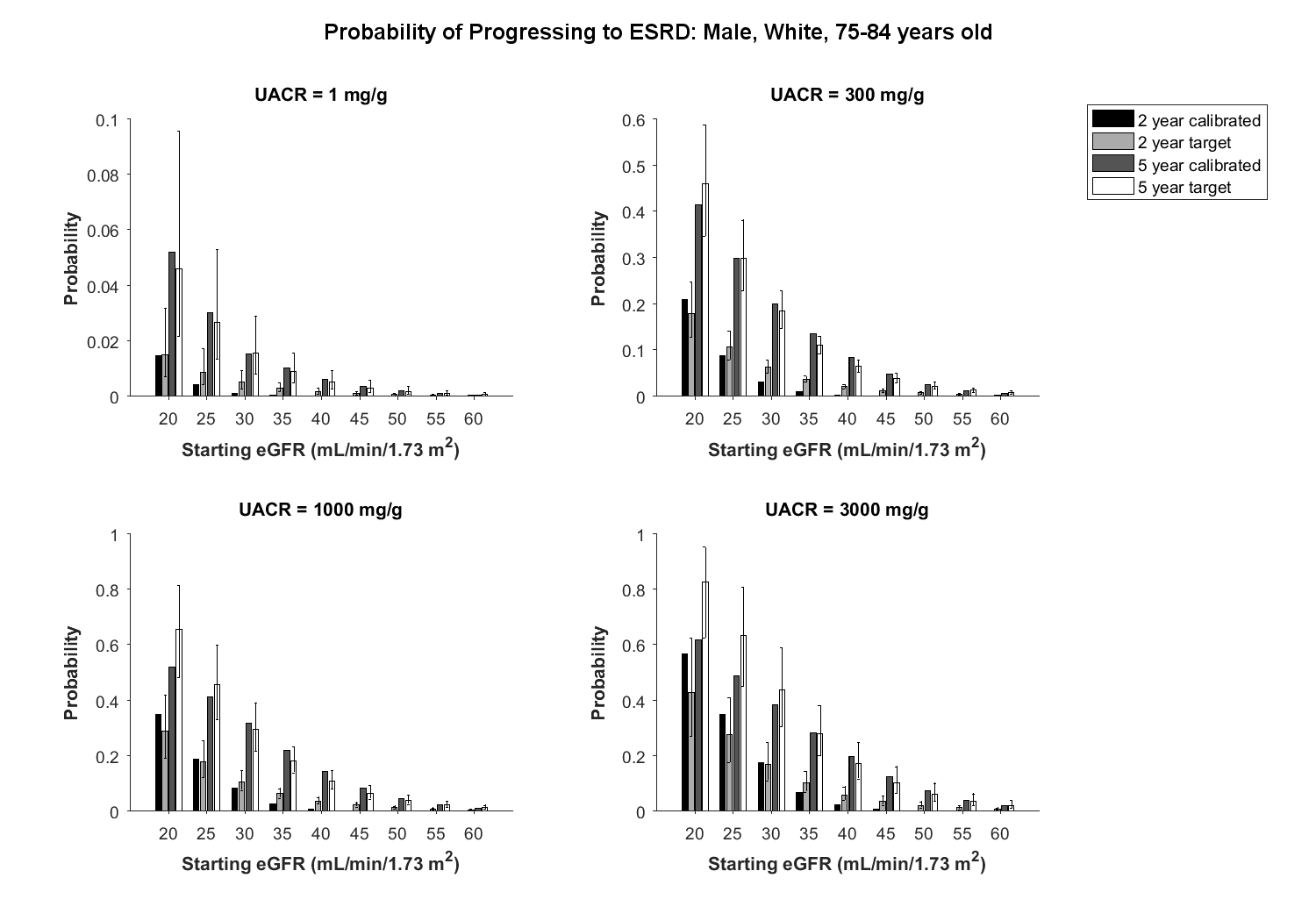
**

**
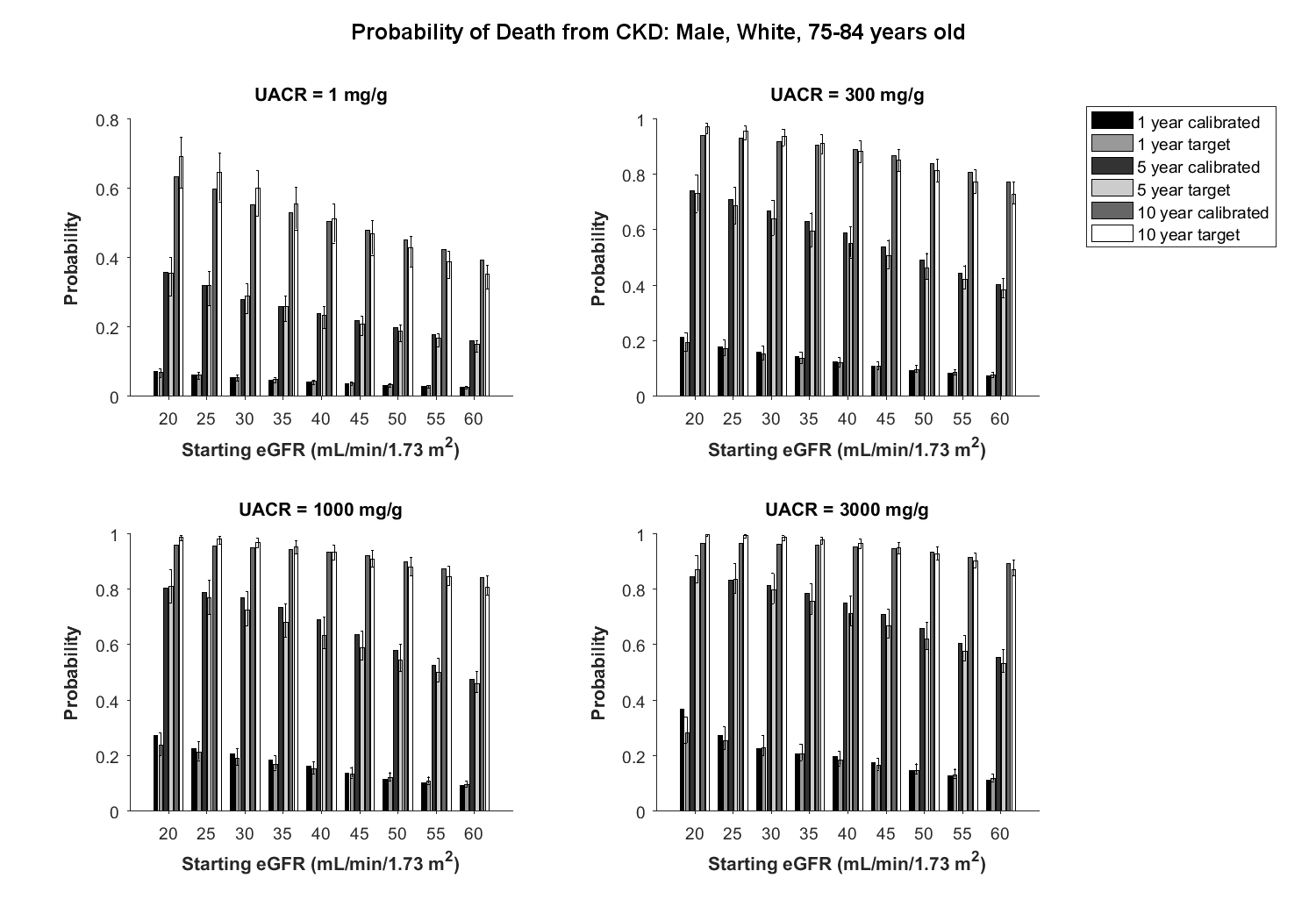
**

**
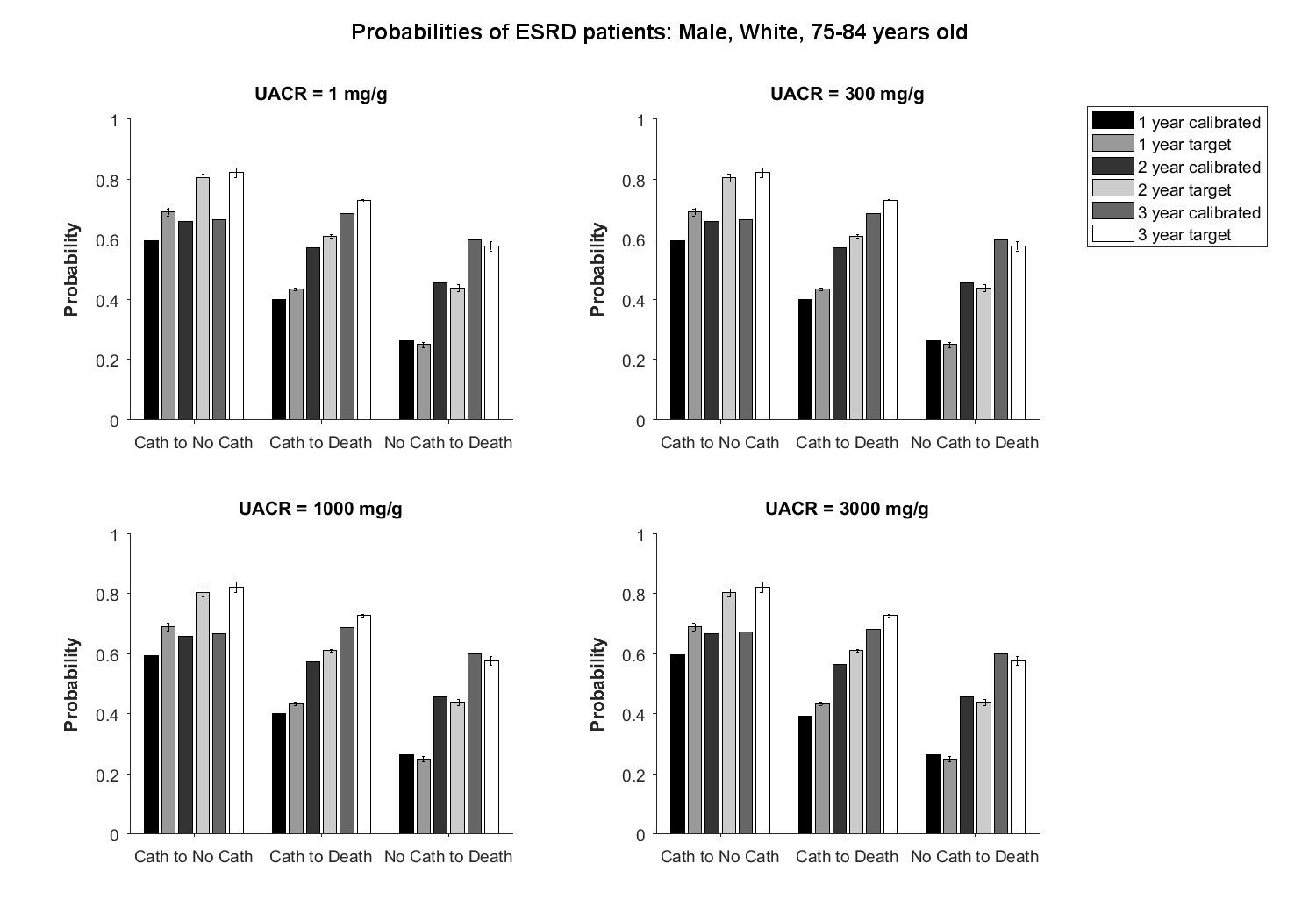
**

**
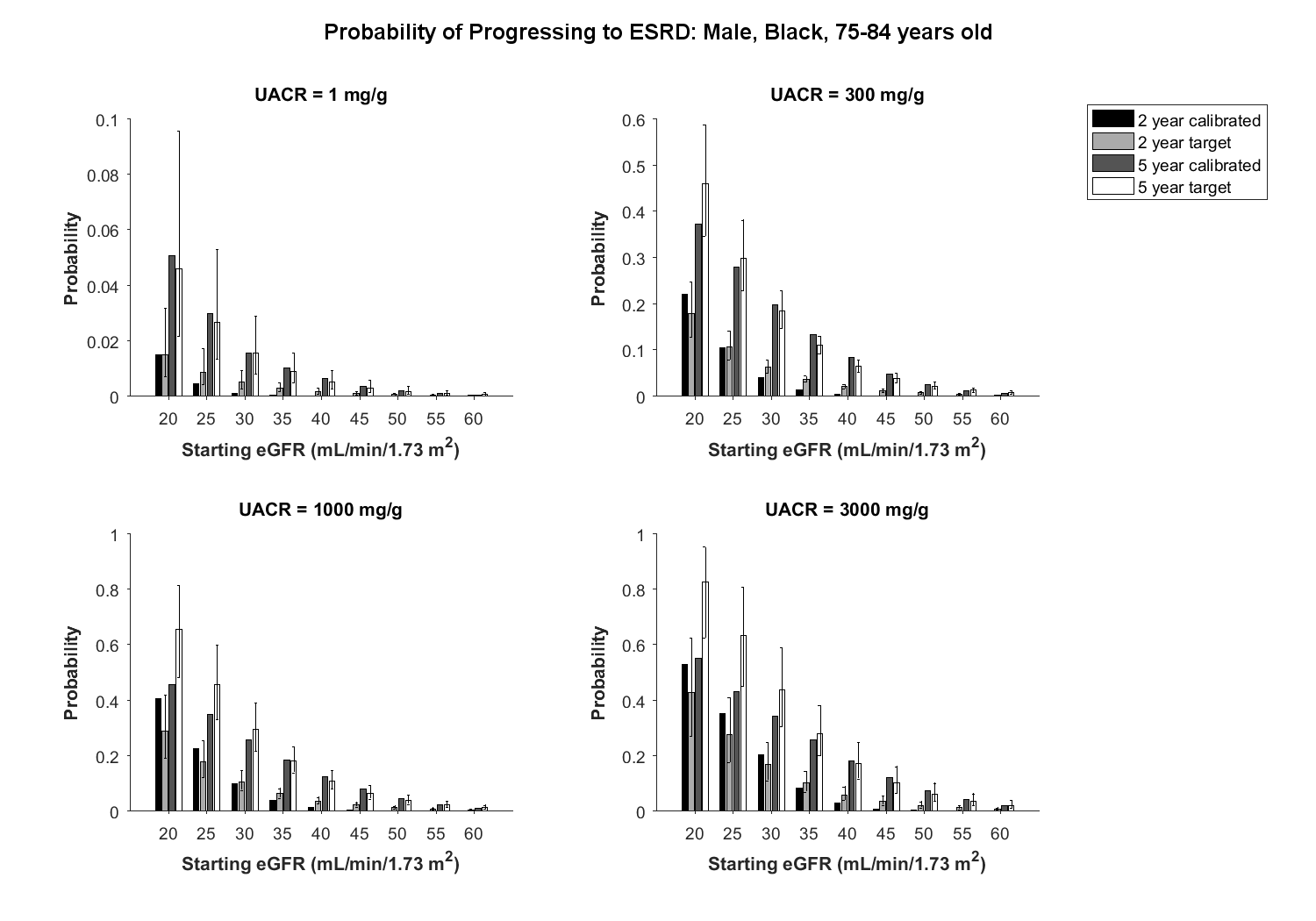
**


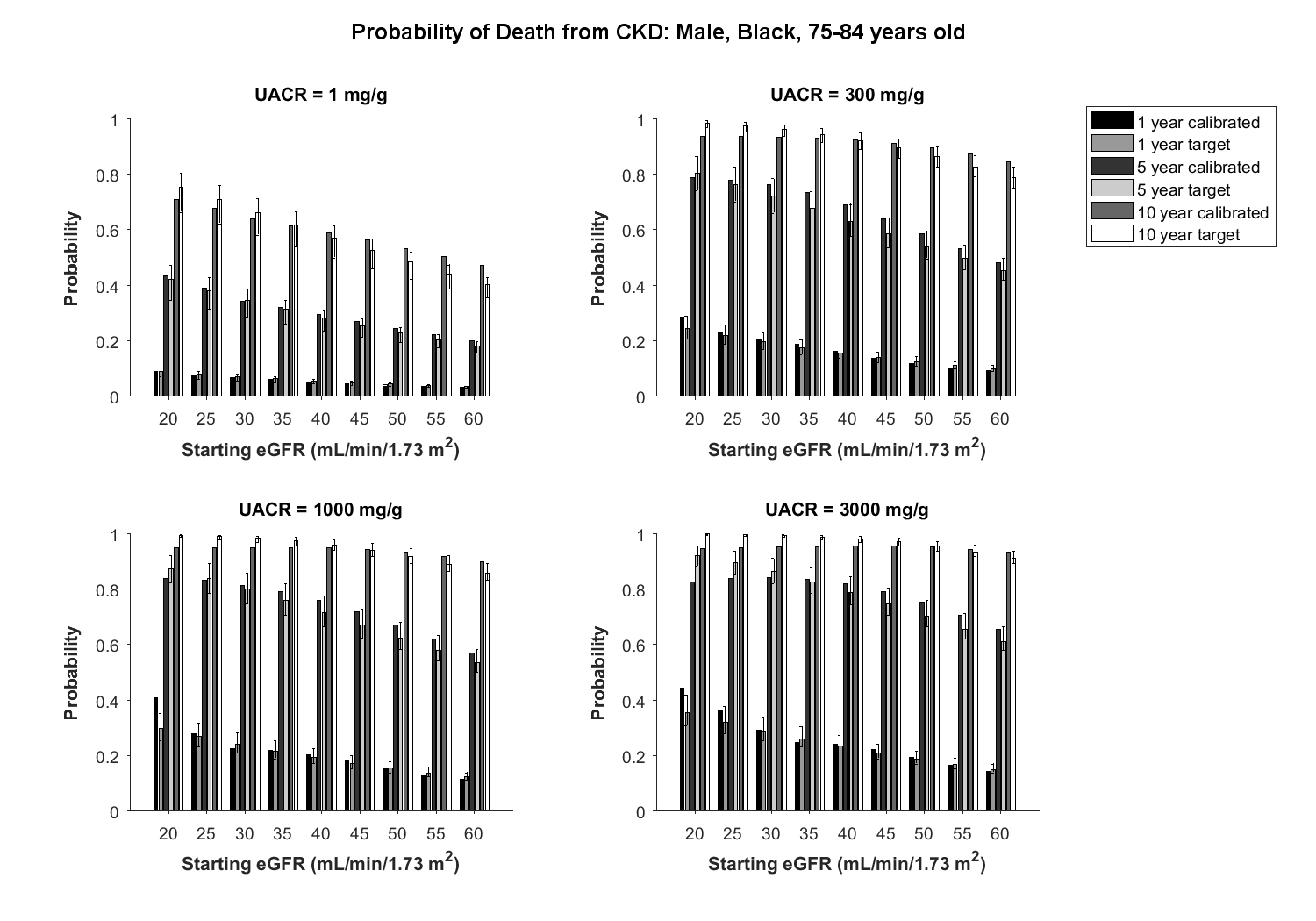


**
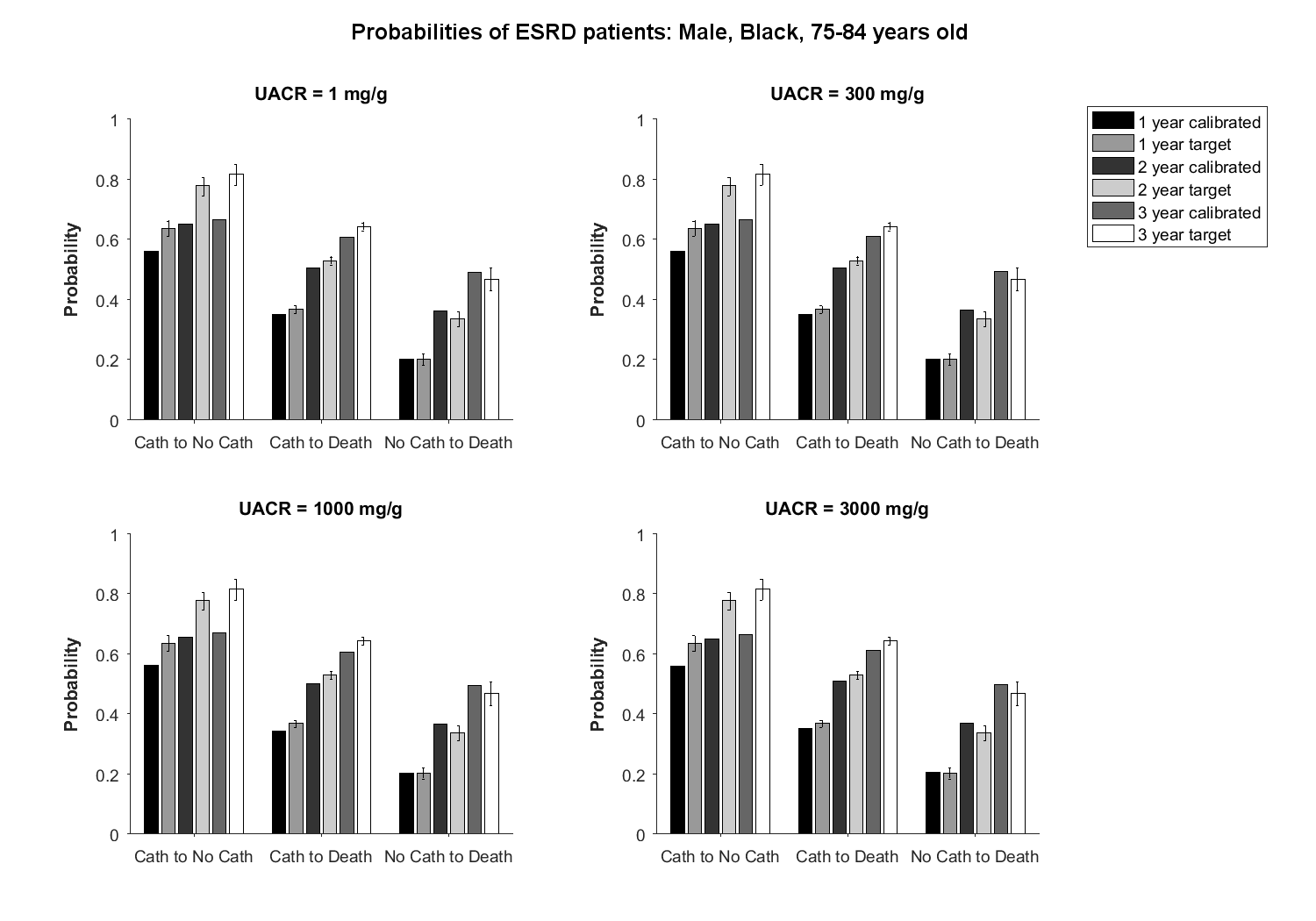
**

**
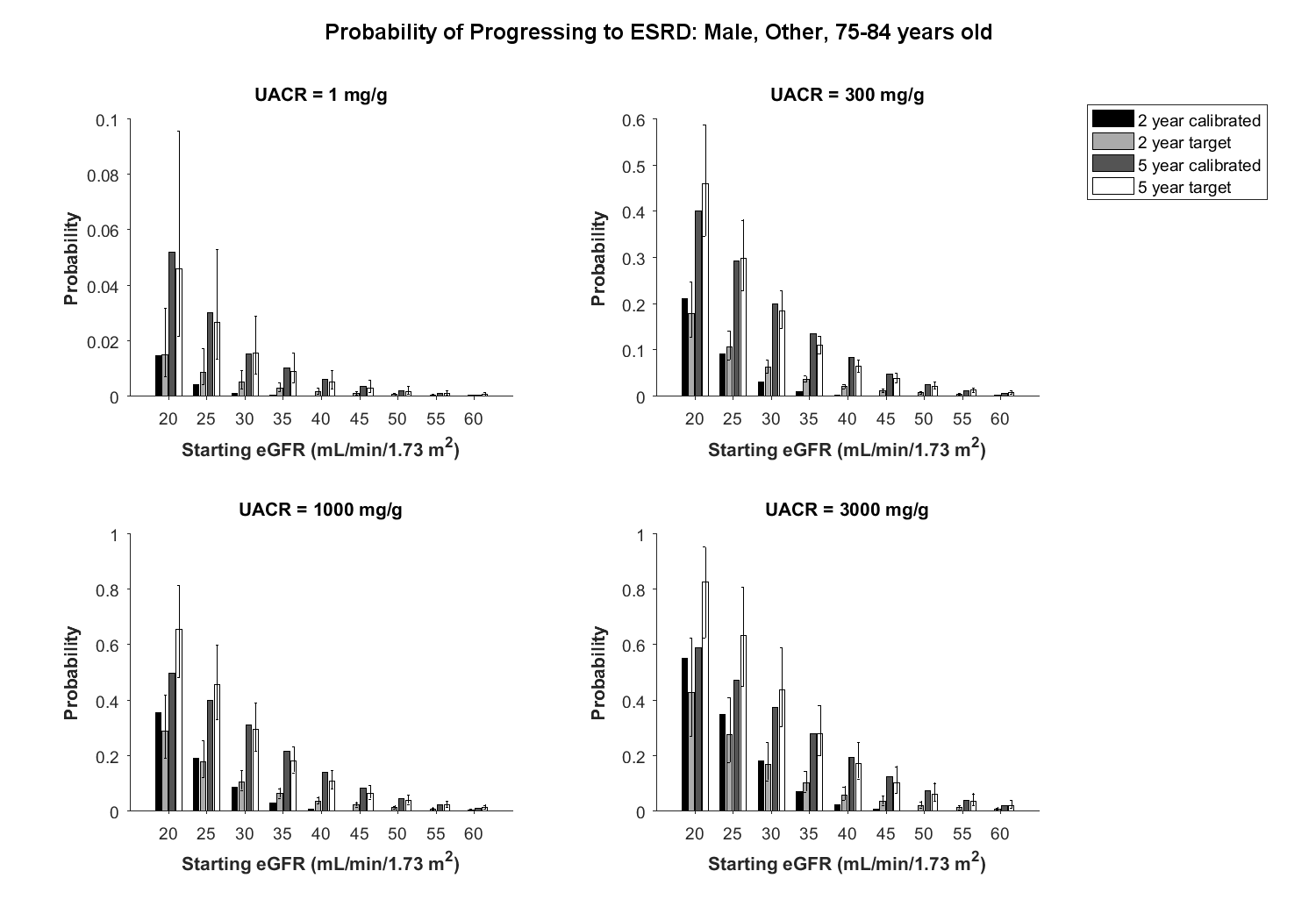
**

**
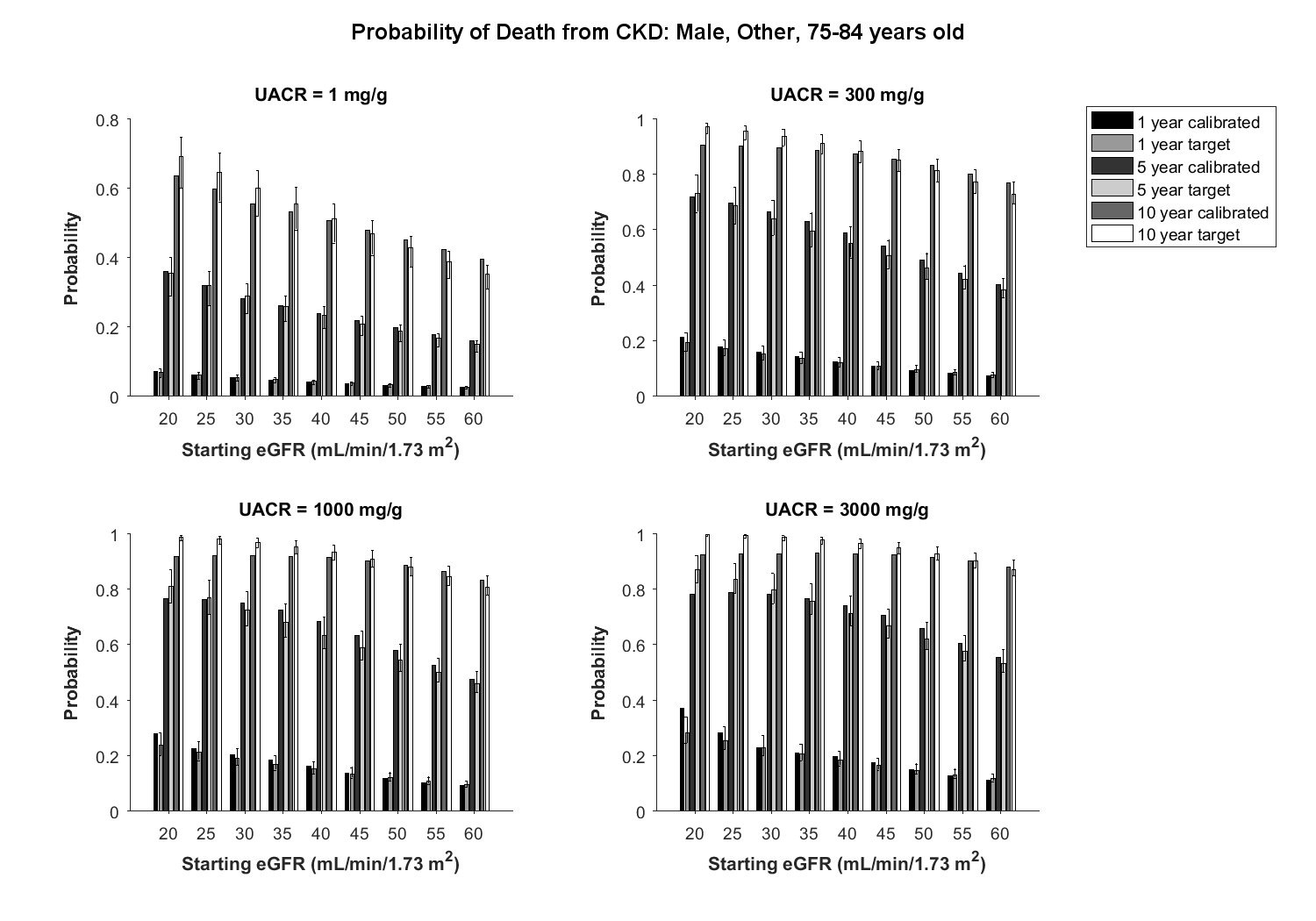
**

**
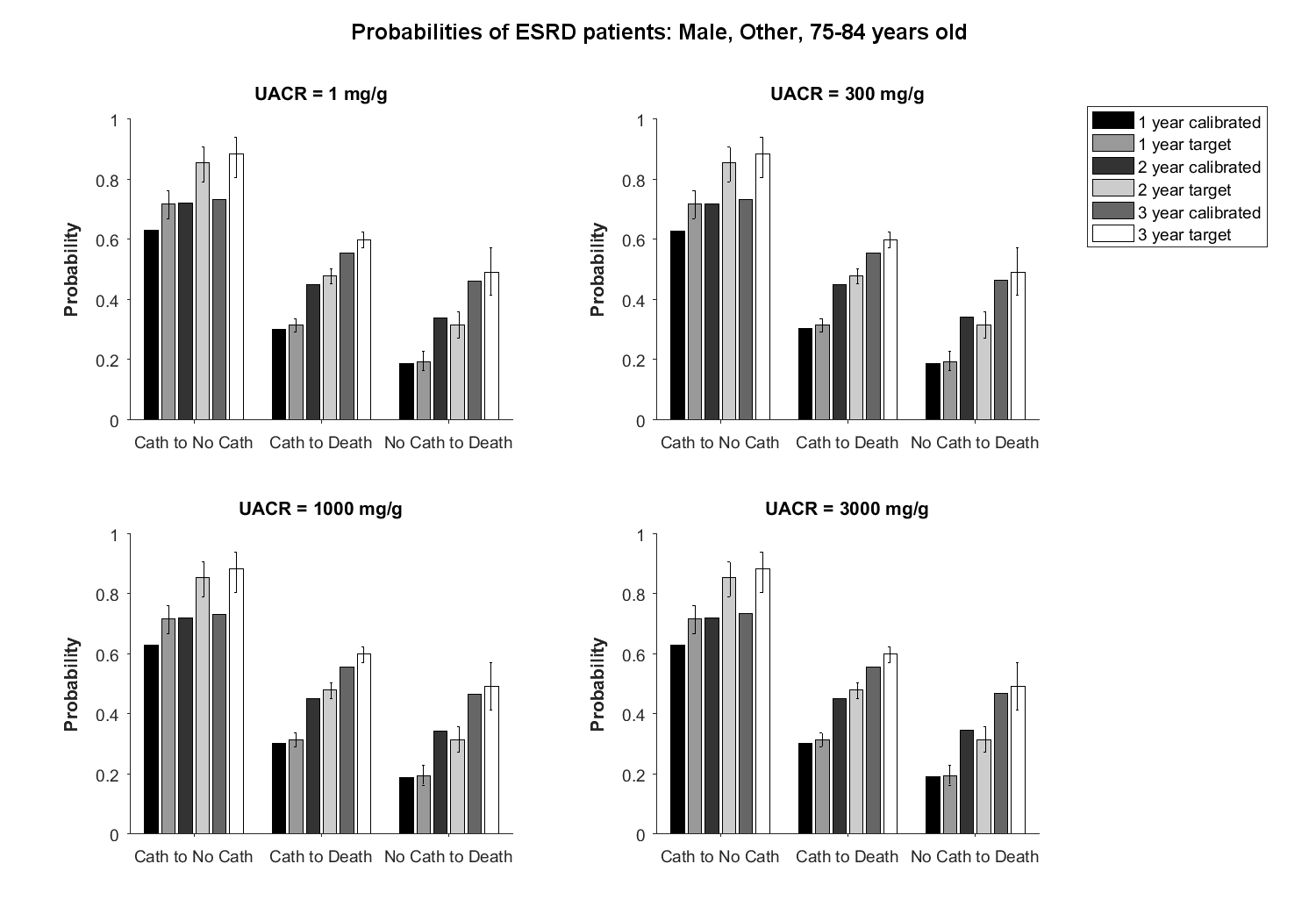
**

Supplement: S2 Appendix — (DOCX) [file pmed.1002532.s002.docx]
