# Supplementary material for: Cost-effectiveness of multidisciplinary care in mild to moderate chronic kidney disease in the United States: A modeling study
Source: PLoS Med. 2018 Mar 27;15(3):e1002532. doi: 10.1371/journal.pmed.1002532 (PMC5870947; doi:10.1371/journal.pmed.1002532)
Supplement: S1 Table — (DOCX) [file pmed.1002532.s003.docx]

**S1 Table: Costs under Multi-Disciplinary Care and Usual Care, by Severity of Kidney Disease**

| **Characteristic** | | **Control** | | **MDC** | | **Change** | |
| --- | --- | --- | --- | --- | --- | --- | --- |
| **eGFR *** | **UACR †** | **Estimate** | **95% CI** | **Estimate** | **95% CI** | **Estimate** | **95% CI** |
| **59** | **1** | $82,739 | ($82,315, $84,366) | $100,157 | ($93,864, $107,851) | $17,419 | ($10,522, $24,357) |
|  | **300** | $65,344 | ($64,198, $65,654) | $75,304 | ($68,971, $80,627) | $9,960 | ($4,040, $15,587) |
|  | **1000** | $61,453 | ($59,879, $61,401) | $69,717 | ($63,505, $74,354) | $8,264 | ($2,923, $13,549) |
|  | **3000** | $58,077 | ($55,770, $57,709) | $64,644 | ($58,674, $70,397) | $6,567 | ($1,881, $13,357) |
| **45** | **1** | $81,637 | ($80,854, $83,499) | $101,383 | ($94,018, $110,057) | $19,746 | ($11,868, $27,589) |
|  | **300** | $66,234 | ($64,862, $66,532) | $77,906 | ($70,005, $84,617) | $11,672 | ($4,222, $18,669) |
|  | **1000** | $62,937 | ($61,218, $62,898) | $72,858 | ($65,003, $79,158) | $9,922 | ($3,087, $16,841) |
|  | **3000** | $60,115 | ($57,737, $59,785) | $68,318 | ($60,522, $76,183) | $8,203 | ($1,811, $17,170) |
| **30** | **1** | $84,343 | ($83,302, $86,406) | $106,908 | ($98,452, $116,693) | $22,565 | ($13,508, $31,643) |
|  | **300** | $72,341 | ($70,851, $72,732) | $85,570 | ($76,318, $93,448) | $13,229 | ($4,506, $21,421) |
|  | **1000** | $70,962 | ($68,661, $71,226) | $82,166 | ($72,917, $89,676) | $11,204 | ($3,082, $19,465) |
|  | **3000** | $70,425 | ($66,628, $70,789) | $79,289 | ($70,321, $88,089) | $8,864 | ($1,310, $19,034) |
| **Overall** | | $68,571 | ($67,503, $68,674) | $80,572 | ($73,194, $87,494) | $12,001 | ($5,098, $19,358) |

Abbreviations: QALY = quality-adjusted life year, eGFR = estimated glomerular filtration rate, UACR = urine albumin to creatinine ratio, ICER = incremental cost-effectiveness ratio, CI = confidence interval

* Estimated glomerular filtration rate units in mL/min/1.73 m^2^

† Urine albumin to creatinine ratio units in mg/g
