# Supplementary material for: Cost-effectiveness of multidisciplinary care in mild to moderate chronic kidney disease in the United States: A modeling study
Source: PLoS Med. 2018 Mar 27;15(3):e1002532. doi: 10.1371/journal.pmed.1002532 (PMC5870947; doi:10.1371/journal.pmed.1002532)
Supplement: S2 Table — (DOCX) [file pmed.1002532.s004.docx]

**S2 Table: Quality-Adjusted Life Years under Multi-Disciplinary Care and Usual Care, by Severity of Kidney Disease**

| **Characteristic** | | **Control** | | **MDC** | | **Change** | |
| --- | --- | --- | --- | --- | --- | --- | --- |
| **eGFR *** | **UACR †** | **Estimate** | **95% CI** | **Estimate** | **95% CI** | **Estimate** | **95% CI** |
| **59** | **1** | 2.75 | (2.39, 3.09) | 3.05 | (2.62, 3.49) | 0.30 | (0.10, 0.50) |
|  | **300** | 1.88 | (1.60, 2.10) | 2.09 | (1.75, 2.37) | 0.20 | (0.07, 0.35) |
|  | **1000** | 1.72 | (1.46, 1.93) | 1.89 | (1.58, 2.14) | 0.16 | (0.05, 0.29) |
|  | **3000** | 1.59 | (1.34, 1.77) | 1.72 | (1.44, 1.96) | 0.13 | (0.04, 0.29) |
| **45** | **1** | 2.59 | (2.26, 2.92) | 2.94 | (2.52, 3.39) | 0.35 | (0.12, 0.58) |
|  | **300** | 1.66 | (1.40, 1.86) | 1.91 | (1.58, 2.20) | 0.25 | (0.09, 0.43) |
|  | **1000** | 1.48 | (1.24, 1.67) | 1.68 | (1.38, 1.95) | 0.20 | (0.07, 0.37) |
|  | **3000** | 1.34 | (1.11, 1.51) | 1.50 | (1.23, 1.76) | 0.16 | (0.05, 0.37) |
| **30** | **1** | 2.51 | (2.19, 2.83) | 2.91 | (2.48, 3.38) | 0.41 | (0.14, 0.67) |
|  | **300** | 1.48 | (1.23, 1.68) | 1.77 | (1.45, 2.09) | 0.29 | (0.11, 0.50) |
|  | **1000** | 1.29 | (1.05, 1.48) | 1.52 | (1.22, 1.81) | 0.23 | (0.08, 0.43) |
|  | **3000** | 1.14 | (0.91, 1.32) | 1.32 | (1.05, 1.61) | 0.18 | (0.06, 0.41) |
| **Overall** | | 1.78 | (1.52, 2.01) | 2.02 | (1.69, 2.33) | 0.23 | (0.08, 0.42) |

Abbreviations: QALY = quality-adjusted life year, eGFR = estimated glomerular filtration rate, UACR = urine albumin to creatinine ratio, ICER = incremental cost-effectiveness ratio, CI = confidence interval

* Estimated glomerular filtration rate units in mL/min/1.73 m^2^

† Urine albumin to creatinine ratio units in mg/g
