# Supplementary material for: Cost-effectiveness of multidisciplinary care in mild to moderate chronic kidney disease in the United States: A modeling study
Source: PLoS Med. 2018 Mar 27;15(3):e1002532. doi: 10.1371/journal.pmed.1002532 (PMC5870947; doi:10.1371/journal.pmed.1002532)
Supplement: S3 Table — (DOCX) [file pmed.1002532.s005.docx]

**S3 Table: Costs under Multi-Disciplinary Care and Usual Care, by Age**

| **Characteristic** | | | **Control** | | **MDC** | | **Change** | |
| --- | --- | --- | --- | --- | --- | --- | --- | --- |
| **Age (yrs)** | **eGFR *** | **UACR †** | **Estimate** | **95% CI** | **Estimate** | **95% CI** | **Estimate** | **95% CI** |
| **45-64 yrs** | **59** | **1** | $257,759 | ($256,850, $264,162) | $303,464 | ($289,329, $324,085) | $45,705 | ($29,224, $63,054) |
|  |  | **300** | $207,674 | ($204,894, $209,039) | $232,293 | ($221,567, $242,660) | $24,619 | ($14,727, $35,302) |
|  |  | **1000** | $198,302 | ($195,430, $199,390) | $218,214 | ($208,722, $227,323) | $19,912 | ($11,526, $29,619) |
|  |  | **3000** | $190,576 | ($186,758, $190,707) | $207,047 | ($197,414, $216,073) | $16,471 | ($8,928, $27,255) |
|  | **45** | **1** | $255,072 | ($254,323, $262,422) | $305,007 | ($290,024, $326,536) | $49,935 | ($31,907, $67,544) |
|  |  | **300** | $204,989 | ($202,017, $206,326) | $230,608 | ($219,777, $241,357) | $25,620 | ($15,907, $36,967) |
|  |  | **1000** | $196,316 | ($193,258, $197,196) | $216,957 | ($207,645, $226,407) | $20,641 | ($12,538, $30,985) |
|  |  | **3000** | $188,878 | ($185,381, $189,288) | $206,614 | ($196,962, $215,826) | $17,736 | ($10,005, $28,417) |
|  | **30** | **1** | $253,221 | ($252,536, $261,543) | $307,082 | ($291,196, $329,150) | $53,861 | ($34,508, $71,675) |
|  |  | **300** | $203,858 | ($200,784, $205,621) | $230,231 | ($219,576, $241,844) | $26,374 | ($16,839, $38,561) |
|  |  | **1000** | $196,022 | ($192,908, $197,304) | $217,131 | ($208,343, $227,392) | $21,109 | ($13,566, $32,293) |
|  |  | **3000** | $189,260 | ($186,153, $190,591) | $207,585 | ($198,951, $216,794) | $18,325 | ($10,916, $28,318) |
| **65-74 yrs** | **59** | **1** | $101,440 | ($100,063, $104,083) | $123,292 | ($114,953, $133,647) | $21,852 | ($13,050, $31,003) |
|  |  | **300** | $85,990 | ($83,764, $86,860) | $99,065 | ($89,580, $106,776) | $13,074 | ($4,335, $21,100) |
|  |  | **1000** | $82,175 | ($79,261, $82,928) | $92,986 | ($83,930, $100,174) | $10,811 | ($2,845, $18,455) |
|  |  | **3000** | $79,108 | ($74,266, $79,120) | $88,088 | ($78,153, $93,773) | $8,980 | ($1,424, $16,509) |
|  | **45** | **1** | $100,864 | ($99,209, $103,966) | $125,136 | ($115,896, $136,414) | $24,272 | ($14,458, $34,275) |
|  |  | **300** | $90,023 | ($87,231, $90,852) | $104,326 | ($93,658, $112,729) | $14,303 | ($4,320, $23,150) |
|  |  | **1000** | $87,693 | ($83,888, $88,197) | $100,063 | ($89,369, $107,867) | $12,370 | ($3,117, $21,030) |
|  |  | **3000** | $85,457 | ($81,097, $85,211) | $96,648 | ($84,844, $103,490) | $11,191 | ($1,694, $19,903) |
|  | **30** | **1** | $101,655 | ($99,924, $104,980) | $128,313 | ($118,268, $140,418) | $26,658 | ($15,870, $37,522) |
|  |  | **300** | $97,264 | ($93,771, $98,444) | $112,468 | ($100,671, $121,475) | $15,204 | ($4,422, $24,616) |
|  |  | **1000** | $97,404 | ($92,612, $98,314) | $111,025 | ($98,999, $119,079) | $13,622 | ($3,387, $22,707) |
|  |  | **3000** | $97,683 | ($93,304, $99,096) | $109,727 | ($97,462, $117,956) | $12,044 | ($1,620, $21,652) |
| **75-84 yrs** | **59** | **1** | $50,754 | ($50,106, $52,141) | $62,353 | ($57,738, $67,731) | $11,600 | ($6,826, $16,363) |
|  |  | **300** | $36,829 | ($35,764, $37,302) | $43,398 | ($38,777, $47,463) | $6,569 | ($2,318, $10,729) |
|  |  | **1000** | $33,637 | ($32,103, $33,722) | $39,172 | ($34,425, $42,602) | $5,535 | ($1,641, $9,504) |
|  |  | **3000** | $30,699 | ($28,935, $30,770) | $34,833 | ($30,896, $40,377) | $4,134 | ($1,069, $10,322) |
|  | **45** | **1** | $49,622 | ($48,341, $51,177) | $63,253 | ($57,440, $69,360) | $13,631 | ($7,930, $19,345) |
|  |  | **300** | $36,752 | ($35,554, $37,287) | $45,349 | ($38,910, $51,160) | $8,598 | ($2,454, $14,478) |
|  |  | **1000** | $33,727 | ($32,103, $33,999) | $41,090 | ($34,479, $46,513) | $7,363 | ($1,571, $13,217) |
|  |  | **3000** | $31,252 | ($28,866, $31,581) | $36,806 | ($30,847, $45,178) | $5,554 | ($656, $14,688) |
|  | **30** | **1** | $49,330 | ($47,377, $51,015) | $64,996 | ($58,153, $72,110) | $15,666 | ($8,975, $22,746) |
|  |  | **300** | $39,762 | ($38,396, $40,421) | $49,975 | ($41,679, $57,456) | $10,213 | ($2,303, $17,838) |
|  |  | **1000** | $38,710 | ($35,891, $39,460) | $47,209 | ($38,629, $54,492) | $8,499 | ($842, $16,263) |
|  |  | **3000** | $38,788 | ($33,504, $39,841) | $44,664 | ($36,217, $54,063) | $5,876 | (-$819, $16,413) |

Abbreviations: QALY = quality-adjusted life year, eGFR = estimated glomerular filtration rate, UACR = urine albumin to creatinine ratio, ICER = incremental cost-effectiveness ratio, CI = confidence interval

* Estimated glomerular filtration rate units in mL/min/1.73 m^2^

† Urine albumin to creatinine ratio units in mg/g
