# Supplementary material for: Cost-effectiveness of multidisciplinary care in mild to moderate chronic kidney disease in the United States: A modeling study
Source: PLoS Med. 2018 Mar 27;15(3):e1002532. doi: 10.1371/journal.pmed.1002532 (PMC5870947; doi:10.1371/journal.pmed.1002532)
Supplement: S4 Table — (DOCX) [file pmed.1002532.s006.docx]

**S4 Table: Quality-Adjusted Life Years under Multi-Disciplinary Care and Usual Care, by Age**

| **Characteristic** | | | **Control** | | **MDC** | | **Change** | |
| --- | --- | --- | --- | --- | --- | --- | --- | --- |
| **Age (yrs)** | **eGFR *** | **UACR †** | **Estimate** | **95% CI** | **Estimate** | **95% CI** | **Estimate** | **95% CI** |
| **45-64 yrs** | **59** | **1** | 6.25 | (5.41, 7.02) | 6.88 | (5.89, 7.80) | 0.63 | (0.26, 1.01) |
|  |  | **300** | 4.51 | (3.78, 5.11) | 4.90 | (4.12, 5.60) | 0.39 | (0.18, 0.66) |
|  |  | **1000** | 4.23 | (3.56, 4.84) | 4.54 | (3.81, 5.24) | 0.31 | (0.14, 0.53) |
|  |  | **3000** | 4.08 | (3.36, 4.60) | 4.34 | (3.59, 4.93) | 0.26 | (0.11, 0.48) |
|  | **45** | **1** | 6.02 | (5.25, 6.81) | 6.72 | (5.78, 7.68) | 0.70 | (0.29, 1.09) |
|  |  | **300** | 4.13 | (3.42, 4.71) | 4.55 | (3.78, 5.25) | 0.42 | (0.19, 0.72) |
|  |  | **1000** | 3.82 | (3.16, 4.40) | 4.16 | (3.44, 4.85) | 0.33 | (0.15, 0.59) |
|  |  | **3000** | 3.62 | (2.94, 4.15) | 3.91 | (3.19, 4.52) | 0.30 | (0.13, 0.53) |
|  | **30** | **1** | 5.81 | (5.09, 6.61) | 6.57 | (5.66, 7.55) | 0.76 | (0.32, 1.16) |
|  |  | **300** | 3.75 | (3.04, 4.32) | 4.19 | (3.43, 4.90) | 0.45 | (0.20, 0.78) |
|  |  | **1000** | 3.41 | (2.72, 3.96) | 3.76 | (3.03, 4.46) | 0.35 | (0.17, 0.66) |
|  |  | **3000** | 3.11 | (2.44, 3.68) | 3.44 | (2.73, 4.10) | 0.33 | (0.14, 0.58) |
| **65-74 yrs** | **59** | **1** | 3.46 | (3.01, 3.89) | 3.84 | (3.30, 4.40) | 0.39 | (0.13, 0.65) |
|  |  | **300** | 2.41 | (2.05, 2.70) | 2.69 | (2.26, 3.07) | 0.28 | (0.10, 0.47) |
|  |  | **1000** | 2.22 | (1.88, 2.49) | 2.44 | (2.05, 2.79) | 0.22 | (0.07, 0.39) |
|  |  | **3000** | 2.07 | (1.75, 2.33) | 2.24 | (1.87, 2.55) | 0.17 | (0.05, 0.34) |
|  | **45** | **1** | 3.29 | (2.87, 3.71) | 3.73 | (3.19, 4.30) | 0.44 | (0.15, 0.73) |
|  |  | **300** | 2.17 | (1.83, 2.44) | 2.49 | (2.07, 2.87) | 0.32 | (0.12, 0.54) |
|  |  | **1000** | 1.95 | (1.63, 2.21) | 2.21 | (1.83, 2.56) | 0.26 | (0.09, 0.45) |
|  |  | **3000** | 1.78 | (1.47, 2.01) | 1.99 | (1.63, 2.31) | 0.21 | (0.07, 0.40) |
|  | **30** | **1** | 3.14 | (2.75, 3.56) | 3.63 | (3.10, 4.21) | 0.49 | (0.18, 0.80) |
|  |  | **300** | 1.93 | (1.60, 2.19) | 2.28 | (1.87, 2.67) | 0.35 | (0.14, 0.59) |
|  |  | **1000** | 1.69 | (1.37, 1.93) | 1.98 | (1.60, 2.33) | 0.28 | (0.11, 0.50) |
|  |  | **3000** | 1.49 | (1.19, 1.72) | 1.71 | (1.37, 2.05) | 0.22 | (0.08, 0.44) |
| **75-84 yrs** | **59** | **1** | 1.95 | (1.71, 2.20) | 2.17 | (1.87, 2.49) | 0.22 | (0.06, 0.36) |
|  |  | **300** | 1.29 | (1.10, 1.43) | 1.43 | (1.20, 1.62) | 0.14 | (0.04, 0.26) |
|  |  | **1000** | 1.16 | (0.98, 1.28) | 1.28 | (1.06, 1.43) | 0.12 | (0.03, 0.22) |
|  |  | **3000** | 1.04 | (0.87, 1.15) | 1.13 | (0.94, 1.31) | 0.09 | (0.02, 0.25) |
|  | **45** | **1** | 1.81 | (1.58, 2.04) | 2.08 | (1.77, 2.40) | 0.26 | (0.08, 0.44) |
|  |  | **300** | 1.09 | (0.93, 1.22) | 1.29 | (1.05, 1.49) | 0.20 | (0.05, 0.35) |
|  |  | **1000** | 0.95 | (0.80, 1.05) | 1.11 | (0.90, 1.29) | 0.16 | (0.04, 0.30) |
|  |  | **3000** | 0.83 | (0.69, 0.93) | 0.95 | (0.77, 1.18) | 0.12 | (0.03, 0.33) |
|  | **30** | **1** | 1.68 | (1.46, 1.90) | 2.00 | (1.68, 2.34) | 0.31 | (0.10, 0.53) |
|  |  | **300** | 0.91 | (0.76, 1.03) | 1.15 | (0.92, 1.38) | 0.24 | (0.07, 0.42) |
|  |  | **1000** | 0.76 | (0.63, 0.88) | 0.95 | (0.75, 1.17) | 0.19 | (0.05, 0.36) |
|  |  | **3000** | 0.66 | (0.53, 0.77) | 0.79 | (0.62, 1.05) | 0.13 | (0.03, 0.37) |

Abbreviations: QALY = quality-adjusted life year, eGFR = estimated glomerular filtration rate, UACR = urine albumin to creatinine ratio, ICER = incremental cost-effectiveness ratio, CI = confidence interval

* Estimated glomerular filtration rate units in mL/min/1.73 m^2^

† Urine albumin to creatinine ratio units in mg/g
