# Supplementary material for: Cost-effectiveness of multidisciplinary care in mild to moderate chronic kidney disease in the United States: A modeling study
Source: PLoS Med. 2018 Mar 27;15(3):e1002532. doi: 10.1371/journal.pmed.1002532 (PMC5870947; doi:10.1371/journal.pmed.1002532)
Supplement: S5 Table — (DOCX) [file pmed.1002532.s007.docx]

**S5 Table: Costs under Multi-Disciplinary Care and Usual Care, by Sex**

| **Characteristic** | | | **Control** | | **MDC** | | **Change** | |
| --- | --- | --- | --- | --- | --- | --- | --- | --- |
| **Sex** | **eGFR *** | **UACR †** | **Estimate** | **95% CI** | **Estimate** | **95% CI** | **Estimate** | **95% CI** |
| **Female** | **59** | **1** | $83,575 | ($82,518, $85,596) | $101,001 | ($94,727, $108,302) | $17,426 | ($10,844, $23,827) |
|  |  | **300** | $68,242 | ($66,820, $68,976) | $78,533 | ($72,132, $84,340) | $10,292 | ($4,286, $16,101) |
|  |  | **1000** | $64,918 | ($62,719, $65,214) | $73,651 | ($67,002, $78,644) | $8,734 | ($3,152, $14,215) |
|  |  | **3000** | $61,808 | ($58,653, $61,781) | $68,984 | ($62,209, $73,576) | $7,177 | ($2,148, $12,975) |
|  | **45** | **1** | $82,960 | ($81,318, $85,183) | $102,562 | ($95,329, $110,613) | $19,602 | ($12,189, $26,888) |
|  |  | **300** | $69,956 | ($68,325, $70,627) | $81,863 | ($73,961, $88,776) | $11,907 | ($4,536, $18,947) |
|  |  | **1000** | $67,281 | ($64,739, $67,506) | $77,664 | ($69,537, $83,783) | $10,383 | ($3,331, $17,124) |
|  |  | **3000** | $64,703 | ($61,425, $64,712) | $73,684 | ($65,257, $79,660) | $8,981 | ($2,202, $16,202) |
|  | **30** | **1** | $86,142 | ($84,184, $88,563) | $108,428 | ($100,273, $117,708) | $22,286 | ($13,872, $30,781) |
|  |  | **300** | $76,243 | ($74,232, $77,052) | $89,685 | ($80,478, $97,562) | $13,441 | ($4,875, $21,531) |
|  |  | **1000** | $75,199 | ($71,623, $75,903) | $86,959 | ($77,506, $94,257) | $11,760 | ($3,558, $19,790) |
|  |  | **3000** | $74,671 | ($69,135, $75,381) | $84,684 | ($74,816, $91,731) | $10,013 | ($1,999, $18,310) |
| **Male** | **59** | **1** | $81,940 | ($81,246, $84,186) | $99,351 | ($92,784, $107,955) | $17,411 | ($10,242, $24,964) |
|  |  | **300** | $62,575 | ($61,094, $62,950) | $72,219 | ($65,695, $77,488) | $9,644 | ($3,768, $15,211) |
|  |  | **1000** | $58,145 | ($56,536, $58,278) | $65,960 | ($59,952, $70,780) | $7,815 | ($2,605, $13,139) |
|  |  | **3000** | $54,514 | ($52,226, $54,403) | $60,499 | ($54,976, $67,784) | $5,985 | ($1,620, $14,152) |
|  | **45** | **1** | $80,374 | ($79,156, $83,036) | $100,258 | ($92,448, $109,855) | $19,884 | ($11,504, $28,331) |
|  |  | **300** | $62,680 | ($61,032, $63,155) | $74,128 | ($66,116, $80,950) | $11,447 | ($4,000, $18,487) |
|  |  | **1000** | $58,789 | ($56,944, $59,066) | $68,269 | ($60,573, $74,928) | $9,481 | ($2,676, $16,609) |
|  |  | **3000** | $55,733 | ($53,012, $55,868) | $63,193 | ($55,828, $73,221) | $7,460 | ($1,350, $18,382) |
|  | **30** | **1** | $82,370 | ($80,619, $85,355) | $105,241 | ($96,076, $115,889) | $22,871 | ($13,089, $32,639) |
|  |  | **300** | $68,058 | ($66,230, $68,805) | $81,054 | ($71,789, $89,163) | $12,996 | ($4,234, $21,489) |
|  |  | **1000** | $66,312 | ($63,659, $66,992) | $76,906 | ($67,687, $84,933) | $10,594 | ($2,380, $19,206) |
|  |  | **3000** | $65,766 | ($62,616, $66,856) | $73,368 | ($64,897, $84,762) | $7,602 | ($346, $19,937) |

Abbreviations: QALY = quality-adjusted life year, eGFR = estimated glomerular filtration rate, UACR = urine albumin to creatinine ratio, ICER = incremental cost-effectiveness ratio, CI = confidence interval

* Estimated glomerular filtration rate units in mL/min/1.73 m^2^

† Urine albumin to creatinine ratio units in mg/g
