# Supplementary material for: Cost-effectiveness of multidisciplinary care in mild to moderate chronic kidney disease in the United States: A modeling study
Source: PLoS Med. 2018 Mar 27;15(3):e1002532. doi: 10.1371/journal.pmed.1002532 (PMC5870947; doi:10.1371/journal.pmed.1002532)
Supplement: S6 Table — (DOCX) [file pmed.1002532.s008.docx]

**S6 Table: Quality-Adjusted Life Years under Multi-Disciplinary Care and Usual Care, by Sex**

| **Characteristic** | | | **Control** | | **MDC** | | **Change** | |
| --- | --- | --- | --- | --- | --- | --- | --- | --- |
| **Sex** | **eGFR *** | **UACR †** | **Estimate** | **95% CI** | **Estimate** | **95% CI** | **Estimate** | **95% CI** |
| **Female** | **59** | **1** | 2.85 | (2.49, 3.22) | 3.15 | (2.71, 3.60) | 0.30 | (0.10, 0.49) |
|  |  | **300** | 2.00 | (1.70, 2.23) | 2.21 | (1.86, 2.52) | 0.22 | (0.08, 0.37) |
|  |  | **1000** | 1.84 | (1.55, 2.06) | 2.01 | (1.69, 2.28) | 0.18 | (0.06, 0.31) |
|  |  | **3000** | 1.71 | (1.43, 1.91) | 1.85 | (1.53, 2.09) | 0.14 | (0.04, 0.28) |
|  | **45** | **1** | 2.71 | (2.37, 3.06) | 3.06 | (2.62, 3.50) | 0.34 | (0.12, 0.56) |
|  |  | **300** | 1.78 | (1.50, 2.00) | 2.05 | (1.70, 2.36) | 0.27 | (0.10, 0.45) |
|  |  | **1000** | 1.60 | (1.34, 1.80) | 1.82 | (1.50, 2.10) | 0.22 | (0.08, 0.38) |
|  |  | **3000** | 1.45 | (1.20, 1.63) | 1.63 | (1.33, 1.89) | 0.18 | (0.06, 0.35) |
|  | **30** | **1** | 2.63 | (2.29, 2.97) | 3.03 | (2.58, 3.50) | 0.40 | (0.14, 0.65) |
|  |  | **300** | 1.60 | (1.33, 1.82) | 1.91 | (1.56, 2.25) | 0.31 | (0.12, 0.53) |
|  |  | **1000** | 1.40 | (1.14, 1.60) | 1.65 | (1.34, 1.96) | 0.25 | (0.10, 0.45) |
|  |  | **3000** | 1.24 | (0.99, 1.43) | 1.44 | (1.15, 1.74) | 0.20 | (0.07, 0.40) |
| **Male** | **59** | **1** | 2.64 | (2.30, 2.98) | 2.95 | (2.52, 3.38) | 0.30 | (0.10, 0.51) |
|  |  | **300** | 1.78 | (1.51, 1.98) | 1.97 | (1.64, 2.23) | 0.19 | (0.06, 0.33) |
|  |  | **1000** | 1.62 | (1.37, 1.80) | 1.77 | (1.47, 2.01) | 0.15 | (0.04, 0.28) |
|  |  | **3000** | 1.49 | (1.24, 1.65) | 1.60 | (1.34, 1.85) | 0.11 | (0.03, 0.31) |
|  | **45** | **1** | 2.48 | (2.16, 2.80) | 2.84 | (2.42, 3.28) | 0.36 | (0.12, 0.60) |
|  |  | **300** | 1.54 | (1.29, 1.73) | 1.78 | (1.46, 2.05) | 0.24 | (0.08, 0.41) |
|  |  | **1000** | 1.36 | (1.14, 1.54) | 1.55 | (1.27, 1.80) | 0.19 | (0.06, 0.35) |
|  |  | **3000** | 1.23 | (1.01, 1.39) | 1.37 | (1.12, 1.66) | 0.15 | (0.04, 0.39) |
|  | **30** | **1** | 2.37 | (2.07, 2.69) | 2.79 | (2.36, 3.26) | 0.42 | (0.15, 0.69) |
|  |  | **300** | 1.35 | (1.11, 1.54) | 1.62 | (1.31, 1.92) | 0.27 | (0.10, 0.48) |
|  |  | **1000** | 1.17 | (0.94, 1.34) | 1.38 | (1.09, 1.65) | 0.21 | (0.07, 0.41) |
|  |  | **3000** | 1.04 | (0.82, 1.20) | 1.19 | (0.94, 1.50) | 0.15 | (0.04, 0.41) |

Abbreviations: QALY = quality-adjusted life year, eGFR = estimated glomerular filtration rate, UACR = urine albumin to creatinine ratio, ICER = incremental cost-effectiveness ratio, CI = confidence interval

* Estimated glomerular filtration rate units in mL/min/1.73 m^2^

† Urine albumin to creatinine ratio units in mg/g
