# Supplementary material for: Cost-effectiveness of multidisciplinary care in mild to moderate chronic kidney disease in the United States: A modeling study
Source: PLoS Med. 2018 Mar 27;15(3):e1002532. doi: 10.1371/journal.pmed.1002532 (PMC5870947; doi:10.1371/journal.pmed.1002532)
Supplement: S7 Table — (DOCX) [file pmed.1002532.s009.docx]

**S7 Table: Costs under Multi-Disciplinary Care and Usual Care, by Race**

| **Characteristic** | | | **Control** | | **MDC** | | **Change** | |
| --- | --- | --- | --- | --- | --- | --- | --- | --- |
| **Race** | **eGFR *** | **UACR †** | **Estimate** | **95% CI** | **Estimate** | **95% CI** | **Estimate** | **95% CI** |
| **White** | **59** | **1** | $78,240 | ($77,521, $79,999) | $94,962 | ($88,910, $102,266) | $16,722 | ($10,145, $23,124) |
|  |  | **300** | $61,936 | ($60,590, $62,359) | $71,616 | ($65,472, $76,676) | $9,680 | ($4,030, $14,945) |
|  |  | **1000** | $58,192 | ($56,452, $58,341) | $66,295 | ($60,225, $70,585) | $8,103 | ($2,938, $12,939) |
|  |  | **3000** | $54,973 | ($52,554, $54,841) | $61,419 | ($55,581, $66,441) | $6,446 | ($1,938, $12,360) |
|  | **45** | **1** | $77,359 | ($76,116, $79,356) | $96,278 | ($89,230, $104,389) | $18,919 | ($11,457, $26,290) |
|  |  | **300** | $62,841 | ($61,209, $63,289) | $74,266 | ($66,597, $80,554) | $11,425 | ($4,333, $18,028) |
|  |  | **1000** | $59,621 | ($57,738, $59,829) | $69,350 | ($61,791, $75,175) | $9,729 | ($3,158, $16,111) |
|  |  | **3000** | $56,907 | ($54,347, $56,863) | $64,994 | ($57,489, $71,830) | $8,087 | ($1,907, $15,867) |
|  | **30** | **1** | $79,676 | ($77,951, $81,901) | $101,144 | ($93,096, $110,239) | $21,468 | ($13,079, $30,045) |
|  |  | **300** | $68,298 | ($66,510, $68,925) | $81,336 | ($72,568, $88,744) | $13,038 | ($4,824, $20,764) |
|  |  | **1000** | $66,875 | ($64,284, $67,475) | $77,933 | ($69,065, $84,793) | $11,058 | ($3,351, $18,634) |
|  |  | **3000** | $66,290 | ($62,001, $67,090) | $75,131 | ($66,683, $83,004) | $8,842 | ($1,677, $17,462) |
| **Black** | **59** | **1** | $103,078 | ($102,570, $105,582) | $123,813 | ($116,178, $134,627) | $20,735 | ($12,267, $30,300) |
|  |  | **300** | $79,399 | ($77,907, $79,944) | $90,834 | ($83,172, $97,633) | $11,435 | ($4,258, $18,393) |
|  |  | **1000** | $74,559 | ($72,655, $74,667) | $83,678 | ($76,581, $90,395) | $9,119 | ($3,024, $16,525) |
|  |  | **3000** | $70,234 | ($67,364, $69,932) | $77,449 | ($70,438, $86,608) | $7,215 | ($1,749, $17,918) |
|  | **45** | **1** | $100,709 | ($100,271, $103,918) | $124,467 | ($115,608, $136,413) | $23,758 | ($13,570, $34,052) |
|  |  | **300** | $79,571 | ($77,861, $80,122) | $92,676 | ($83,343, $101,061) | $13,105 | ($4,238, $21,773) |
|  |  | **1000** | $75,508 | ($73,126, $75,613) | $86,437 | ($77,265, $94,791) | $10,930 | ($2,918, $20,335) |
|  |  | **3000** | $71,901 | ($68,592, $71,799) | $80,731 | ($71,528, $92,997) | $8,830 | ($1,423, $22,664) |
|  | **30** | **1** | $102,172 | ($101,537, $106,052) | $129,407 | ($119,000, $142,517) | $27,236 | ($15,352, $38,226) |
|  |  | **300** | $85,616 | ($83,044, $86,526) | $100,081 | ($89,212, $109,915) | $14,465 | ($4,182, $24,622) |
|  |  | **1000** | $83,818 | ($80,349, $84,545) | $95,829 | ($84,914, $105,610) | $12,011 | ($2,308, $22,681) |
|  |  | **3000** | $83,039 | ($76,734, $83,776) | $92,164 | ($80,575, $105,421) | $9,124 | ($164, $23,901) |
| **Other** | **59** | **1** | $90,205 | ($88,952, $92,438) | $108,335 | ($101,356, $116,448) | $18,130 | ($10,966, $25,383) |
|  |  | **300** | $74,619 | ($72,584, $75,661) | $84,479 | ($77,363, $91,066) | $9,860 | ($3,328, $16,417) |
|  |  | **1000** | $71,262 | ($68,598, $71,812) | $79,446 | ($72,353, $85,623) | $8,184 | ($2,238, $15,086) |
|  |  | **3000** | $68,258 | ($65,046, $68,544) | $74,759 | ($67,669, $82,693) | $6,501 | ($1,177, $15,916) |
|  | **45** | **1** | $89,462 | ($87,865, $91,964) | $109,849 | ($101,828, $118,816) | $20,387 | ($12,156, $28,556) |
|  |  | **300** | $77,231 | ($74,888, $78,462) | $88,466 | ($79,677, $96,633) | $11,235 | ($3,041, $19,370) |
|  |  | **1000** | $74,921 | ($71,712, $75,635) | $84,798 | ($75,549, $92,628) | $9,876 | ($1,856, $18,566) |
|  |  | **3000** | $72,712 | ($69,287, $73,322) | $80,837 | ($71,704, $91,811) | $8,124 | ($676, $20,386) |
|  | **30** | **1** | $88,649 | ($86,442, $91,351) | $110,977 | ($101,999, $120,674) | $22,328 | ($13,222, $31,339) |
|  |  | **300** | $81,733 | ($78,708, $83,417) | $93,820 | ($83,400, $103,081) | $12,087 | ($2,439, $21,438) |
|  |  | **1000** | $81,934 | ($77,084, $83,542) | $92,618 | ($81,438, $101,878) | $10,684 | ($952, $20,657) |
|  |  | **3000** | $82,550 | ($77,182, $84,571) | $90,977 | ($80,437, $103,708) | $8,427 | (-$840, $22,023) |

Abbreviations: QALY = quality-adjusted life year, eGFR = estimated glomerular filtration rate, UACR = urine albumin to creatinine ratio, ICER = incremental cost-effectiveness ratio, CI = confidence interval

* Estimated glomerular filtration rate units in mL/min/1.73 m^2^

† Urine albumin to creatinine ratio units in mg/g
