# Supplementary material for: Cost-effectiveness of multidisciplinary care in mild to moderate chronic kidney disease in the United States: A modeling study
Source: PLoS Med. 2018 Mar 27;15(3):e1002532. doi: 10.1371/journal.pmed.1002532 (PMC5870947; doi:10.1371/journal.pmed.1002532)
Supplement: S8 Table — (DOCX) [file pmed.1002532.s010.docx]

**S8 Table: Quality-Adjusted Life Years under Multi-Disciplinary Care and Usual Care, by Race**

| **Characteristic** | | | **Control** | | **MDC** | | **Change** | |
| --- | --- | --- | --- | --- | --- | --- | --- | --- |
| **Race** | **eGFR *** | **UACR †** | **Estimate** | **95% CI** | **Estimate** | **95% CI** | **Estimate** | **95% CI** |
| **White** | **59** | **1** | 2.68 | (2.34, 3.02) | 2.97 | (2.56, 3.40) | 0.29 | (0.10, 0.48) |
|  |  | **300** | 1.84 | (1.57, 2.06) | 2.05 | (1.71, 2.32) | 0.20 | (0.07, 0.34) |
|  |  | **1000** | 1.69 | (1.43, 1.88) | 1.85 | (1.55, 2.09) | 0.16 | (0.05, 0.29) |
|  |  | **3000** | 1.56 | (1.31, 1.73) | 1.68 | (1.40, 1.91) | 0.12 | (0.04, 0.28) |
|  | **45** | **1** | 2.53 | (2.21, 2.86) | 2.87 | (2.46, 3.31) | 0.34 | (0.12, 0.56) |
|  |  | **300** | 1.62 | (1.37, 1.82) | 1.87 | (1.55, 2.15) | 0.25 | (0.09, 0.42) |
|  |  | **1000** | 1.44 | (1.21, 1.62) | 1.64 | (1.35, 1.90) | 0.20 | (0.07, 0.36) |
|  |  | **3000** | 1.30 | (1.08, 1.46) | 1.46 | (1.20, 1.71) | 0.16 | (0.05, 0.35) |
|  | **30** | **1** | 2.45 | (2.14, 2.77) | 2.84 | (2.42, 3.30) | 0.39 | (0.14, 0.65) |
|  |  | **300** | 1.44 | (1.19, 1.63) | 1.73 | (1.41, 2.03) | 0.29 | (0.11, 0.50) |
|  |  | **1000** | 1.24 | (1.02, 1.42) | 1.48 | (1.19, 1.75) | 0.23 | (0.09, 0.42) |
|  |  | **3000** | 1.09 | (0.88, 1.26) | 1.27 | (1.01, 1.55) | 0.18 | (0.06, 0.39) |
| **Black** | **59** | **1** | 3.02 | (2.62, 3.38) | 3.36 | (2.88, 3.84) | 0.34 | (0.12, 0.58) |
|  |  | **300** | 2.03 | (1.72, 2.27) | 2.25 | (1.88, 2.56) | 0.21 | (0.07, 0.37) |
|  |  | **1000** | 1.86 | (1.57, 2.09) | 2.02 | (1.70, 2.32) | 0.16 | (0.05, 0.33) |
|  |  | **3000** | 1.72 | (1.43, 1.91) | 1.85 | (1.54, 2.15) | 0.13 | (0.04, 0.36) |
|  | **45** | **1** | 2.83 | (2.47, 3.19) | 3.23 | (2.76, 3.73) | 0.40 | (0.14, 0.67) |
|  |  | **300** | 1.79 | (1.50, 2.02) | 2.05 | (1.69, 2.38) | 0.26 | (0.08, 0.45) |
|  |  | **1000** | 1.61 | (1.34, 1.82) | 1.81 | (1.49, 2.12) | 0.20 | (0.06, 0.41) |
|  |  | **3000** | 1.47 | (1.20, 1.66) | 1.63 | (1.33, 1.97) | 0.16 | (0.04, 0.45) |
|  | **30** | **1** | 2.72 | (2.38, 3.09) | 3.18 | (2.71, 3.71) | 0.47 | (0.16, 0.76) |
|  |  | **300** | 1.61 | (1.33, 1.85) | 1.91 | (1.55, 2.26) | 0.29 | (0.10, 0.52) |
|  |  | **1000** | 1.43 | (1.15, 1.65) | 1.66 | (1.32, 1.99) | 0.22 | (0.07, 0.46) |
|  |  | **3000** | 1.29 | (1.01, 1.50) | 1.46 | (1.14, 1.83) | 0.16 | (0.04, 0.47) |
| **Other** | **59** | **1** | 2.91 | (2.53, 3.28) | 3.22 | (2.77, 3.68) | 0.31 | (0.10, 0.51) |
|  |  | **300** | 2.04 | (1.73, 2.29) | 2.25 | (1.88, 2.56) | 0.20 | (0.06, 0.36) |
|  |  | **1000** | 1.89 | (1.59, 2.12) | 2.05 | (1.71, 2.35) | 0.16 | (0.05, 0.32) |
|  |  | **3000** | 1.76 | (1.47, 1.97) | 1.89 | (1.57, 2.18) | 0.13 | (0.03, 0.35) |
|  | **45** | **1** | 2.76 | (2.40, 3.11) | 3.12 | (2.67, 3.58) | 0.36 | (0.12, 0.59) |
|  |  | **300** | 1.83 | (1.53, 2.07) | 2.07 | (1.71, 2.40) | 0.24 | (0.08, 0.43) |
|  |  | **1000** | 1.66 | (1.38, 1.88) | 1.86 | (1.53, 2.17) | 0.20 | (0.06, 0.39) |
|  |  | **3000** | 1.53 | (1.25, 1.73) | 1.69 | (1.37, 2.01) | 0.16 | (0.04, 0.43) |
|  | **30** | **1** | 2.59 | (2.25, 2.92) | 2.99 | (2.54, 3.46) | 0.40 | (0.14, 0.66) |
|  |  | **300** | 1.60 | (1.32, 1.84) | 1.88 | (1.53, 2.23) | 0.27 | (0.09, 0.48) |
|  |  | **1000** | 1.43 | (1.16, 1.66) | 1.65 | (1.32, 1.99) | 0.22 | (0.07, 0.44) |
|  |  | **3000** | 1.31 | (1.03, 1.52) | 1.47 | (1.16, 1.82) | 0.16 | (0.04, 0.45) |

Abbreviations: QALY = quality-adjusted life year, eGFR = estimated glomerular filtration rate, UACR = urine albumin to creatinine ratio, ICER = incremental cost-effectiveness ratio, CI = confidence interval

* Estimated glomerular filtration rate units in mL/min/1.73 m^2^

† Urine albumin to creatinine ratio units in mg/g
