# Supplementary material for: Cost-effectiveness of multidisciplinary care in mild to moderate chronic kidney disease in the United States: A modeling study
Source: PLoS Med. 2018 Mar 27;15(3):e1002532. doi: 10.1371/journal.pmed.1002532 (PMC5870947; doi:10.1371/journal.pmed.1002532)
Supplement: S9 Table — (DOCX) [file pmed.1002532.s011.docx]

**S9 Table: Cost-Effectiveness when Varying the Effectiveness of Multi-Disciplinary Care, Patients with eGFR of 59 mL/min/1.73 m^2^**

| **UACR *** | **Scenario** | **HR Death** | | **HR ESRD** | | **ICER ($/QALY)** | **Net Monetary Benefit ($) †** | |
| --- | --- | --- | --- | --- | --- | --- | --- | --- |
|  |  | **Estimate** | **95% CI** | **Estimate** | **95% CI** |  | **Estimate** | **95% CI** |
| **1** | **Base Case** | 0.70 | (0.56, 0.89) | 0.55 | (0.36, 0.85) | $57,958 | $27,662 | ($4,404, $50,748) |
|  | **50% of Base Case** | 0.85 | (0.78, 0.95) | 0.78 | (0.69, 0.92) | $89,293 | $8,208 | (-$1,998, $17,097) |
|  | **25% of Base Case** | 0.93 | (0.89, 0.97) | 0.89 | (0.84, 0.96) | $151,869 | -$120 | (-$4,991, $3,724) |
|  | **100% of Non-Discounted** | 0.68 | (0.54, 0.88) | 0.55 | (0.36, 0.84) | $54,451 | $33,121 | ($5,972, $59,596) |
|  | **50% of Non-Discounted** | 0.84 | (0.77, 0.94) | 0.78 | (0.68, 0.92) | $81,419 | $10,738 | (-$1,218, $21,029) |
|  | **25% of Non-Discounted** | 0.92 | (0.88, 0.97) | 0.89 | (0.84, 0.96) | $135,250 | $1,102 | (-$4,610, $5,596) |
|  | **Only Mortality, Base Case** | 0.73 | (0.59, 0.91) | 1.02 | (1.00, 1.03) | $65,465 | $22,458 | ($666, $44,615) |
|  | **Only Mortality, 50% of Base Case** | 0.86 | (0.79, 0.96) | 1.01 | (1.00, 1.02) | $99,780 | $6,102 | (-$3,694, $14,835) |
|  | **Only Mortality, 25% of Base Case** | 0.93 | (0.90, 0.98) | 1.00 | (1.00, 1.01) | $168,427 | -$1,074 | (-$5,813, $2,766) |
| **300** | **Base Case** | 0.78 | (0.65, 0.92) | 0.60 | (0.41, 0.86) | $49,116 | $20,458 | ($5,420, $37,581) |
|  | **50% of Base Case** | 0.90 | (0.85, 0.96) | 0.82 | (0.74, 0.94) | $76,187 | $6,442 | ($429, $11,917) |
|  | **25% of Base Case** | 0.95 | (0.93, 0.98) | 0.91 | (0.88, 0.97) | $126,465 | $966 | (-$1,764, $3,336) |
|  | **100% of Non-Discounted** | 0.72 | (0.57, 0.90) | 0.57 | (0.39, 0.85) | $44,089 | $30,930 | ($8,853, $55,094) |
|  | **50% of Non-Discounted** | 0.87 | (0.80, 0.95) | 0.80 | (0.72, 0.93) | $62,461 | $11,194 | ($2,073, $19,445) |
|  | **25% of Non-Discounted** | 0.93 | (0.90, 0.98) | 0.90 | (0.86, 0.96) | $96,402 | $3,244 | (-$1,003, $6,904) |
|  | **Only Mortality, Base Case** | 0.84 | (0.73, 0.96) | 1.05 | (1.01, 1.16) | $79,474 | $9,877 | (-$703, $20,880) |
|  | **Only Mortality, 50% of Base Case** | 0.92 | (0.87, 0.98) | 1.03 | (1.01, 1.06) | $109,766 | $2,653 | (-$2,314, $7,449) |
|  | **Only Mortality, 25% of Base Case** | 0.96 | (0.94, 0.99) | 1.01 | (1.00, 1.03) | $171,154 | -$678 | (-$3,090, $1,544) |
| **1000** | **Base Case** | 0.82 | (0.69, 0.95) | 0.66 | (0.48, 0.92) | $50,916 | $16,081 | ($4,047, $31,005) |
|  | **50% of Base Case** | 0.92 | (0.87, 0.98) | 0.85 | (0.79, 0.97) | $78,111 | $5,097 | ($224, $9,764) |
|  | **25% of Base Case** | 0.96 | (0.94, 0.99) | 0.93 | (0.90, 0.99) | $128,005 | $739 | (-$1,494, $2,823) |
|  | **100% of Non-Discounted** | 0.74 | (0.58, 0.92) | 0.61 | (0.43, 0.89) | $43,853 | $28,252 | ($8,083, $53,857) |
|  | **50% of Non-Discounted** | 0.88 | (0.80, 0.96) | 0.82 | (0.75, 0.95) | $60,116 | $10,609 | ($2,135, $19,108) |
|  | **25% of Non-Discounted** | 0.94 | (0.90, 0.98) | 0.91 | (0.88, 0.98) | $89,879 | $3,377 | (-$563, $7,188) |
|  | **Only Mortality, Base Case** | 0.86 | (0.75, 0.97) | 1.08 | (1.02, 1.22) | $82,489 | $7,985 | (-$647, $18,043) |
|  | **Only Mortality, 50% of Base Case** | 0.93 | (0.88, 0.98) | 1.04 | (1.01, 1.09) | $111,259 | $2,173 | (-$1,885, $6,518) |
|  | **Only Mortality, 25% of Base Case** | 0.97 | (0.94, 0.99) | 1.02 | (1.01, 1.04) | $169,827 | -$542 | (-$2,507, $1,510) |
| **3000** | **Base Case** | 0.87 | (0.69, 0.97) | 0.74 | (0.52, 0.93) | $52,297 | $12,269 | ($3,187, $30,994) |
|  | **50% of Base Case** | 0.93 | (0.87, 0.98) | 0.89 | (0.80, 0.98) | $78,474 | $4,163 | ($170, $9,574) |
|  | **25% of Base Case** | 0.97 | (0.93, 0.99) | 0.95 | (0.90, 0.99) | $126,870 | $651 | (-$1,259, $3,061) |
|  | **100% of Non-Discounted** | 0.76 | (0.52, 0.93) | 0.64 | (0.43, 0.87) | $41,638 | $27,074 | ($7,959, $65,406) |
|  | **50% of Non-Discounted** | 0.88 | (0.77, 0.96) | 0.82 | (0.72, 0.94) | $55,246 | $10,850 | ($2,465, $22,345) |
|  | **25% of Non-Discounted** | 0.94 | (0.89, 0.98) | 0.91 | (0.86, 0.97) | $80,218 | $3,839 | (-$151, $8,858) |
|  | **Only Mortality, Base Case** | 0.88 | (0.72, 0.97) | 1.11 | (1.02, 1.31) | $84,280 | $6,772 | (-$687, $19,869) |
|  | **Only Mortality, 50% of Base Case** | 0.94 | (0.87, 0.99) | 1.05 | (1.01, 1.14) | $111,165 | $1,907 | (-$1,640, $7,422) |
|  | **Only Mortality, 25% of Base Case** | 0.97 | (0.93, 0.99) | 1.03 | (1.01, 1.06) | $166,174 | -$388 | (-$2,126, $2,166) |

Abbreviations: eGFR = estimated glomerular filtration rate, UACR = urine albumin to creatinine ratio, ICER = incremental cost-effectiveness ratio, HR = hazard ratio, QALY = quality-adjusted life year, ESRD = end-stage renal disease, CI = confidence interval

Notes:

We summarize each of the scenarios below:

Base case: MDC effectiveness was 25% in CKD stage 3, 50% in CKD stage 4, 100% in CKD stage 5

50%, 25% of base case: MDC effectiveness was 12.5%/6.25% in CKD stage 3, 25%/12.5% in CKD stage 4, 50%/25% in CKD stage 5, respectively

100%, 50%, 25% of non-discounted: MDC effectiveness was 100%, 50%, and 25% in all CKD stages respectively

Only Mortality: Same as above except MDC was only effective in reducing mortality (not progression to ESRD)

* Urine albumin to creatinine ratio in units of mg/g

† Net monetary benefit under a willingness to pay threshold of $150,000 per QALY gained
