# Supplementary material for: Cost-effectiveness of multidisciplinary care in mild to moderate chronic kidney disease in the United States: A modeling study
Source: PLoS Med. 2018 Mar 27;15(3):e1002532. doi: 10.1371/journal.pmed.1002532 (PMC5870947; doi:10.1371/journal.pmed.1002532)
Supplement: S10 Table — (DOCX) [file pmed.1002532.s012.docx]

**S10 Table: Cost-Effectiveness when Varying the Effectiveness of Multi-Disciplinary Care, Patients with eGFR of 45 mL/min/1.73 m^2^**

| **UACR *** | **Scenario** | **HR Death** | | **HR ESRD** | | **ICER ($/QALY)** | **Net Monetary Benefit ($) †** | |
| --- | --- | --- | --- | --- | --- | --- | --- | --- |
|  |  | **Estimate** | **95% CI** | **Estimate** | **95% CI** |  | **Estimate** | **95% CI** |
| **1** | **Base Case** | 0.69 | (0.54, 0.88) | 0.55 | (0.36, 0.84) | $56,181 | $32,975 | ($5,996, $59,557) |
|  | **50% of Base Case** | 0.84 | (0.77, 0.94) | 0.78 | (0.68, 0.92) | $86,086 | $10,085 | (-$1,674, $20,273) |
|  | **25% of Base Case** | 0.92 | (0.88, 0.97) | 0.89 | (0.84, 0.96) | $145,804 | $315 | (-$5,302, $4,711) |
|  | **100% of Non-Discounted** | 0.68 | (0.53, 0.88) | 0.55 | (0.36, 0.84) | $54,692 | $35,774 | ($6,800, $64,176) |
|  | **50% of Non-Discounted** | 0.84 | (0.76, 0.94) | 0.78 | (0.68, 0.92) | $82,624 | $11,371 | (-$1,325, $22,242) |
|  | **25% of Non-Discounted** | 0.92 | (0.88, 0.97) | 0.89 | (0.84, 0.96) | $138,376 | $934 | (-$5,111, $5,657) |
|  | **Only Mortality, Base Case** | 0.72 | (0.57, 0.91) | 1.01 | (1.00, 1.01) | $64,783 | $25,913 | ($1,054, $51,133) |
|  | **Only Mortality, 50% of Base Case** | 0.86 | (0.78, 0.95) | 1.00 | (1.00, 1.01) | $98,145 | $7,211 | (-$4,003, $17,174) |
|  | **Only Mortality, 25% of Base Case** | 0.93 | (0.89, 0.98) | 1.00 | (1.00, 1.00) | $164,848 | -$990 | (-$6,410, $3,391) |
| **300** | **Base Case** | 0.75 | (0.62, 0.91) | 0.59 | (0.41, 0.85) | $46,612 | $25,888 | ($7,768, $46,848) |
|  | **50% of Base Case** | 0.88 | (0.83, 0.96) | 0.81 | (0.73, 0.93) | $70,767 | $8,480 | ($1,338, $15,127) |
|  | **25% of Base Case** | 0.94 | (0.92, 0.98) | 0.91 | (0.87, 0.96) | $114,947 | $1,760 | (-$1,496, $4,639) |
|  | **100% of Non-Discounted** | 0.72 | (0.58, 0.90) | 0.58 | (0.40, 0.84) | $45,037 | $31,208 | ($9,522, $55,856) |
|  | **50% of Non-Discounted** | 0.87 | (0.81, 0.95) | 0.80 | (0.72, 0.92) | $65,302 | $10,840 | ($2,125, $18,956) |
|  | **25% of Non-Discounted** | 0.94 | (0.91, 0.98) | 0.90 | (0.86, 0.96) | $102,133 | $2,880 | (-$1,093, $6,399) |
|  | **Only Mortality, Base Case** | 0.83 | (0.72, 0.96) | 1.02 | (1.01, 1.06) | $79,483 | $11,671 | (-$505, $24,476) |
|  | **Only Mortality, 50% of Base Case** | 0.91 | (0.86, 0.98) | 1.01 | (1.00, 1.03) | $106,919 | $3,354 | (-$2,340, $8,866) |
|  | **Only Mortality, 25% of Base Case** | 0.96 | (0.93, 0.99) | 1.01 | (1.00, 1.01) | $162,452 | -$471 | (-$3,248, $2,096) |
| **1000** | **Base Case** | 0.79 | (0.66, 0.93) | 0.64 | (0.45, 0.87) | $48,972 | $20,468 | ($6,017, $39,252) |
|  | **50% of Base Case** | 0.90 | (0.85, 0.97) | 0.83 | (0.76, 0.94) | $72,839 | $6,769 | ($1,041, $12,642) |
|  | **25% of Base Case** | 0.95 | (0.93, 0.98) | 0.92 | (0.89, 0.97) | $115,501 | $1,429 | (-$1,193, $4,011) |
|  | **100% of Non-Discounted** | 0.75 | (0.60, 0.92) | 0.62 | (0.43, 0.86) | $46,669 | $26,593 | ($8,160, $51,117) |
|  | **50% of Non-Discounted** | 0.88 | (0.82, 0.96) | 0.82 | (0.74, 0.93) | $65,457 | $9,475 | ($2,011, $17,244) |
|  | **25% of Non-Discounted** | 0.94 | (0.91, 0.98) | 0.91 | (0.87, 0.97) | $98,825 | $2,711 | (-$731, $6,133) |
|  | **Only Mortality, Base Case** | 0.85 | (0.74, 0.96) | 1.04 | (1.01, 1.10) | $83,591 | $9,369 | (-$441, $21,003) |
|  | **Only Mortality, 50% of Base Case** | 0.92 | (0.87, 0.98) | 1.02 | (1.01, 1.05) | $108,886 | $2,742 | (-$1,870, $7,766) |
|  | **Only Mortality, 25% of Base Case** | 0.96 | (0.94, 0.99) | 1.01 | (1.00, 1.02) | $160,345 | -$336 | (-$2,588, $2,023) |
| **3000** | **Base Case** | 0.84 | (0.66, 0.96) | 0.69 | (0.48, 0.89) | $50,867 | $15,986 | ($4,819, $38,698) |
|  | **50% of Base Case** | 0.92 | (0.84, 0.98) | 0.86 | (0.78, 0.95) | $73,288 | $5,619 | ($861, $12,025) |
|  | **25% of Base Case** | 0.96 | (0.92, 0.99) | 0.93 | (0.89, 0.98) | $113,357 | $1,286 | (-$950, $4,072) |
|  | **100% of Non-Discounted** | 0.78 | (0.56, 0.94) | 0.64 | (0.44, 0.85) | $46,924 | $23,364 | ($7,253, $56,041) |
|  | **50% of Non-Discounted** | 0.89 | (0.79, 0.97) | 0.83 | (0.74, 0.93) | $63,255 | $8,866 | ($1,979, $18,309) |
|  | **25% of Non-Discounted** | 0.95 | (0.90, 0.98) | 0.91 | (0.87, 0.97) | $92,257 | $2,816 | (-$408, $6,920) |
|  | **Only Mortality, Base Case** | 0.86 | (0.70, 0.97) | 1.06 | (1.01, 1.18) | $86,957 | $7,775 | (-$561, $22,705) |
|  | **Only Mortality, 50% of Base Case** | 0.93 | (0.85, 0.98) | 1.03 | (1.01, 1.08) | $109,948 | $2,344 | (-$1,607, $8,545) |
|  | **Only Mortality, 25% of Base Case** | 0.96 | (0.93, 0.99) | 1.02 | (1.00, 1.04) | $157,003 | -$200 | (-$2,137, $2,640) |

Abbreviations: eGFR = estimated glomerular filtration rate, UACR = urine albumin to creatinine ratio, ICER = incremental cost-effectiveness ratio, HR = hazard ratio, QALY = quality-adjusted life year, ESRD = end-stage renal disease, CI = confidence interval

Notes:

We summarize each of the scenarios below:

Base case: MDC effectiveness was 25% in CKD stage 3, 50% in CKD stage 4, 100% in CKD stage 5

50%, 25% of base case: MDC effectiveness was 12.5%/6.25% in CKD stage 3, 25%/12.5% in CKD stage 4, 50%/25% in CKD stage 5, respectively

100%, 50%, 25% of non-discounted: MDC effectiveness was 100%, 50%, and 25% in all CKD stages respectively

Only Mortality: Same as above except MDC was only effective in reducing mortality (not progression to ESRD)

* Urine albumin to creatinine ratio in units of mg/g

† Net monetary benefit under a willingness to pay threshold of $150,000 per QALY gained
