# Supplementary material for: Cost-effectiveness of multidisciplinary care in mild to moderate chronic kidney disease in the United States: A modeling study
Source: PLoS Med. 2018 Mar 27;15(3):e1002532. doi: 10.1371/journal.pmed.1002532 (PMC5870947; doi:10.1371/journal.pmed.1002532)
Supplement: S11 Table — (DOCX) [file pmed.1002532.s013.docx]

**S11 Table: Cost-Effectiveness when Varying the Effectiveness of Multi-Disciplinary Care, Patients with eGFR of 30 mL/min/1.73 m^2^**

| **UACR *** | **Scenario** | **HR Death** | | **HR ESRD** | | **ICER ($/QALY)** | **Net Monetary Benefit ($) †** | |
| --- | --- | --- | --- | --- | --- | --- | --- | --- |
|  |  | **Estimate** | **95% CI** | **Estimate** | **95% CI** |  | **Estimate** | **95% CI** |
| **1** | **Base Case** | 0.67 | (0.52, 0.87) | 0.56 | (0.37, 0.85) | $55,315 | $38,626 | ($7,890, $68,512) |
|  | **50% of Base Case** | 0.84 | (0.76, 0.94) | 0.78 | (0.69, 0.93) | $84,555 | $11,957 | (-$1,406, $23,339) |
|  | **25% of Base Case** | 0.92 | (0.88, 0.97) | 0.89 | (0.84, 0.96) | $142,969 | $611 | (-$5,774, $5,547) |
|  | **100% of Non-Discounted** | 0.67 | (0.52, 0.87) | 0.56 | (0.37, 0.85) | $55,315 | $38,626 | ($7,890, $68,512) |
|  | **50% of Non-Discounted** | 0.84 | (0.76, 0.94) | 0.78 | (0.69, 0.93) | $84,555 | $11,957 | (-$1,406, $23,339) |
|  | **25% of Non-Discounted** | 0.92 | (0.88, 0.97) | 0.89 | (0.84, 0.96) | $142,969 | $611 | (-$5,774, $5,547) |
|  | **Only Mortality, Base Case** | 0.71 | (0.56, 0.90) | 1.00 | (1.00, 1.01) | $65,372 | $29,002 | ($1,103, $57,614) |
|  | **Only Mortality, 50% of Base Case** | 0.85 | (0.78, 0.95) | 1.00 | (1.00, 1.00) | $98,875 | $8,011 | (-$4,545, $19,318) |
|  | **Only Mortality, 25% of Base Case** | 0.93 | (0.89, 0.98) | 1.00 | (1.00, 1.00) | $165,814 | -$1,189 | (-$7,265, $3,741) |
| **300** | **Base Case** | 0.73 | (0.60, 0.90) | 0.59 | (0.40, 0.83) | $45,337 | $30,540 | ($9,914, $55,724) |
|  | **50% of Base Case** | 0.88 | (0.82, 0.95) | 0.80 | (0.71, 0.92) | $68,746 | $9,997 | ($2,082, $17,719) |
|  | **25% of Base Case** | 0.94 | (0.91, 0.98) | 0.90 | (0.86, 0.96) | $111,067 | $2,233 | (-$1,306, $5,510) |
|  | **100% of Non-Discounted** | 0.73 | (0.60, 0.90) | 0.59 | (0.40, 0.83) | $45,337 | $30,540 | ($9,914, $55,724) |
|  | **50% of Non-Discounted** | 0.88 | (0.82, 0.95) | 0.80 | (0.71, 0.92) | $68,746 | $9,997 | ($2,082, $17,719) |
|  | **25% of Non-Discounted** | 0.94 | (0.91, 0.98) | 0.90 | (0.86, 0.96) | $111,067 | $2,233 | (-$1,306, $5,510) |
|  | **Only Mortality, Base Case** | 0.82 | (0.72, 0.95) | 1.01 | (1.00, 1.02) | $84,755 | $11,509 | (-$779, $24,896) |
|  | **Only Mortality, 50% of Base Case** | 0.91 | (0.86, 0.98) | 1.00 | (1.00, 1.01) | $112,823 | $3,082 | (-$2,656, $8,853) |
|  | **Only Mortality, 25% of Base Case** | 0.96 | (0.93, 0.99) | 1.00 | (1.00, 1.00) | $169,438 | -$782 | (-$3,586, $1,892) |
| **1000** | **Base Case** | 0.78 | (0.64, 0.92) | 0.61 | (0.42, 0.83) | $48,323 | $23,574 | ($7,867, $46,626) |
|  | **50% of Base Case** | 0.90 | (0.84, 0.96) | 0.81 | (0.73, 0.92) | $72,025 | $7,675 | ($1,672, $14,508) |
|  | **25% of Base Case** | 0.95 | (0.92, 0.98) | 0.91 | (0.87, 0.96) | $113,383 | $1,687 | (-$1,037, $4,574) |
|  | **100% of Non-Discounted** | 0.78 | (0.64, 0.92) | 0.61 | (0.42, 0.83) | $48,323 | $23,574 | ($7,867, $46,626) |
|  | **50% of Non-Discounted** | 0.90 | (0.84, 0.96) | 0.81 | (0.73, 0.92) | $72,025 | $7,675 | ($1,672, $14,508) |
|  | **25% of Non-Discounted** | 0.95 | (0.92, 0.98) | 0.91 | (0.87, 0.96) | $113,383 | $1,687 | (-$1,037, $4,574) |
|  | **Only Mortality, Base Case** | 0.84 | (0.74, 0.96) | 1.01 | (1.00, 1.05) | $92,421 | $8,371 | (-$894, $20,035) |
|  | **Only Mortality, 50% of Base Case** | 0.92 | (0.87, 0.98) | 1.00 | (1.00, 1.02) | $118,660 | $2,152 | (-$2,232, $7,143) |
|  | **Only Mortality, 25% of Base Case** | 0.96 | (0.93, 0.99) | 1.00 | (1.00, 1.01) | $171,774 | -$728 | (-$2,871, $1,567) |
| **3000** | **Base Case** | 0.83 | (0.64, 0.95) | 0.64 | (0.43, 0.84) | $50,593 | $17,416 | ($5,846, $42,925) |
|  | **50% of Base Case** | 0.92 | (0.84, 0.98) | 0.83 | (0.75, 0.92) | $74,387 | $5,832 | ($1,206, $12,270) |
|  | **25% of Base Case** | 0.96 | (0.92, 0.99) | 0.92 | (0.88, 0.96) | $115,088 | $1,274 | (-$850, $3,985) |
|  | **100% of Non-Discounted** | 0.83 | (0.64, 0.95) | 0.64 | (0.43, 0.84) | $50,593 | $17,416 | ($5,846, $42,925) |
|  | **50% of Non-Discounted** | 0.92 | (0.84, 0.98) | 0.83 | (0.75, 0.92) | $74,387 | $5,832 | ($1,206, $12,270) |
|  | **25% of Non-Discounted** | 0.96 | (0.92, 0.99) | 0.92 | (0.88, 0.96) | $115,088 | $1,274 | (-$850, $3,985) |
|  | **Only Mortality, Base Case** | 0.86 | (0.70, 0.97) | 1.03 | (1.00, 1.09) | $100,634 | $5,919 | (-$1,058, $19,191) |
|  | **Only Mortality, 50% of Base Case** | 0.93 | (0.85, 0.98) | 1.01 | (1.00, 1.04) | $125,282 | $1,406 | (-$1,904, $6,903) |
|  | **Only Mortality, 25% of Base Case** | 0.96 | (0.92, 0.99) | 1.01 | (1.00, 1.02) | $175,353 | -$703 | (-$2,323, $1,790) |

Abbreviations: eGFR = estimated glomerular filtration rate, UACR = urine albumin to creatinine ratio, ICER = incremental cost-effectiveness ratio, HR = hazard ratio, QALY = quality-adjusted life year, ESRD = end-stage renal disease, CI = confidence interval

Notes:

We summarize each of the scenarios below:

Base case: MDC effectiveness was 25% in CKD stage 3, 50% in CKD stage 4, 100% in CKD stage 5

50%, 25% of base case: MDC effectiveness was 12.5%/6.25% in CKD stage 3, 25%/12.5% in CKD stage 4, 50%/25% in CKD stage 5, respectively

100%, 50%, 25% of non-discounted: MDC effectiveness was 100%, 50%, and 25% in all CKD stages respectively

Only Mortality: Same as above except MDC was only effective in reducing mortality (not progression to ESRD)

* Urine albumin to creatinine ratio in units of mg/g

† Net monetary benefit under a willingness to pay threshold of $150,000 per QALY gained
